# Tangled Tail of Mechanically Interlocked Peptides

**DOI:** 10.1021/jacs.6c05395

**Published:** 2026-06-23

**Authors:** Toby G. Johnson, A. James Link

**Affiliations:** † Department of Chemical and Biological Engineering, 6740 Princeton University, Princeton, New Jersey 08544, United States; ‡ Omenn-Darling Bioengineering Institute, Princeton University, Princeton, New Jersey 08544, United States; § Department of Chemistry, Princeton University, Princeton, New Jersey 08544, United States; ∥ Department of Molecular Biology, Princeton University, Princeton, New Jersey 08544, United States

## Abstract

First synthesized by chemists in the 1960s, mechanically interlocked molecules (MIMs) were created out of scientific curiosity and thought only to be academic oddities. However, naturally occurring interlocked biomolecules were subsequently discovered, highlighting the potential of the mechanical bond to serve a biological function. Interlocked structures have been identified in different classes of biomolecules; for example, catenated DNA serves to regulate DNA transcription; and the capsid of the HK97 bacteriophage consists of 72 protein macrocycles interlocked into a catenated chainmail sphere for the storage of viral DNA. The fields of molecular biology, which focuses on characterizing naturally occurring interlocked biomolecules, and supramolecular chemistry, which strives to chemically synthesize ever more complex molecular topologies, have historically run orthogonally to one another. However, as the conventional subject areas blend into synthetic biology, the benefits of a combined, interdisciplinary approach are evidenced by the bioengineering of new-to-nature mechanically interlocked peptides (MIPs). As the field evolves to attract increasingly diverse and interdisciplinary interest, we feel the time is right to provide an overview of these advances and establish a unified nomenclature. By systematically reviewing the MIPs currently found in nature, and the MIMs synthesized by chemists from peptidyl components, drawing chemical comparisons between the two we hope to bridge the gap between these fields and lay the foundations for future interdisciplinary work.

## Graphical abstract

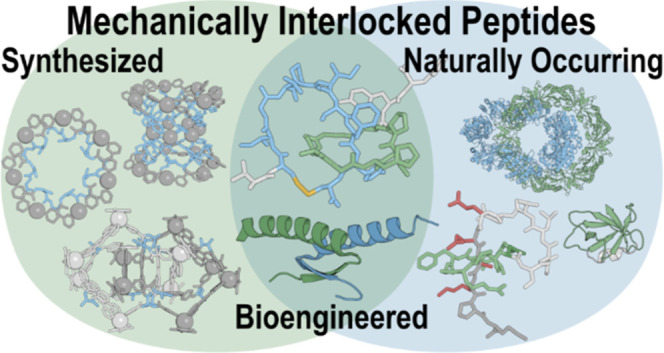

## Introduction

Originally created as an academic curiosity by synthetic chemists, MIMs are a unique class of compounds which are comprised of molecular components threaded through one another, such that the ensemble is unable to separate without breaking a covalent bond.^1^ In the macroscopic world rings made of steel, a strong but brittle material, can be interlocked with one another to form a chain which retains the high tensile strength of steel with the new property of flexibility. Wagon wheels provide another example of interlocked components, where the wheels are threaded onto an axle, but not fixed to it, allowing free rotation for locomotion. In fact, mechanically interlocked components are a prevalent design feature in macroscopic machines, inspiring chemists to design ever more complex interlocked topologies to study and exploit the unique, emergent properties of the resultant assembly.^2–5^ Synthetic MIMs have demonstrated unique benefits when employed for the stabilization of reactive species,^6^ for sensing and catalysis,^7–10^ ion transport,^11–14^ memory storage,^15^ the synthesis of sequence-specific oligomers,^16^ and have already displayed some success in biomedical applications.^17,18^

MIMs can be divided into four categories: knots, catenanes, rotaxanes, and entanglements as shown in Figure 1. Applying mathematical descriptors to these sophisticated entangled architectures has been helpful in providing a framework to define the structures,^19^ however, it has also been shown to trivialize the chemical complexity of them often leading to confused nomenclature. Applying a strict mathematical description of topology to interlocked architectures reduces chemical structures to a so-called embedded graph. An embedded graph is the simplest representation of the connectivity of a molecule when projected onto a surface, where bond lengths and bond angles are not considered and only the connectivity of the architecture is represented. These embedded graphs are helpful to distinguish between topologically complex structures (catenanes and knots), which cannot be represented in two-dimensional space without a crossing point, and topologically trivial structures (rotaxanes and entanglements), which have an embedded graph with no crossing points. So, when it comes to describing interlocked architectures, denoting rotaxanes as complex chemical structures should be avoided, given that they are not topologically complex. Indeed, the term topology should be avoided entirely for rotaxanes as mathematically they possess no topological features. For example, a rotaxane capable of thermally unthreading into the noninterlocked components (referred to as a pseudorotaxane), garners a structure with no crossing points following deformations (topologically trivial). However, in chemistry, physical constraints such as restricted bond rotation and the inability to extend bonds infinitely are the defining properties of molecular species, that enable the isolation of different conformational isomers with unique properties. For example, rotaxanes display unique properties differing from their component parts, despite being equivalent in terms of mathematical topology. That said, applying a rigorous mathematically grounded nomenclature to interlocked molecular systems to maintain continuity between scientific disciplines and to provide a strong grounding for the development of ever more sophisticated architectures is essential.

**Figure 1:**
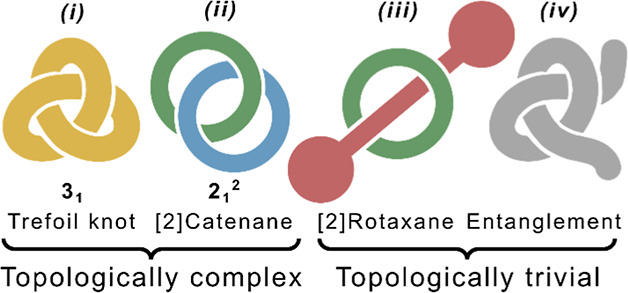
Mechanically interlocked architectures. (i) The trefoil knot (3_1_) is the archetypal knot where one cyclic strand is entangled into a knotted topology that cannot be represented in 2D without crossing points. (ii) The Hopf link (2_1_^2^) is the simplest link (or [2]­catenane) with a crossing number of 2. (iii) A [2]­rotaxane is a mechanically interlocked architecture which kinetically traps components together but topologically is trivial as deformations of the structure can untangle the components. (iv) Molecular entanglements may generate mechanically interlocked architectures but given that the strand can be deformed into a linear chain they are topologically trivial (may sometimes be referred to as open knots). Alexander-Briggs notations are included for the topologically complex architectures.

Historically, the term topology has been used in biology to describe any peptide structure with a different branching pattern or for comparison of an interlocked architecture with the unthreaded conformer.^20–22^ However, in the majority these architectures are in fact topologically trivial and thus should be discussed in terms of constitutional isomerism or alternative conformational arrangement—with no reference to topology at all. When a chain becomes entangled in the macroscopic world like tying a shoelace, we may say it is knotted, however mathematically this is not a knot as a sequence of deformations can return the shoelace to an untangled linear conformation. Cyclizing the tail ends of an entangled strand can result in a true mathematical knot if deformations cannot unravel the structure into a topologically trivial cyclic strand, the so-called unknot. If a single cyclic strand cannot be represented in 2D space without crossing points it is topologically complex and therefore mathematically a knot. The simplest knot is the trefoil knot with 3 crossing points and is referred to as a prime knot, as it cannot be decomposed into component factor knots. A knot is defined as a composite knot if it is comprised of multiple factor knots connected together in the same cyclic strand. When two or more cyclic strands are interlocked with one another this is referred to as a mathematical link, or [*n*]­catenane (where *n* refers to the number of components) in molecular architectures. Structures with linear strands interlocked with cyclic ones that cannot be disentangled without breaking covalent bonds are referred to as [*n*]­rotaxanes (where *n* refers to the number of components) but given that deformations can reduce the structure to a 2D representation without crossing points they are topologically trivial.

The field of knot theory has categorized the simplest knots and links, identifying the complete set of topological isomers for a given crossing number (CN) to which a unique Alexander-Briggs notion can be applied.^23^ The Alexander-Briggs descriptor *x*_*z*_^*y*^ denotes each unique knot or link, where *x* refers to the CN, *y* corresponds to the number of components (in a knot, *y* = 1 and is usually not included), and z is a designator for the order of the structure within the defined set of structures with that specific CN and number of components. The trefoil knot (3_1_) is the only knot with 3 crossings but can exist as one of two topological isomers which are enantiomers. This topological chirality is a result of the structure itself, irrespective of directionality of the component cycle and is therefore said to be an unconditional stereogenic element.^24^ The Hopf link (2_1_^2^) is the simplest mathematical link, which is achiral unless the component rings are oriented generating conditional stereogenic elements. So far, only the simplest systems formed of cyclic or linear components have been considered, however additional connections introduced between or within components can alter the structure and may lead to changes in topological complexity. To address the growing variety of mechanically interlocked molecular architectures, there is a constant need to review the nomenclature to enable the classification and accurate description of structures. Indeed, in the discovery of new MIP architectures, where additional cross-links are more prevalent than synthetic derivatives^25^ a rigorous description of topology is essential, and the use of circuit topology has recently been employed to address this.^26^

Although first synthesized by chemists,^27–29^ MIMs were subsequently found in nature. Clayton and Vinograd identified the first mechanically interlocked biomolecules, in 1967, showing that DNA extracted from HeLa cells and human leukemic leucocytes contained interlocked rings of DNA.^30,31^ Since this seminal work, the function of dedicated topoisomerase enzymes to regulate the extent of DNA catenation (defined by the linking number) has been well studied.^32–35^ Increasing the linking number increases the packing efficiency of supercoiled DNA, while reducing the linking number is necessary to allow the procession of DNA polymerase during DNA replication. Indeed, crossings introduced during DNA replication^36^ must be removed (i.e., separate rings, linking number = 0) before cell division can occur, highlighting the integral biological function of molecular topology in the regulation of gene expression and cell division, two essential processes of a cell. The ease of DNA synthesis and the exploitation of topoisomerases have enabled the preparation of synthetic mechanically interlocked DNA^37,38^ within the field of DNA origami for the development of functional devices.^39,40^ No topologically complex RNA molecules have been discovered, despite secondary structures which lack chain entanglements being referred to as pseudoknots.^41^ Recently, however, an RNA chain was identified with an entangled structure (PDB: 4V2S) but since the chain ends are not linked this is mathematically not a knot.^42^

Polypeptides are another class of biomolecules for which naturally occurring interlocked architectures have been identified and will be the focus of this review. MIPs in nature span a large range of length scales. Peptides (<50 amino acids, aa) have been identified with small 7 aa macrocycles (as small as 22 atoms) threaded by a peptide chain in [1]­rotaxane lasso peptides, resembling the scale of protein secondary structures. Proteins (>50 aa) with an entangled polypeptide backbone form at the tertiary structure level and can become a true knot upon formation of an internal disulfide bond. Quaternary protein complexes have also been discovered with a true interlocked [72]­catenane structure, as well as catenane-type architectures which are not strictly speaking interlocked molecules as they are self-assembled by noncovalent interactions rather than being covalently linked. This perspective aims to clarify the, at times, confusing terminology of entangled polypeptides applying nomenclature from the field of supramolecular chemistry to unify interlocked peptides with MIM nomenclature. Through direct comparison of naturally occurring and synthetic aa containing MIMs, links between the size, chemical functionalities and chirality of these two molecular classes will be drawn to provide opportunities for future research. The design of new-to-nature MIPs through bioengineering will also be surveyed, highlighting the most recent advances which garner new architectures and study molecular motion, helping to answer the question of the biological function of the mechanical bond in living systems. Bioengineered MIPs, which inherently are produced within the cell, is one of the most promising areas in which we can expect to see useful applications of biocompatible MIMs that will have impacts on biology and medicine.

MIP architectures, regardless of length scale, are typically found to show minimal dynamic motion. In contrast, the design of synthetic MIMs including those bearing peptidyl components has been significantly motivated by the propensity to display unique molecular motions as a result of confinement introduced by the interlocked architecture. Notably, MIMs underpin the field of molecular machines (Nobel Prize in Chemistry 2016,^43–45^ for molecules with controlled nanomechanical motion, which can perform a task when energy is added) and as such MIPs have great potential to garner a new class of biocompatible molecular machines. Through this perspective, we hope to untangle some of these topics and present a unified view of the field of mechanically interlocked peptides without trivializing key details, so that chemists and biologists alike can engineer new complexity into these versatile and biologically relevant architectures.

## Entanglements and Knots in Polypeptides

### Naturally Occurring Entangled Proteins

The field of knotted proteins adds complexity to the matter given that this area refers to any protein with a chain entanglement of the peptide backbone as a knot (Figure 2),^46^ like the so-called knots in a shoelace. However, topologically these are not knots, as deformations of the structure can reduce these entanglements to a linear chain. In 1977, Richardson identified the first protein chain entanglement in the structure of human carbonic anhydrase C which had been solved 5 years before.^47,48^ Many other entangled proteins have since been discovered and comprise ∼6% of the total number of structures within the Protein Data Bank (PDB).^49^ The KnotProt database was established to catalogue these structures and provides an excellent resource for the characterization of protein entanglements.^50,51^ With the advent of machine learning (ML) models for the prediction of protein 3D structures, the same algorithms applied to the PDB have been applied to predicted protein structures to identify chain entanglements with the results collated in the AlphaKnot database.^52,53^ The Alexander-Briggs notation is applied to these linear chain-entangled proteins, referred to as probabilistic knots. Despite not being knots, the mathematical descriptor can be useful to classify these structures. Of the entangled proteins, the vast majority are enzymes with the active site found within the knotted core, highlighting the benefit of the entanglement to facilitate the desired arrangement of residues for catalytic activity.^49^ The highest CN in an entangled protein was previously 6, observed in the dehalogenases DehI from *Pseudomonas putida* (PDB: 3BJX) which has a stevedore-type (6_1_) architecture (Figure 2ii).^54,55^ However, the septafoil torus-like (7_1_) architecture of Q9PR55 (Figure 2iii), an 89 aa protein from *Ureaplasma urealyticum*, first identified from AlphaFold 2 predictions was recently confirmed by X-ray crystallography (PDB: 8IWA).^56,57^ The interested reader is directed to the following reviews for a more detailed discussion of entangled proteins.^41,49,58–60^ Entanglements between multiple chains can influence the properties of higher order molecular assemblies and has been shown to influence the assembly of biomolecular condensates.^61–63^

**Figure 2:**
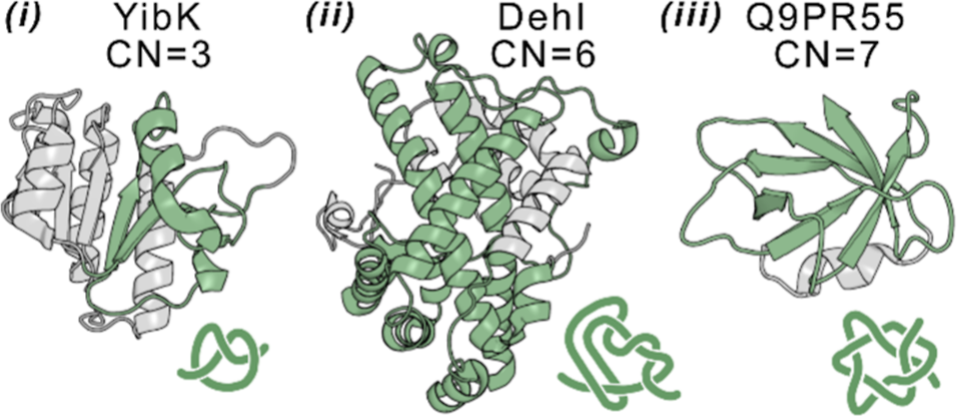
Entangled proteins. Crystal structures showing the knotted core (green) of open chain entangled proteins (i) YibK (PDB: 1MXI), (ii) DehI (PDB: 3BJX) and (iii) Q9PR55 (PDB: 8IWA). Schematics of the entangled structures are shown to convey the architecture of the knot core, although all structures are topologically trivial. The crossing number (CN) in entangled protein structures conveys the number of crossing points in a projection of the polypeptide chain onto a 2D plane.

Covalently cross-linking the tail ends of an entangled protein can generate a topologically nontrivial mathematical knot, referred to by biologists as a deterministic knot.^50^ However, as is common in proteins multiple internal cross-links can be formed (disulfide bonds or coordinated metal ions) which generate various closed loops that may interlock with one another forming topologically nontrivial structures termed θ-curves.^64–67^ The true topological complexity of these structures is only just beginning to be unraveled, but is sure to yield interesting results for the biologists and mathematicians alike. So far much effort has gone into deciphering the folding mechanism of entangled proteins^68–70^ and to determine the influence of entanglement on physical properties,^71,72^ however, much is yet to be learnt about the link to function.^49,73^ Systems have been bioengineered to probe the properties of entangled proteins, with cysteines substituted into the structure to generate trefoil-based entangled polymer chains^74^ and to form a true composite knot linking together two trefoil-based entanglements (3_1_#3_1_).^75^ The entangled protein YibK from *Pseudomonas aeruginosa* has an open chain trefoil-like structure (Figure 2i), which could be enzymatically cyclized with sortase A to give a true trefoil knot (3_1_).^76^ Cyclic YibK (cYibK) was crystallized (PDB: 6QH8) to confirm the trefoil knot structure, which consists of an all amide linked 162 aa peptide backbone. Beyond the entangled motifs found in nature, new structures have been engineered de novo with trefoil (3_1_) and pentafoil (5_1_) type entanglements^77–79^ expanding the motifs available for future applications of entangled proteins.

### Synthetic Molecular Knots Containing Amino Acids

The first molecular knot synthesized was a trefoil knot reported by Dietrich-Buchecker and Sauvage in 1989 ^80^ employing his now well-established Cu^I^-phenanthroline metal ion template-directed strategy.^81^ Copper coordination by a bisphenanthroline ligand generated a well-defined helicate which upon ring closing yielded a trefoil knot, [2]­catenane and macrocycle with the former isolated in 3% yield. Metal ion template-directed synthesis of MIMs is by far the most common strategy to preorganize the chain into an entangled architecture before ring closing, however other noncovalent interactions have been shown to effectively stabilize the entanglement prior to cross-linking.^82,83^ In 2012, Sanders et al. demonstrated for the first time that a molecular knot could be formed without the need for metal ion templation, instead self-assembling due to the hydrophobic effect in water. The trefoil knot was the major thermodynamically preferred product of a disulfide dynamic combinatorial library (DCL) formed by a trimeric naphthalenediimide ligand containing interstitial alanine residues and terminal cysteine amino acids. When the ligand comprised of all l- or d-amino acids equal and opposite enantiomers of the trefoil knot were obtained, but if a ligand with both l- and d-amino acids was used no trefoil knot was observed in the DCL. Expanding on this design, Sanders et al. were able to identify figure-of-eight knots (4_1_) and a Solomon link (4_1_^2^) in a DCL formed from a dimeric naphthalenediimide ligand connected by a flexible alkyl chain and terminated with cysteines as before to facilitate disulfide formation.^84^

Amino acids are readily accessible from the chiral pool of enantiopure natural products, making them ideal building blocks for the preparation of topologically chiral molecular knots. The chirality of the fixed stereogenic center of the aa was shown by Sanders to influence the enantiomer of the trefoil knot obtained, but the crossing pattern of the knot topology could not be unambiguously assigned. X-ray crystallography allowing the absolute assignment of topological chirality of the aa containing trefoil knot **1a** (The Cambridge Crystallographic Data Centre (CCDC) deposition number: 600890), which was formed by ring closing a linear valine-steroid trimeric precursor (Figure 3A).^85^ Leigh and co-workers demonstrated that two peptide β-hairpin (4 aa) motifs could be integrated into a tritopic-2,6-pyridinedicarboxamide ligand, to form a trefoil (3_1_) knot templated by Lu^III^.^86^ Zhang and co-workers have also recently demonstrated an elegant method to stereoselectively prepare cinquefoil knots (5_1_), where the stereogenic center of pendant amino acids dictate the crossing pattern of the assembly and hence the unconditional topological chirality of the resultant knot.^87–89^ The method has been expanded to demonstrate that the aa induces narcissistic chiral self-sorting, where helicates preferentially form from ligands baring the same pendant aa of the same point chirality.^90^

**Figure 3:**
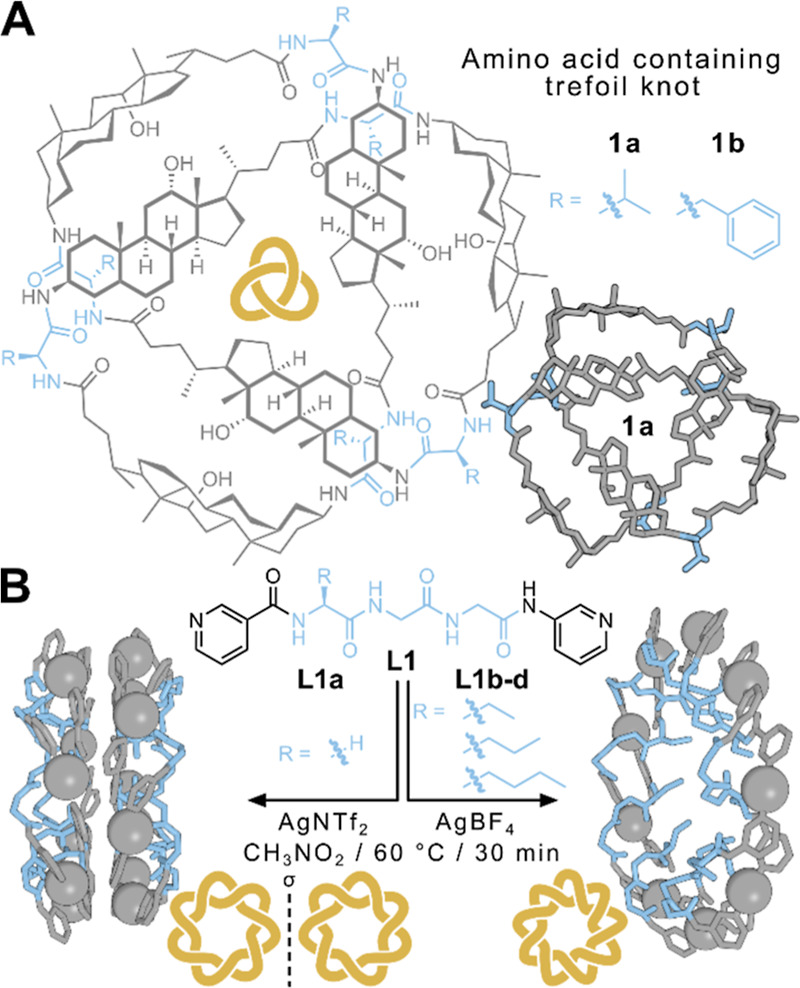
Synthetic knots containing amino acids (blue). (A) Chemical structure of **1** with different aa residues and crystal structure of **1a** (CCDC: 600890) showing the crossing pattern of the single enantiomer of the trefoil knot synthesized. (B) Chemical structure of **L1** with different aa residues used to form metal-peptide complexes with different knot architectures. Ligand **L1a** with a Gly_3_ sequence formed an enantiomeric pair of septafoil torus knots (CCDC: 1953459) (left), while **L1b-d** formed a single enantiomer of a nonafoil torus knot with the structure formed from **L1b** shown (CCDC: 2100940) (right).

In 2014, Fujita reported an entangled metal-peptide network formed by coordination of Ag^I^ with short tripeptide linkers with 3-pyridyl amide ligands at both termini (3pyr-Gly-Pro-Pro-3pyr).^91,92^ Iterations of the peptide linker have been shown to modulate the metal-peptide network structure and also generate discrete coordination complexes with complex topologies.^93^ Remarkably, the ligand **L1a** with a simple Gly_3_ linker was found to form a 7-crossing torus knot (7_1_) in the solid state (CCDC: 1953459) when complexed with AgNTf_2_ (Figure 3B).^94^ The 7_1_ knot can exist in two enantiomeric forms as a result of the crossing pattern (unconditional topological stereogenic element). Since the knot is composed of seven subunits, each with strand directionality, there are 10 potential constitutional isomers that arise from arranging the subunits in different orientations end-to-end. With the unconditional topological stereogenic element of the knot and the conditional stereogenic element introduced by strand directionality, each of the 10 constitutional isomers can exist as 4 possible stereoisomers. In the solid state the all head-to-tail isomer was resolved as a pair of topological enantiomers. Changing the first aa from glycine to alanine of **L1** was found to generate a single enantiomer of the septafoil knot in the solid-state, while increasing the length of the alkyl side chain (ethyl, *n*-propyl, or *n*-butyl) was found to increase the CN of the knot or link observed in the solid state, with the structure of a nonafoil torus knot (9_1_) confirmed by X-ray crystallography (CCDC: 2100940) (Figure 3B).^95^ Expanding on this assembly, a noncanonical aa l-β-(4-pyridyl)­alanine (4pa) could be incorporated within a ligand (3pyr-Gly-4pa-Pro-3pyr) which assembled into a tubular torus knot dimer (CCDC: 2418832) formed from two 7_1_ torus knot bridged by 7 pyridyl cross-links at the center of each ligand.^96^ These examples highlight that relatively simple aa building blocks can direct the formation of structures with remarkable topological complexity.

## Protein Catenanes

### Catenanes in Naturally Occurring Polypeptides

Catenanes have been identified in nature, spanning a wide range of molecular length scales.^17,97–99^ Large self-assembled multiprotein complexes have been characterized with a Hopf link [2]­catenane-like architecture (Figure 4). Although not strictly mechanically interlocked as they are self-assembled via noncovalent interactions rather than covalently linked, the architecture has been observed for several enzymes from different organisms suggesting its physiological relevance. First identified in 2005 in the crystal structure of bovine mitochondrial peroxiredoxin III (PrxIII) (PDB: 1ZYE and 4MH2),^100,101^ Hopf link-like protein assemblies have since been observed in CS_2_ hydrolases from archaea (PDB: 3TEO)^102–105^ and bacteria,^106^ for ribonucleotide reductase (RNR) (PDB: 4ERP) from *Escherichia coli* ^107^ and the recombination protein R (RecR) (PDB: 1VDD) from bacteria which is implicated in homologous recombination to repair single and double-strand breaks in DNA.^108^ A similar Hopf link-like architecture can arise when two C-shaped proteins assemble into an entangled protein dimer (Figure 5). The catenane-like conformation is thought to serve a regulatory function, as the entangled dimer is inactive in the case of the lytic transglycosylase SltB1 from *Pseudomonas aeruginosathis* (PDB: 5O8X),^109^ and active in the methyltransferase OphMA from *Dendrothele bispora* (PDB: 6MJF).^110,111^

**Figure 4:**
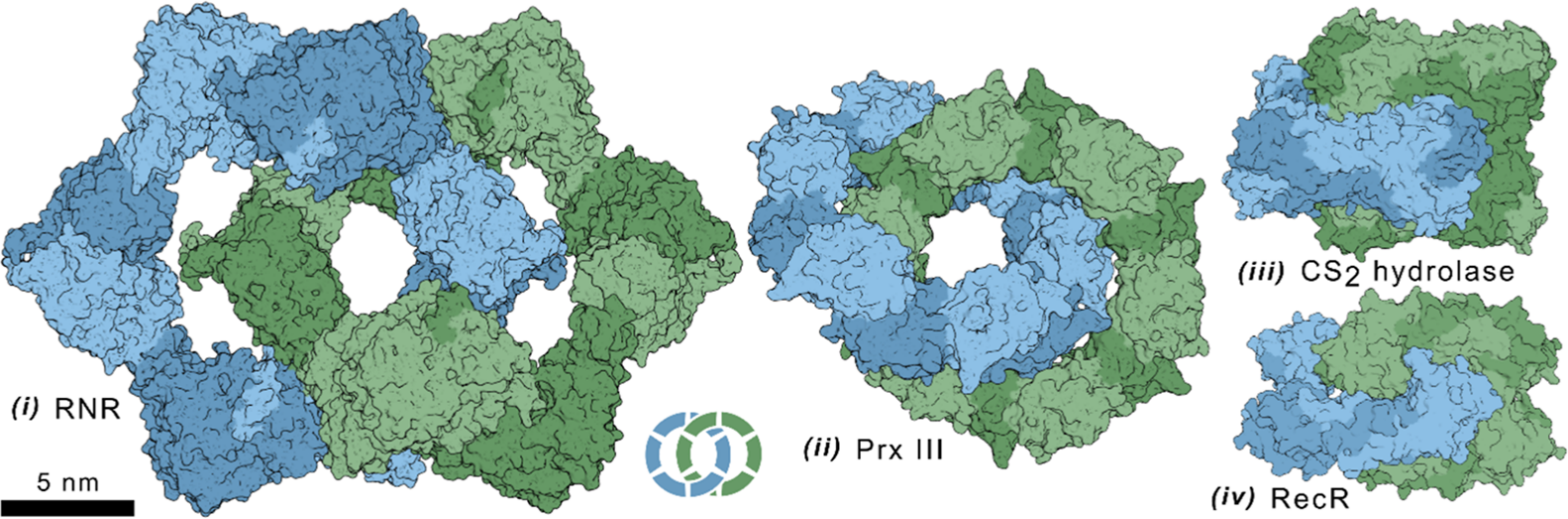
Noncovalent catenane-like protein assembly crystal structures. (i) *E. coli* class Ia ribonucleotide reductase (PDB: 4ERP) with an eight-protein component self-assembled ring. (ii) Bovine mitochondrial Cys168Ser variant of peroxiredoxin III (PDB: 1ZYE) with a 12-protein component self-assembled ring. (iii) *Acidianus* CS_2_ hydrolase (PDB: 3TEO) with an eight-protein component self-assembled ring. (iv) *Deinococcus radiodurans* RecR (PDB: 1VDD) with a four-protein component self-assembled ring.

**Figure 5:**
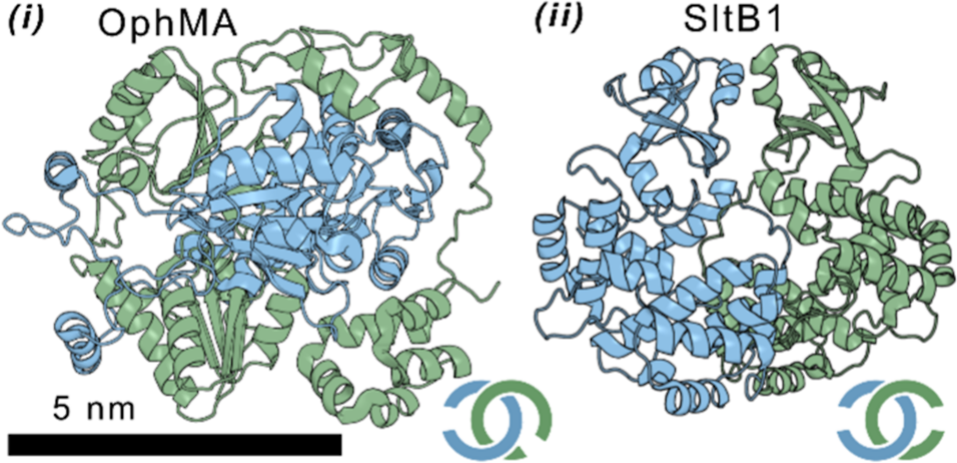
Noncovalent catenane-like protein dimer crystal structures. (i) *Dendrothele bispora* methyltransferase OphMA (PDB: 6MJF) and (ii) *Pseudomonas aeruginosa* lytic transglycosylase SltB1 (PDB: 5O8X).

A true interlocked architecture has been observed in the HK97 bacteriophage capsid,^112,113^ which stores viral DNA. The crystal structure was solved for the remarkable [72]­catenane structure (Figure 6i), which self-assembles from 420 protein subunits into an icosahedral capsid that autocatalytically form 420 isopeptide bonds to garner an interlocked chainmail-like cage (940 and 1128 aa macrocycles, PDB: 1OHG).^112,114^ The ability to express the protein subunit and tailoring protease in *E. coli* and conduct HK97 capsid maturation in vitro has enabled numerous studies of variants to be conducted allowing the stages of capsid maturation to be biochemically analyzed and intermediate structures to be determined by cryogenic electron microscopy, X-ray crystallography and small-angle X-ray scattering (SAXS).^115–123^ Recently, one other capsid has been shown to form a similar interlocked chainmail structure from the *P. aeruginosa* phage D3.^124^ Both the HK97 capsid (Triangulation number, *T* = 7) and D3 capsid (*T* = 9) have icosahedral symmetry, however the D3 capsid was found to have a larger structure comprised of 540 protein subunits that garners a [92]­catenane after autocatalytic isopeptide bond formation. These icosohedral structures differ in their Goldberg triangulation number which defines the number of triangular facets the component 20 equilateral triangular faces of an icosahedron are divided into. Other phage capsids form entangled assemblies stabilized by noncovalent interactions and additional auxiliary stabilizing proteins but lack the covalent linkages and thus are not topologically complex.^125–127^ Engineering the protein subunits of phage capsids to contain cysteine residues, and chemically treating assembled capsids has been used to introduce covalent cross-links, however without structural validation it is unknown whether a catenane architecture was formed.^128–130^

**Figure 6:**
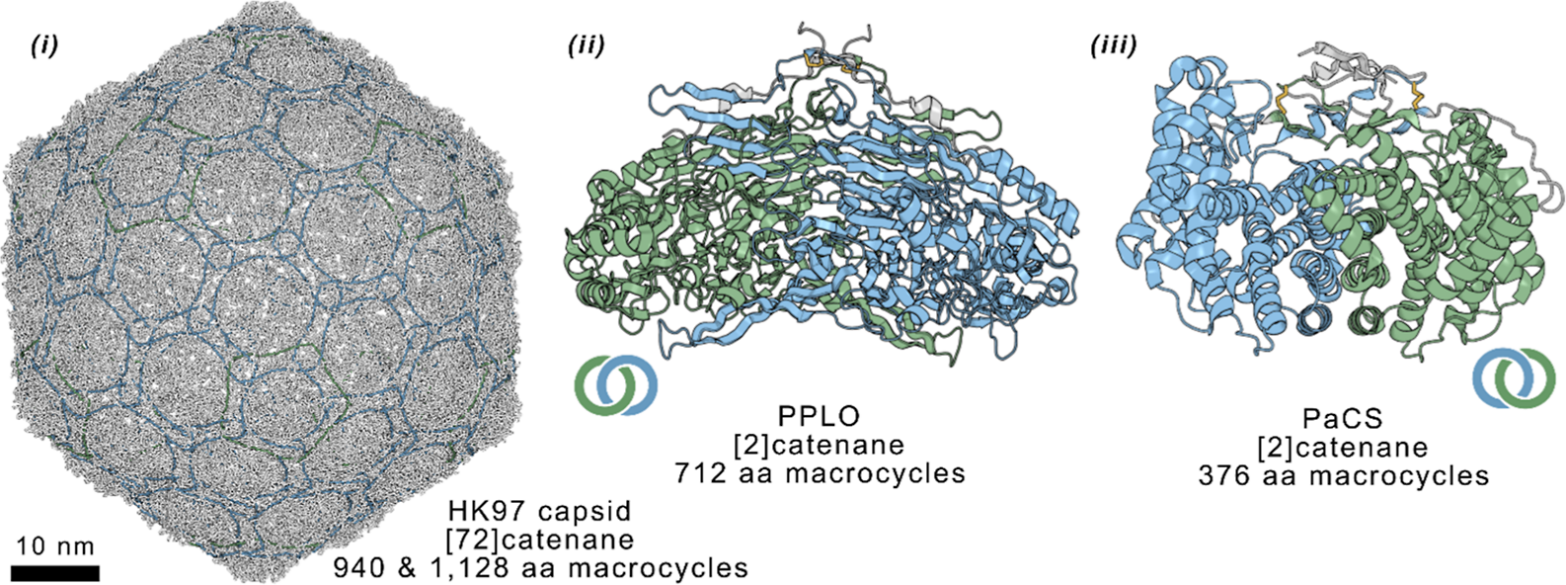
Naturally occurring protein catenanes. (i) HK97 bacteriophage capsid [72]­catenane (PDB: 1OHG), showing the hexameric (1128 aa, blue) and pentameric (940 aa, green) rings partially colored to convey the shortest distance between isopeptide bonds, with scale bar. (ii) *Pichia pastoris* lysyl oxidase (PPLO, 712 aa macrocycle, PDB: 1N9E) and (iii) *Pyrobaculum aerophilum* citrate synthase (PaCS, 376 aa macrocycle, PDB: 2IBP) are protein [2]­catenanes formed by intramolecular disulfide bonds (golden) cyclizing the two protein subunits, to highlight the separate rings each is uniquely colored (blue or green).

The entanglement of chains in protein dimers into intertwined architectures is a common feature observed in protein complexes.^131,132^ Many enzymes function as homodimers, where chain entanglements can result from portions of one protein binding to the other and vice versa in so-called domain swapped dimers.^133,134^ The presence of disulfide linkages in these intertwined protein dimers can result in naturally occurring Hopf link [2]­catenanes such as in the thermophile *Pyrobaculum aerophilum* citric synthase (PaCS, 376 aa macrocycle, PDB: 2IBP)^135^ and *Pichia pastoris* yeast lysyl oxidase (PPLO, 712 aa macrocycle, PDB: 1N9E)^136^ shown in Figure 6. Interestingly, a mutant of PaCS with the two cysteine residues substituted to serines (incapable of forming a [2]­catenane) displayed a melting temperature 10.5 °C lower than the wild-type PaCS, revealing the increased stability afforded by cross-linking in the interlocked topology. The introduction of cross-links (disulfide or ion coordination) within a single protein can also generate [2]­catenanes with different topologies including Hopf Links (CN = 2) and Solomon links (CN = 4).^64,137^ The LinkProt database was established to catalogue catenane-type structures found in proteins, based on self-assembled macromolecular structures, as well as catenanes formed by multiple or single proteins.^138^

### Bioengineering Polypeptide Links

Intertwined protein architectures have been utilized to bioengineer complex protein topologies that have been investigated in biomedical applications.^98,139–141^ Yan and Dawson pioneered this strategy, reporting in 2001 the first synthesis of a [2]­catenane comprised solely from amino acids (40 aa macrocycle).^142^ The linear peptide was prepared by solid phase peptide synthesis (SPPS) containing the dimerization domain from the tumor suppressor protein p53 (residues 325–356), which was known to self-assemble into a bisecting U motif (Figure 7A).^143,144^ Following self-assembly in aqueous buffer, ring closing proceeded by native chemical ligation (NCL) to garner a [2]­catenane following a template-directed synthesis (Figure 7Bi). Introducing M340E/L344 K substitutions into the p53 dimerization domain garnered a [2]­catenane less prone to aggregation in solution, allowing the thermal stability of the assembled intertwined dimer to be directly compared to the [2]­catenane revealing a 59 °C increase in melting temperature as a result of the complex topology.^145^ Substitution of noncanonical amino acids into the p53 dimerization domain allowed the threading of a linear domain into a cyclized version to be monitored by fluorescence spectroscopy. Along with the monitoring of the pseudo[2]­rotaxane assembly, a hetero[2]­catenane was also prepared from a linear and cyclic component providing further evidence for an interlocked structure, where threading must occur prior to cyclization.^146^

**Figure 7:**
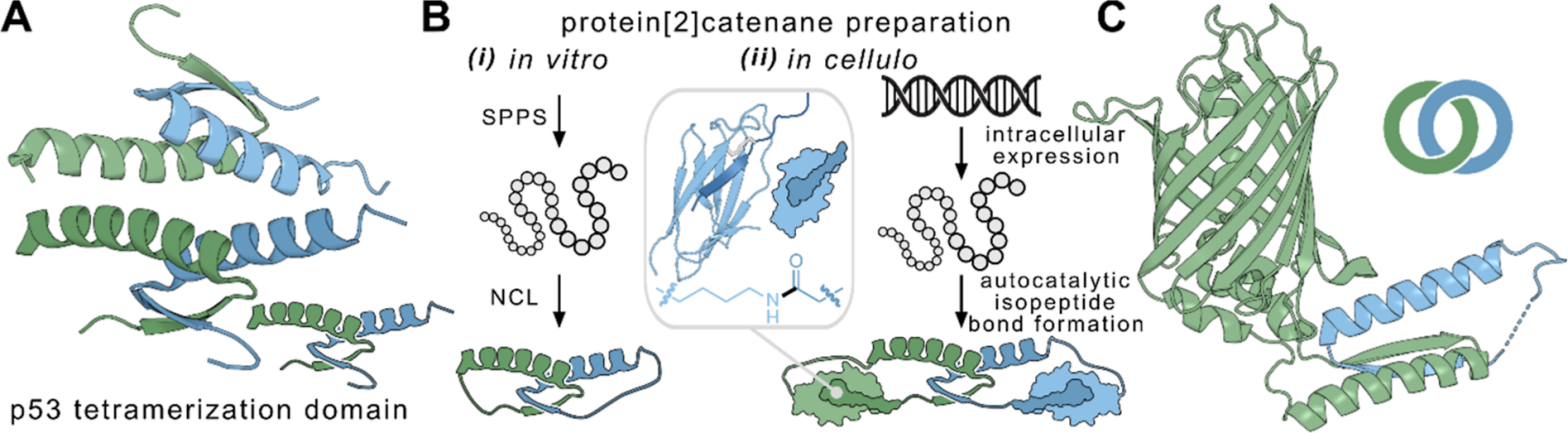
Bioengineering polypeptide links. (A) Crystal structure of the naturally occurring intertwined tetramerization domain from the tumor suppressor protein p53 (residues 325–356, PDB: 8UQR). (B) Template-directed synthesis of protein [2]­catenanes, (i) cross-linking by native chemical ligation in vitro or (ii) autocatalytic isopeptide formation (SpyCatcher/SpyTag, PDB: 4MLI) in cellulo. (C) Crystal structure of a GFP hetero[2]­catenane (PDB: 7BWN).

In 2016, Wang and Zhang expanded on this strategy by utilizing autocatalytic isopeptide bond formation between SpyTag and SpyCatcher to cyclize the self-assembled protein dimer allowing [2]­catenane synthesis in cellulo (Figure 7Bii).^147^ SpyTag is a 13 aa peptide that binds to the ∼12 kDa SpyCatcher protein. Upon this association an isopeptide bond is formed, covalently joining the two polypeptides. A fusion protein was designed with a central p53 dimerization domain flanked on both or one side by an elastin-like protein (ELP) spacer and terminated by appropriate portions of the SpyCatcher/SpyTag system.^148,149^ The fusion protein could be gene-encoded, allowing the transformation of *E. coli* cells with a plasmid to facilitate intracellular expression of the target protein, self-assembly and subsequent bond formation to generate a protein homo[2]­catenane (179 or 255 aa macrocycles).^147^ Replacing the ELP domain in the gene with a protein of interest (POI, either green fluorescent protein (GFP) or dihydrofolate reductase (DHFR)) allowed the preparation of [2]­catenanes and [1]­catenanes (so-called pretzelanes) of the POI in cellulo, which displayed enhanced stability and increased catalytic activity in the case of DHFR relative to cyclic and linear derivatives.^150,151^ Still employing the p53 dimerization domain, but instead utilizing split inteins to facilitate cyclization, a protein hetero[2]­catenane (46 and 280 aa macrocycles) with GFP in one macrocycle could be prepared in cellulo (Figure 7C), with the interlocked architecture confirmed by X-ray crystallography (PDB: 7BWN).^152^ Introduction of charged residues in p53 yielded two domains that preferentially formed a heterodimer, enabling the preparation of higher order hetero­[*n*]­catenanes (*n* = 2, 3 or 4).^153^ The in vivo pharmacokinetic properties of hetero­[*n*]­catenanes have been investigated in mouse models as potential cancer therapeutics.^153,154^ Remarkably, with a cancer biomarker targeting protein (AffiHER2) integrated, [3] and [4]­catenanes displayed enhanced tumor targeting and retention compared to a monomeric control, while a [2]­catenane with interferon-α2b (IFN) mechanically interlocked with an albumin-binding domain (ABD) displayed similarly enhanced stability along with antitumor efficacy. Zhang and co-workers have also prepared mechanically interlocked hydrogel networks from the p53 dimerization domain.^155–157^ The hydrogels showed enhanced swelling ratios and relaxed faster after applied stress because of chain entanglement and could be used as a matrix to grow cells in or for the storage and release of enzymes.

The discovery of new entwined domains in nature, referred to as probabilistic links by biologists,^138^ will enable the development of future MIPs. Although the naturally occurring p53 ^142^ and HP0242 ^77^ dimerization domains have allowed access to many interlocked architectures, models that identify the bisecting U-motif in crystal structures in the PDB^138,158^ or from machine learning protein models^159,160^ will expand the toolbox of MIP bioengineering. Some of these newly identified entwined homo- and heterodimerization domains have been experimentally validated to form a [2]­catenane after cross-linking.^161,162^ Another means of accessing protein catenanes has been to ‘rewire’ the connectivity of individual secondary structure elements in a protein and split the structure into separate chains. These two protein halves then assemble into the same original tertiary structure, but with a new connectivity, such that appropriate cross-linking generates an interlocked architecture. Different interlocked architectures have been prepared in this way, a [2]­catenane was prepared from DHFR,^163^ [2]­rotaxanes and [2–3]­catenanes were prepared from fluorescent proteins,^164^ while [1–2]­rotaxanes, [2–3]­catenanes and a trefoil knot were prepared from the SpyCatcher/SpyTag complex.^165^ Employing a rewired split SpyCatcher/SpyTag complex interlocked proteins can be prepared via an active template-directed synthesis,^166^ where the template facilitates both the organization of components and cross-linking such that formation of an interlocked architecture is favored over the topologically trivial product. This active template-directed approach does not require an entwined dimerization domain, enabling more complex catenanes to be accessed with a series of radial [*n*]­catenanes (*n* = 2, 3, 4 and 5) prepared with one central ring^167^ and a linear [5]­catenane with all unique macrocycles reported (so-called a molecular olympiadane).^168^ To confirm the interlocked protein architecture crystallography remains the best method, however selective proteolysis of component macrocycles and ion mobility spectrometry-mass spectrometry (IMS-MS) have also been employed.^169^

Catenanes have also been prepared in vitro from lasso peptides (Figure 8). Lasso peptides will be discussed in more detail below, but for now suffice it to say that lasso peptides are small (13–33 aa) [1]­rotaxane peptides produced by bacteria. Our group first demonstrated in 2016 that a lasso peptide derivative could form a disulfide DCL (mediated by Cys cross-linking) in water.^170^ The lasso peptide with 3 point substitutions (2 Cys and 1 Arg) could be heterologously expressed in *E. coli*, and following isolation and treatment with trypsin (proteolysis after Arg) garnered the [2]­rotaxane used for the DCL. The library was comprised of ∼50% [3]­catenane (8 and 18 aa macrocycles, Figure 8ii) and ∼25% of each of the two [4]­catenane (8 and 27 aa macrocycles) constitutional isomers, that result from different head-to-tail arrangements of the [2]­rotaxane subunit. Expanding on this work, in 2021 our group demonstrated that by changing the position of the cysteine/s substituted within the lasso peptide scaffold a range of different interlocked architectures could be accessed in the disulfide DCL.^171^ MIPs include [1–3]­rotaxanes, molecular [c2]­daisy chains, a lasso peptide with non-native chirality, [2–5]­catenanes and a double-lasso macrocycle were all identified, with the NMR structure solved for the peptide [2]­catenane (Figure 8i). Interestingly, the molecular size of the peptide [2]­catenane (8 and 10 aa, 26 and 32 atom macrocycles) is nearly equivalent to Sauvage’s original copper-templated [2]­catenane^172^ (30 atom macrocycles) and Stoddart’s donor–acceptor cyclophane-based [2]­catenane^173^ (28 and 34 atom macrocycles).

**Figure 8:**
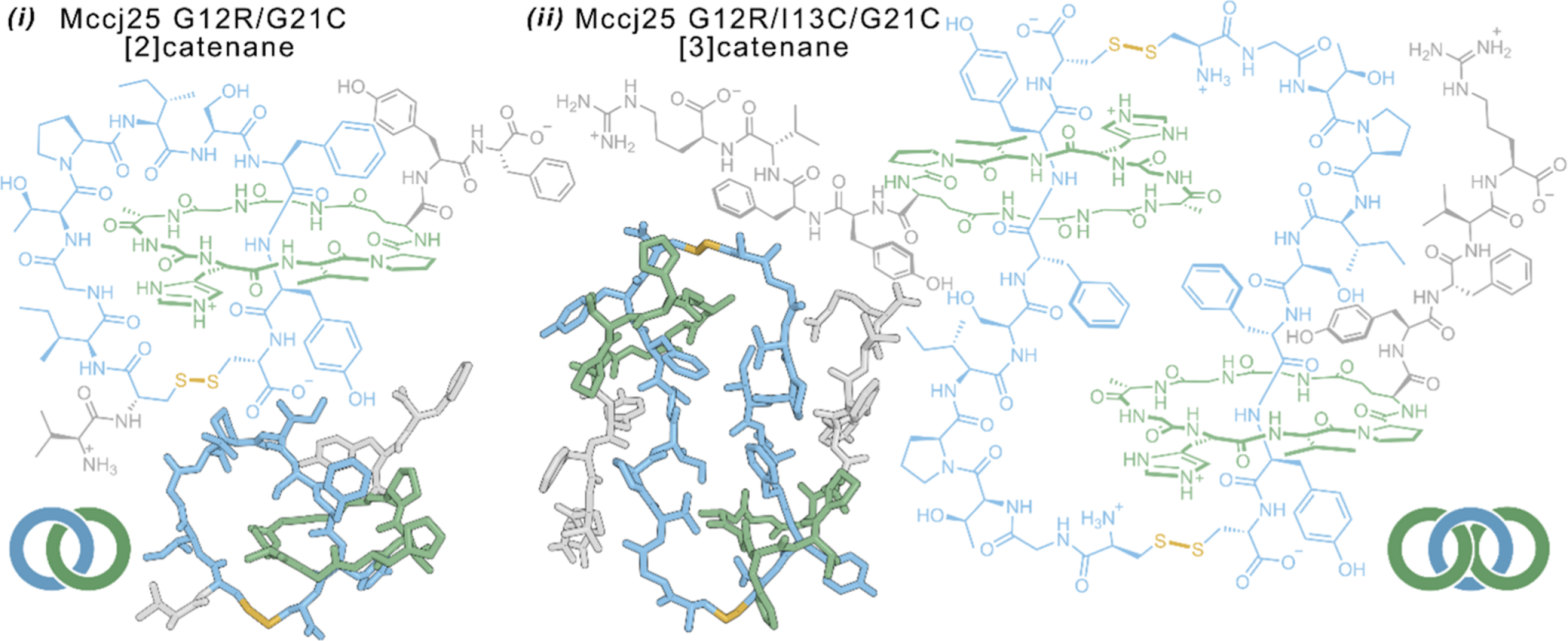
Mechanically interlocked peptide catenanes. Chemical structure and solution state structures determined by ^1^H NMR spectroscopy of (i) a [2]­catenane (8 and 10 aa macrocycles, PDB: 7LL7) and (ii) [3]­catenane (8 and 18 aa macrocycles, PDB: 5T56) formed by intramolecular disulfide cross-linking in a lasso peptide (MccJ25) derivative.

### Synthetic Catenanes Containing Amino Acids

Catenanes have been a desirable target for synthetic chemists, not only for their aesthetically intriguing structure but for the hope that their topologically complex architecture would provide unique properties.^174–176^ First synthesized in 1960, a [2]­catenane was prepared by Wasserman based on the statistical entanglement of alkyl chains in a yield of ca. 0.0001%.^27^ Metal template-directed strategies first developed by Sauvage significantly improved the yield of complex architectures,^172^ and more recently the development of active metal template-directed strategies pioneered by Leigh and co-workers have become the standard protocol for the preparation of MIMs.^177,178^ The integration of amino acids into catenanes as simple linker molecules is common and beyond the scope of this perspective.^179–181^ However, examples in which the aa plays a significant role in the interlocked architecture will be surveyed.

In 2005, Sanders, Otto and co-workers reported a DCL formed from the dipeptide-hydrazide building block **L2a** that favored assembly of a [2]­catenane in the presence of the neurotransmitter acetylcholine (Figure 9i).^182^ The acylhydrazide (C-terminus) and 4-(dimethoxymethyl)­benzoate unit (N-terminus) reversibly combined to form hydrazone linkages under acidic conditions in organic solvent. In the absence of acetylcholine, no catenane products were observed in the DCL, but when added, a [2]­catenane consisting of two interlocked macrocyclic trimers was formed as the major product (70%), with the structure determined by ^1^H NMR. Gagné and co-workers expanded on this design investigating a series of building blocks with other noncanonical amino acids in the place of Phe to garner [2]­catenanes in the absence of a templating additive and confirming the architecture by X-ray crystallography (Figure 9ii).^183–185^ Sanders, Pantoş and co-workers have also demonstrated numerous examples of disulfide DCLs in water comprised of donor and acceptor components terminated by cysteines to facilitate disulfide formation contain [2–3]­catenane members.^84,186–194^

**Figure 9:**
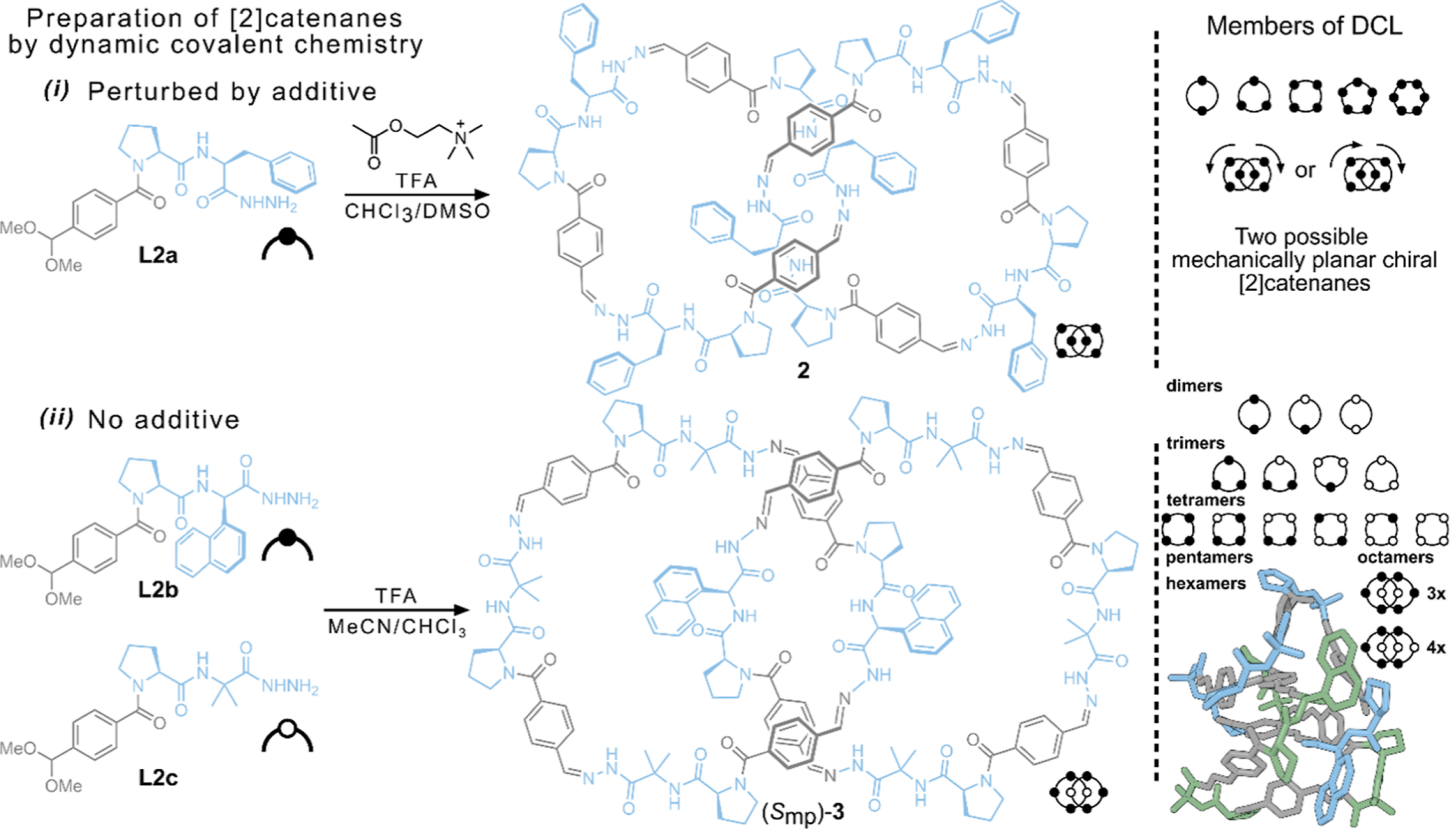
Catenanes containing amino acids formed by dynamic covalent chemistry. (i) Sanders’s preparation of [2]­catenane **2** formed as the major product in a DCL in the presence of acetylcholine. (ii) Gagné’s preparation of [2]­catenane (*S*_mp_)-**3** with the solid-state structure confirmed by X-ray crystallography. One of the two possible diastereomers of the mechanically planar chiral configuration of the [2]­catenane is resolved in each of the DCLs with Gagné obtaining a crystal structure (CCDC: 736965) to confirm the absolute chirality.

Ulrich, Cougnon and co-workers recently reported a small cationic [2]­catenane with a peptide portion in the macrocycle that was prepared via dynamic covalent self-assembly in water.^195^ Condensation of a bisaldehyde that self-assembles into a bisecting U-architecture through hydrophobic interactions with a short peptide bearing acylhydrazide moieties facilitated the synthesis of the [2]­catenane. The presence of arginine residues enabled the [2]­catenane to complex siRNA and reduced the nuclease susceptibility of the RNA. The interlocked architecture also displayed enhanced proteolytic stability to trypsin and proteinase K relative to the component linear peptide.

The reappreciation of stereogenic elements that arise from an interlocked architecture has led to a flurry of stereoselective synthesis of MIMs spearheaded by Goldup and co-workers.^196,197^ Three conditional mechanical stereogenic units can be defined for catenanes, arising when two oriented rings (mechanically planar chirality), two facially dissymmetric rings (mechanically axial chirality), or one oriented and one facially dissymmetric ring (mechanical geometric isomerism) are interlocked (Figure 10A). These descriptors can also be applied to rotaxanes, which are related to the catenane architectures by appropriate ring opening of one component. In order to access such structures Bordoli and Goldup developed a chiral auxiliary strategy to stereoselectively prepare a single diastereomer (one stereogenic center and one mechanical stereogenic element) that after ablation of the auxiliary yielded a single enantiomer resulting from mechanical stereochemistry.^198^ After the initial report using a sugar as the chiral auxiliary, it was shown that a Phe-based azide gave mechanically planar chiral [2]­rotaxanes in up to 98:2 d.r. via active template Cu-mediated alkyne–azide cycloaddition (AT-CuAAC) reactions.^199–203^ Mechanically planar chiral catenanes have been prepared from an oriented linear component and an oriented macrocycle with a pendant chiral auxiliary (Figure 10B).^204^ Utilizing a pendant Phe as the chiral auxiliary allowed the stereoselective synthesis of a [2]­catenane,^205^ as well as all three stereoisomers of a coconformationally mechanically planar chiral [3]­rotaxane by judicious selection of the chirality of the aa.^206^ Incorporating the stereogenic center within the ring also enabled access to mechanically planar chiral [2–3]­catenanes, where a Tyr-based azide provided stereoselective control to the AT-CuAAC reaction.^207^ The rich stereochemistry of these derivatives highlights the stereogenic elements that can result from the arrangement of MIMs, however none of them strictly result from the architecture’s topology as orientation of the macrocycles is not a topological feature and hence are said to have conditional mechanical stereochemistry. Easy access to enantiopure aas has made them essential building blocks in forging mechanical stereogenic elements, and they will likely continue to be utilized in the future stereoselective preparations of MIPs.

**Figure 10:**
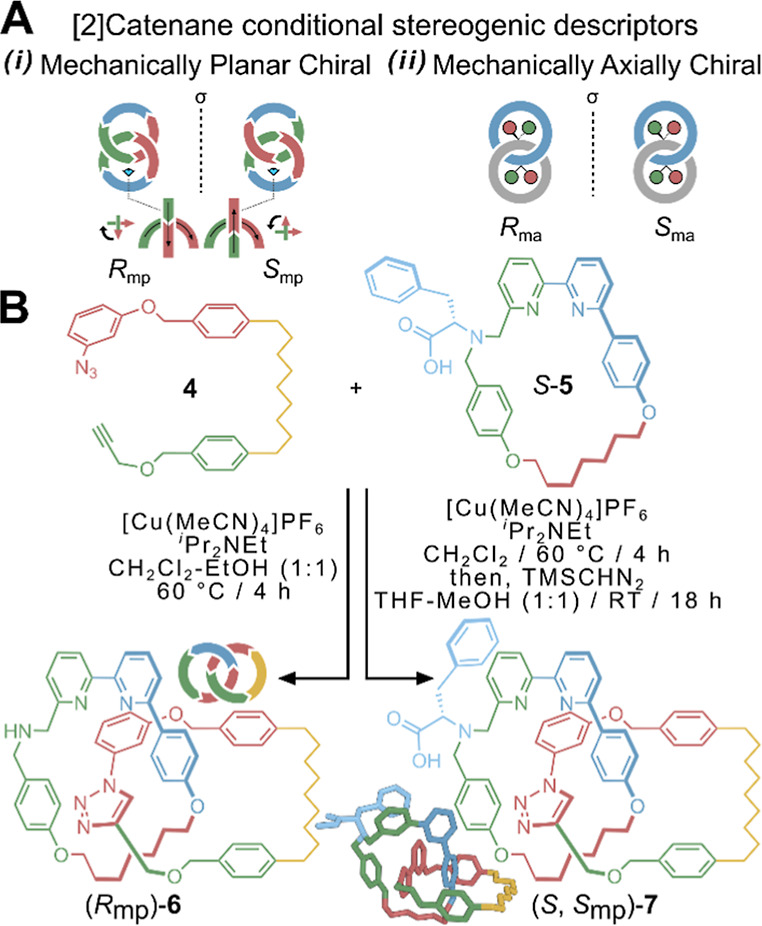
Stereogenic considerations of [2]­catenanes. (A) Hopf links have no unconditional stereogenic elements, but the introduction of oriented rings or facially dissymmetric rings generates conditional stereogenic elements. (i) Two interlocked oriented rings can exist as one of two mechanically planar chiral enantiomers. (ii) Two interlocked facially dissymmetric rings can exist as one of two mechanically axially chiral enantiomers. Red is considered higher priority than green for stereochemical assignment in part A only. (B) Goldup’s synthesis of mechanically planar chiral [2]­catenanes, employing Phe as a chiral auxiliary. The crystal structure (CCDC: 2207369) allowed the assignment of the absolute chirality of (*S*, *S*_mp_)-**7**.

Integration of a small number (≤6) of aas into a short ligand has been shown to generate a myriad of catenane architectures (Figure 11A) upon coordination of a metal ion, with structures characterized by X-ray crystallography.^208–210^ Many Hopf link [2]­catenanes (CN = 2) have been reported ranging in molecular size (7–19 Å diameter macrocycles).^211–216^ Davis and co-workers reported the dipeptide ligand **L3** with terminal pyridylimine binding motifs which chelate Co^2+^ to form a metallopeptide [2]­catenane with two ligands per macrocycle (Figure 11Bi). Four [2]­catenanes were prepared from different enantiomers of the Ala dipeptide unit (*RR*, *SS*, *RS* and *SR*), which all formed the same Hopf link topology, but different folded architectures depending on chiral matching of the aa pair. The [2]­catenanes displayed remarkably high Young’s moduli (up to 157.6 GPa) and minor antimicrobial activity against *E. coli* and *Staphylococcus aureus*.^216^ More complex [2]­catenane topologies have also been reported including a cyclic handcuff [2]­catenane (CN = 4),^217^ Solomon links (CN = 4)^218–220^ and torus knot [2]­catenanes (CN = 8 and 10) with the architectures shown in Figure 11A.^94,95,221^ A remarkably complex architecture comprised of two trefoil knots interlocked into a Hopf link [2]­catenane architecture (CN = 14) formed as a single topologically chiral structure (Figure 11Bii).^222^ Changing the spacer of ligand **L4** from naphthyl (**L4a**) to biphenyl (**L4b**) altered the topology from a trefoil knot to the double trefoil link, with the stereogenic center of Ala in the ligand governing the diastereoselective assembly. Catenanes with increasing numbers of component macrocycles have also been reported,^223,224^ where the macrocycles form the faces of 3D polydehral cages, such as a tetrahedron,^225,226^ cube^227^ or dodecahedron.^228^ A highly entangled structure (CN = 60) reported by Fujita and co-workers is isostructural to the dodecahedral symmetry of the HK97 capsid, but with a different Goldberg triangulation number, *T* = 3 for the metal-peptide capsid and *T* = 7 for the HK97 capsid. A simple ligand with terminal pyridyl binding units and a central Pro-Pro-aminobenzoyl-propargylglycine-Pro unit self-assembled into the Cu_60_L_60_ capsid with a diameter of about 4 nm and an internal volume of ∼34,000 Å^3^. Twelve macrocycles (5 ligands) can be identified in the structure, but due to cross-links between them the architecture is strictly a [1]­catenane. The 60-crossing woven network capsule can also be described as 20 trefoil knots (each formed from 3 ligands) connected via 6 cross-links. By derivatization of the first Pro at the γ-position which was exposed to the surface of the assembly, with di- or tetraethylene glycol methyl ether (DEG or TEG) chains the surface of the dodecahedral capsule could be functionalized. The ease of synthetic preparation of these small peptide-based ligands and the shear complexity of interlocked architectures that can be accessed is astonishing, with their inherent biocompatible and functional utility yet to be unlocked.

**Figure 11:**
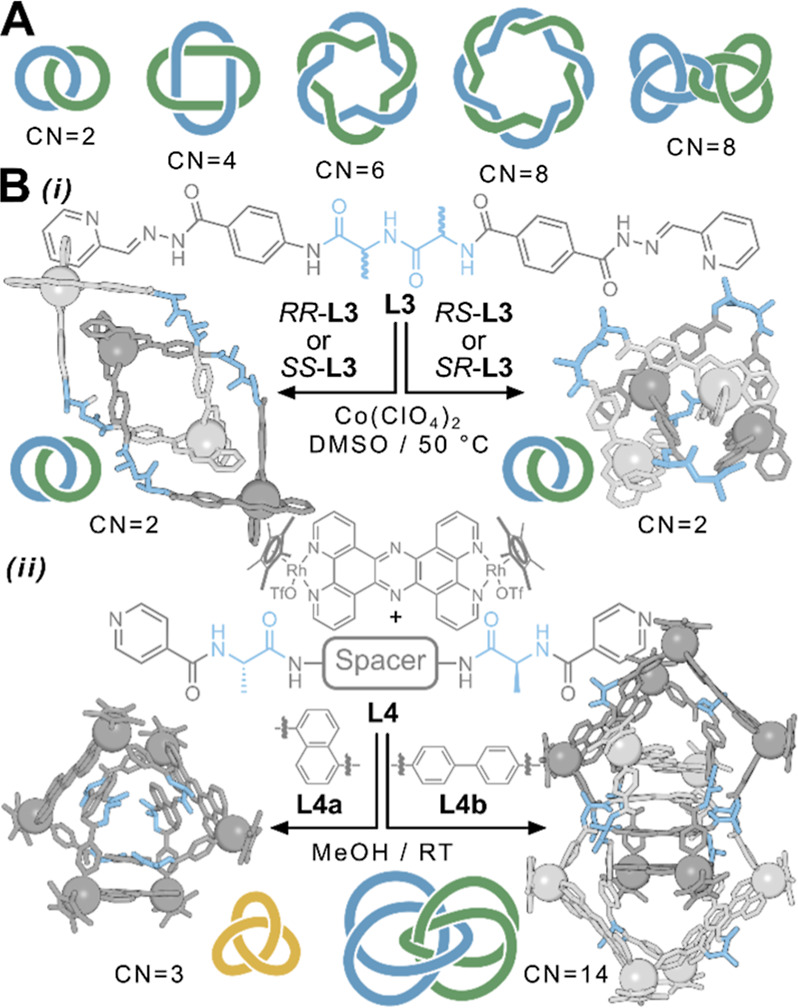
Metal-peptide [2]­catenane complexes with different topologies. (A) Graphics of [2]­catenane topologies including a Hopf link, Solomon link, torus links with CN = 6 and 8, and a trefoil link (from left to right) with CN shown. (B) Metal-peptide complexes with a (i) [2]­catenane topology adopting different conformations (CCDC: 2299892 (left) and 2299893 (right)) as a result of the point chirality of the aa in **L3** and (ii) trefoil knot architecture (CCDC: 2143574) on the left when the ligand spacer is a naphthyl group (**L4a**) or interlocked double trefoil link structure (CCDC: 2143575) on the right when the ligand spacer is a biphenyl group (**L4b**).

## Polypeptide Rotaxanes

### Peptide Rotaxanes Found in Nature

The rotaxane architecture, with a ring threaded onto a linear axle, is prevalent in nature if one considers the structure of many DNA binding proteins (topoisomerases, helicases, DNA and RNA polymerases, and the ribosome).^229^ These essential enzyme complexes are comprised of many polypeptide chains, that generate a supramolecular structure encircling DNA or RNA creating an architecture reminiscent of a rotaxane. Despite the protein assembly not being covalently linked together, the fact that it encircles the nucleic acid chain ensures strong binding and restricts the translational motion of the enzyme complex to a 1-dimensional track. As a result, the genetic code is read without skipping nucleotides, ensuring the high fidelity of DNA replication, transcription and translation of mRNA.

Rotaxane structures have been identified in single proteins, where a single internal cross-link generates a macrocycle in the structure that is pierced by another portion of the protein.^230–233^ These [1]­rotaxane lasso proteins have been identified with up to 6 piercings and are catalogued in the LassoProt and AlphaLasso databases.^234–236^ The threaded architecture of the lasso protein leptin has been shown to influence dynamics of its native state, which impacts biological activity and protein stability, drawing a link between the interlocked structure and biological function.^237^

### Peptide and Protein Cysteine Knots

An interlocked structural motif known as the cysteine knot^238,239^ (previously knottin)^240^ is prevalent in proteins and peptides found in all kingdoms of life.^50,241^ First confirmed by X-ray crystallography in 1982 (PDB: 4CPA),^242^ this motif is comprised of three disulfide bonds forming an interlocked [1]­rotaxane architecture, where two disulfide bonds form a macrocycle via connecting segments of the peptide backbone that is threaded by the third disulfide bond. There are three subclasses of cysteine knot shown in Figure 12A, which differ in the bridge forming residues and the connection of the termini, namely growth factor cysteine knots (GFCK),^243–245^ inhibitor cysteine knots (ICK)^246,247^ and cyclic cysteine knots (CCK).^248^ Confusingly, though, none of these architectures are actually knots, rather they are [1]­rotaxanes with multiple connecting bridges between the chain that threads through the macrocycle. Based on the archetypal structure of a [2]­rotaxane one can add bridges between the macrocycle and one of the ends of the axle; 1 bridge generates a lasso [1]­rotaxane, a bridge at each end of the axle garners a figure-of-eight architecture, 3 bridges can connect from the same end of the axle or from each end as it the case for the GFCK and ICK architectures (Figure 12B). Cyclizing the peptide backbone in the CCK results in a fourth bridge, which gives a topologically complex Möbius topology as a result of the bridging connectivity (Figure 12C).^249^

**Figure 12:**
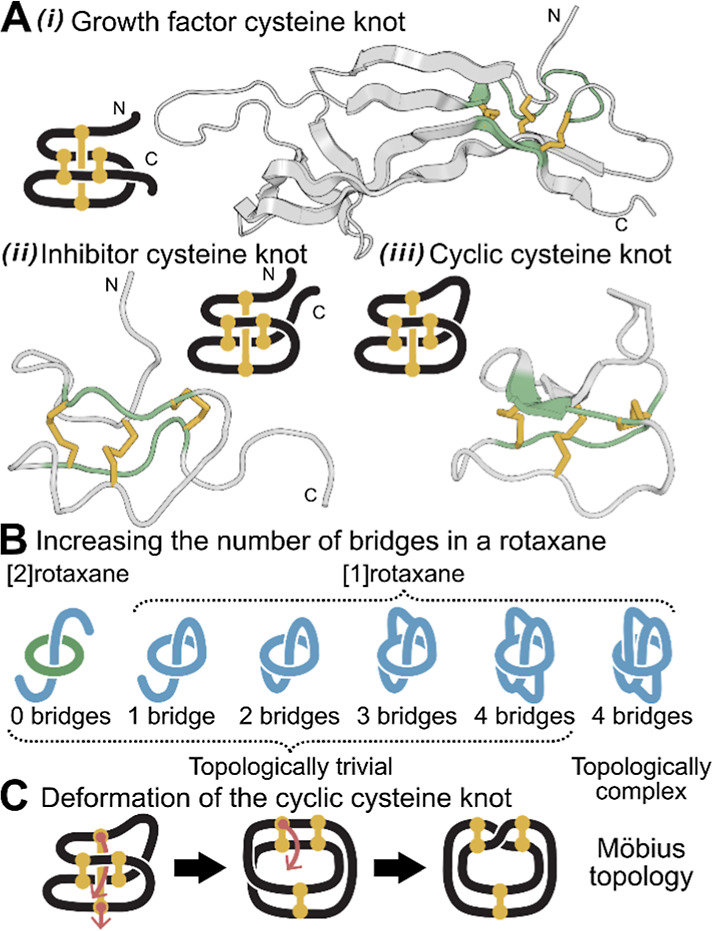
Protein rotaxanes found in nature with a cysteine knot motif. (A) Structure of the cysteine knot containing (i) GFCK *Mus musculus* nerve growth factor protein structure (PDB: 1BET), (ii) ICK *Solanum tuberosum* carboxypeptidase inhibitor (PDB: 4CPA), and (iii) CCK *Oldenlandia affinis* kalata B1 (PDB: 4TTM). Structures are shown as gray ribbons with the disulfide forming cysteine side chains in gold and the residues which form the threaded macrocycle in green. Graphics also show the different connectivities of the three cysteine knot architectures. (B) Graphics showing different rotaxane architectures with an increasing number of connecting bridges between the ring and the strand threaded through it. (C) The connecting pattern of a rotaxane with 4 bridges can generate a topologically complex architecture as is seen for the CCK protein family, which is shown to have Möbius topology by sequential deformation of the architecture.

The prevalence of the cysteine knot motif among a diverse array of biologically active peptides and proteins suggests the interlocked architecture provides unique biological benefits. The first cysteine knot was identified in a carboxypeptidase inhibitor,^242^ which is typical of the ICK miniproteins found in spider, snail, snake and other animal venoms that inhibit proteases, ion channels and other biological targets of pharmacological interest.^239,250,251^ Disulfide bonds confine the protein into a unique structure reducing the degrees of freedom for molecular motion, which is further restricted by the interlocked architecture.^252,253^ The cysteine knot enables stronger and more selective binding, as well as improves properties such as resistance to proteolysis, thermal stability and cell permeability.^254–256^ The cysteine knot motif is the only interlocked architecture characterized from the larger family of disulfide rich proteins (DRP), which can access a vast library of connectivities highlighting the potential for other interlocked architectures to be discovered.

Cyclotides are a class of natural products isolated from plants, belonging to the CCK family.^248,257,258^ They have since been biochemically classified as ribosomally synthesized and post-translationally modified peptides (RiPPs),^259^ encoded in the genome as a precursor peptide, which undergoes oxidative folding, proteolysis of flanking precursor pro-domains, and finally cyclization by an asparaginyl endopeptidase (AEP).^260–262^ These MIP natural products^263–265^ constitute the largest family of cyclic peptides, displaying vast sequence diversity in the loops delineated by the six Cys residues. Cyclotides have been subdivided into two groups depending on the presence of an unusual *cis*-Pro in loop 5 for Möbius cyclotides and no such residue in bracelet cyclotides.^248^ However, as discussed above in fact all cyclotides have Möbius topology irrespective of a *cis*-Pro, and the original report specified that the single chain peptide backbone is strictly speaking not a flat ribbon which is required for such a topology. The Möbius topology of cyclotides is generated by the three disulfide bridges and enzymatic cyclization of the backbone,^260^ but other bioengineered derivatives that cyclize a naturally occurring DRP have garnered a Möbius topology unappreciated in the original reports.^266,267^ The interlocked structure imparts significant thermal stability, resistance to chemical denaturants and proteolytic stability, evidenced by kalata B1, the first cyclotide isolated from the leaves of *Oldenlandia affinis*,^268^ which retains bioactivity when dissolved in boiling water.^269^ Interestingly, the total synthesis of kalata B1 confirmed that the native disulfide linkages spontaneously form in water upon oxidation of the cyclic peptide and a linear derivative was also found to form the same interlocked architecture.^270^

Owing to the enhanced stability and selective binding conferred by the cysteine knot motif, an array of bioengineering studies have employed this mechanically interlocked architecture for biotechnological applications.^271–276^ Replacing a loop sequence for a binding epitope has enabled the rational design of cysteine knots that bind new biological targets, with the beneficial stability and membrane permeability of the interlocked scaffold.^277–280^ Bioconjugation of fluorescent probes and drugs to a cysteine knot has facilitated localization of the appended moiety to the native biological target, where the stability and selective binding afforded by the cysteine knot is akin to antibody scaffolds.^281–284^ The application of biotechnologies to screen vast genetically encoded libraries of mutants of a cysteine knot has generated variants with new binding selectivities and affinities in the picomolar range.^285–293^ Overall, this has culminated in the approval of two cysteine knot-based drugs, linaclotide, an orally available treatment for irritable bowel syndrome, and ziconotide, an intrathecally administered analgesic agent for the amelioration of severe and chronic pain, epitomizing the benefit of interlocked therapeutics.^294^

## Lasso Peptides

Polypeptide chains have been identified in nature in the form of small [1]­rotaxane natural products referred to as lasso peptides.^295–298^ The interlocked structure was first confirmed by nuclear Overhauser effect (NOE) correlations from solution state 2D ^1^H NMR experiments for natural products isolated from *Streptomyces* bacteria as early as 1994 (PDB: 1RPB),^299–301^ with the term ‘lasso’ being coined for this structural motif in 1996.^302^ However, it was not until 2003 when three groups independently assigned the structure of microcin J25 (MccJ25) (PDB: 1Q71, 1PP5) by analogous 2D ^1^H NMR experiments that this structural feature became widely known.^303–305^ MccJ25 was identified in 1992 from an antimicrobial activity screen of isolates obtained from the gut microbiota of a human newborn.^306^ Through screening isolates from the gut microbiome against *E. coli* marker strains separated by a 10 kDa molecular weight cut off cellophane sheet, it was hoped antibiotics with a low molecular weight and high stability to gut proteases would be identified. MccJ25 was purified from the supernatant of the *E. coli* AY25 liquid cultures. Remarkably, purified MccJ25 retained its interlocked structure and antimicrobial activity despite boiling temperatures (100 °C, 30 min) and even autoclaving (120 °C, 15 min).

Microcin J25 and the MIPs from *Streptomyces*, have since been realized to be produced following the same biosynthetic logic and as such are all referred to as lasso peptides. The lasso peptides are themselves a class of RiPPs,^259^ which are natural products encoded in the genome as a precursor peptide, that is produced by the ribosome and then post-translationally modified (PTM) into the final mature RiPP. Tailoring enzymes, which are typically encoded in genes proximal to the precursor in the biosynthetic gene cluster (BGC), selectively decorate the precursor peptide guided by supramolecular recognition of the N-terminal leader peptide (leader-dependent enzymes) or the C-terminal core (leader-independent enzymes). The gene encoded nature of the lasso peptides and the growing list of whole genome sequences acquired, has enabled computationally guided genome mining to identify many thousands of putative lasso peptides throughout the bacterial domain.^307–309^ Indeed, many open source programs have been developed which parse genomic data in search of RiPP natural product BGCs including RODEO,^308^ BAGEL,^310^ and antiSMASH.^311^

Lasso peptide BGCs typically contain at least three genes necessary for maturation of the interlocked product: the precursor gene (A), a leader peptidase gene (B) and a lasso macrocyclase gene (C) (Figure 13A). Lasso precursor peptides generally have short (20–40 aa) N-terminal leader peptides with a C-terminal core peptide (13–33 aa).^312,313^ The peptidase gene may be split in two, generating two protein domains (a RiPP recognition element^314,315^ which guides recognition of the precursor peptide and the protease enzyme domain necessary for proteolytic cleavage) which assemble to form the active enzyme.^316–318^ The precursor is recognized by the peptidase which cleaves the leader sequence before the core is processed by the macrocyclase.^319–321^ Within the active site of the macrocylase the core sequence is preorganized such that adenylation of an Asp of Glu side chain carboxylate and subsequent nucleophilic attack by the peptide N-terminus forms an isopeptide bond trapping the molecule into an interlocked architecture (Figure 13B).^322,323^ It has been proposed that the peptidase (B) and macrocyclase (C) must form a complex for the two enzymes to become catalytically active^320^ and demonstrated by size exclusion chromatography that these enzymes indeed form a complex.^324^ The [1]­rotaxane structure, reminiscent of a cable tie, for all lasso peptides has been found to have the same right-handed structural chirality, formally denoted *S*_mp_ following Goldup’s assignment rules for mechanically planar chirality.^197^ Lasso peptides have been structurally characterized with 7,^312,325^ 8 ^306,313,326^ and 9 ^327^ aa rings. The linear portion of the lasso peptide consists of the loop region (residues after the macrocycle but not threaded through) and the tail region (C-terminal residues threaded through the macrocycle) and methods exist for the computational prediction of the interlocked structure of any lasso peptide precursor.^328,329^

**Figure 13:**
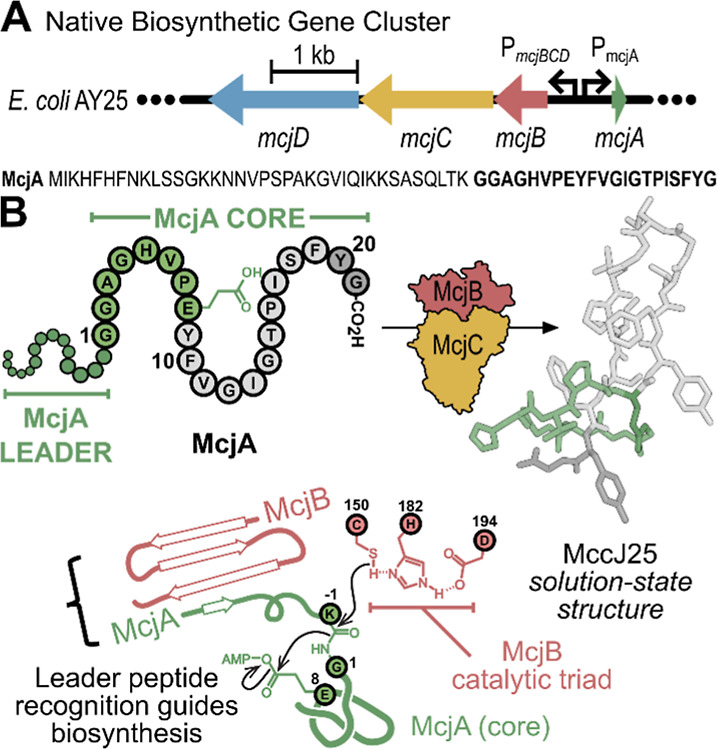
Biosynthesis of the [1]­rotaxane lasso peptide MccJ25. (A) The native biosynthetic gene cluster of MccJ25 with genes for the precursor peptide (*mcjA*, green), the lasso peptidase (*mcjB*, red), the lasso cyclase (*mcjC*, gold) and ABC transporter (*mcjD*, blue). The sequence of the 58 aa precursor peptide is shown, with the core sequence in bold. (B) The biosynthesis of MccJ25 proceeds via excision of the leader peptide and subsequent nucleophilic attack of an adenylated side chain residue to form an isopeptide bond which kinetically traps the tail into an interlocked architecture. The structure of the lasso peptide MccJ25 (PDB: 1Q71) is colored to highlight the ring (green), loop (light gray) and tail (dark gray) regions.

A dedicated membrane transporter is also commonly found colocalized within the lasso peptide BGC. For MccJ25, the gene *mcjD* encodes an ATP-binding cassette (ABC) transporter McjD.^330,331^ Given the antimicrobial activity of MccJ25, a means of protecting the producing cell from the cytotoxic compound is required. The transporter McjD serves to actively transport MccJ25 out of the cytoplasm across the inner membrane serving as an immunity factor, ensuring a low intracellular concentration of MccJ25. The native outer membrane protein channel TolC, which has a 140 Å pore length, has been implicated in the export of MccJ25 facilitating the direct transport of the lasso peptide from the intracellular environment through McjD across the inner membrane and then through TolC, bypassing the periplasm and outer membrane.^332^ Transient opening of the McjD transporter pore prevents cytoxic MccJ25 from reentry to the cell thus serving as a self-immunity factor to the cell.^333^

The presence of Cys residues leads to the formation of lasso peptides with additional dynamic covalent disulfide cross-links, though the mechanism governing disulfide bond formation in these peptides remains unknown.^334^ The structures are categorized into classes defined by the number of disulfides (Figure 14). Class I lasso peptides^299,300^ were the first characterized lasso peptides, comprising of 2 disulfide bonds (one tail-ring, one ring-loop) that form a tricyclic lasso architecture that is topologically trivial. Class II lasso peptides^306^ are by far the largest class, comprising no disulfide bonds, with a [1]­rotaxane architecture typically stabilized by bulky aa side chains on the threaded portion of the peptide which sterically block the macrocycle from unthreading. Class III lasso peptides^335,336^ have one disulfide bond (tail-ring) generating a molecular figure-of-eight structure. Class IV lasso peptides^308,313^ also have one disulfide bond (tail-tail) forming a ring within the tail. Achromonodin-1 ^337^ (a Class II lasso peptide) holds the record for the largest loop at 20 aa (30 aa total), while pandonodin^313^ (a Class IV lasso peptide) has the longest tail at 18 aa (33 aa total). The disulfide bonds likely help stabilize the lasso, however, are not required to maintain the interlocked architecture. The interlocked structure of lasso peptides is typically determined by NOE correlations in 2D ^1^H NMR experiments,^338^ along with tandem mass spectrometry (MS^n^)^339,340^ and IMS-MS,^341,342^ and has been determined by X-ray crystallography in some cases.^325,343,344^ The structure of lasso peptides binding target proteins have also been determined by X-ray crystallography^345–349^ and cryo-electron microscopy.^350^

**Figure 14:**
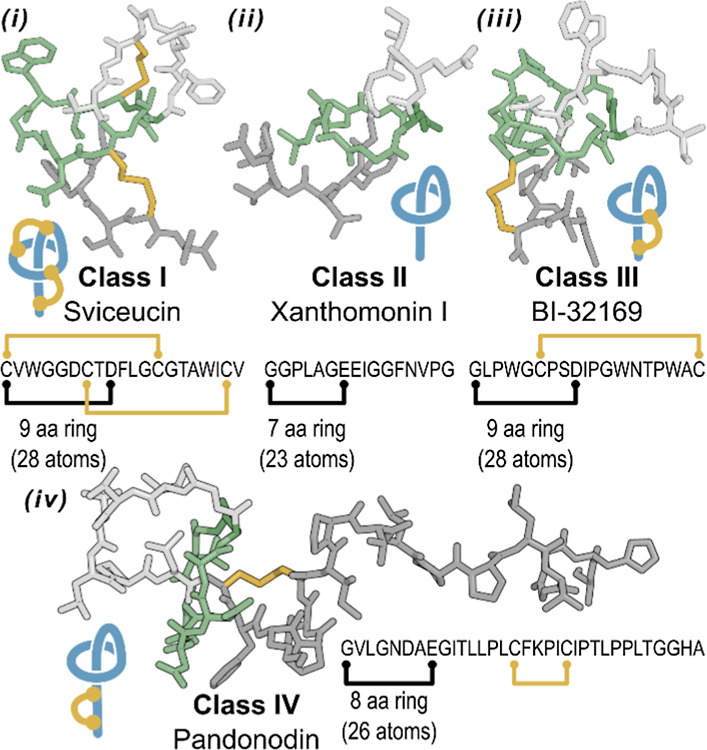
Structural diversity of lasso peptides. Depending on the presence of disulfide bridges, lasso peptides are classified into (i) Class I, with two disulfide bridges (ring-loop and ring-tail) such as sviceucin (solution structure PDB: 2LS1), (ii) Class II, with no disulfide bridges such as xanthomonin I (solid-state structure lacking 4 tail aa, PDB: 4NAG), (iii) Class III, with one disulfide bridge (ring-tail) such as BI-32169 (solid-state structure, PDB: 3NJW), and (iv) Class IV, with one disulfide bridge (tail-tail) such as pandonodin (solution structure, PDB: 6Q1X).

Beyond the class defining formation of the macrolactam ring around the tail, lasso peptides have been discovered with an array of other PTMs.^351^ Traditionally, natural product discovery has been guided by phenotypic screening, cultivating an array of bacterial strains and evaluating the cell extracts for bioactivity. This phenotype driven approach has resulted in the discovery of lasso peptides with C-terminal methylation (lassomycin),^352,353^ hydroxylation (RES-701),^354,355^ aa epimerization (MS-271),^356–360^ and most recently formation of a second isopeptide bond resulting in a figure-of-eight structure (lariocidin).^348^ Elicitor screening has also been shown to be an effective method for the discovery of lasso peptides with additional PTMs and unconventional BGCs.^361–363^ Lasso peptides harboring further chemical transformations have also been discovered via genome mining followed by metabolomics or proteomics on the native host.^298,307,308^ Citrulassin, harboring a citrulline residue derived from Arg,^308,364^ was isolated in this way as were lasso peptides with aromatic side chain cross-links,^365^ Trp chlorination,^366–368^ Trp prenylation,^349,366^ Ser glycosylation^369^ and examples harboring multiple unique PTMs.^370–372^ Genome mining can also be followed by heterologous reconstitution of the lasso peptide BGC in a more convenient host, such as *E. coli* or *Streptomyces*. The lasso peptide paeninodin, phosphorylated on its C-terminal Ser hydroxyl, was discovered via heterologous expression,^373,374^ as was the Lys-acetylated lasso peptide albusnodin^375^ and the Tyr-acylated lasso peptide Snou-LP.^376^ Heterologous expression of the antimicrobial lasso peptide triculamin^377^ revealed that Lys acetylation in these molecules serves as a self-resistance mechanism,^378^ which has also been demonstrated for lariocidin.^379^

In many of the cases discussed above, the additional PTMs are in fact installed on the linear precursor peptide (A protein), prior to the formation of the lasso peptide.^367,373^ Recently our group has reported lasso peptides lihuanodin and cellulonodin-2 that are decorated with a single aspartimide moiety within their rings.^380^ This modification comes about via enzymatic methylation of a specific aspartate residue in the lasso peptide ring followed by spontaneous dehydration, concomitant with elimination of methanol.^381^ In contrast to the cases discussed above, this PTM only occurs on the interlocked substrate; neither the linear sequence nor a cyclic peptide corresponding to the lasso peptide ring is a substrate for the methyltransferase. Thus, the supramolecular [1]­rotaxane structure of these lasso peptides is the key recognition element for enzymatic modification. Prenylation,^349^ acetylation,^375,378^ aspartimidylation^380^ and cross-linking the C-terminus with the ring^348^ are the only PTMs that have been confirmed to be installed after formation of the interlocked architecture. Another example of an enzyme requiring the [1]­rotaxane structure of the lasso peptide for molecular recognition is the lasso peptide isopeptidase.^317^ This enzyme, the gene for which is found colocalized with lasso peptide BGCs primarily in α-proteobacteria, recognizes only threaded lasso peptides and cleaves the isopeptide bond, returning the lasso peptide to a linear peptide.^317,344,382^ A cocrystal structure of the lasso peptide isopeptidase from *Asticcacaulis excentricus* with its substrate, the lasso peptide astexin-3 shows a large cavity within the active site that readily accommodates the unique rotaxane structure of the lasso peptide.^346^

Based on the historical bioactivity screening methods employed for natural product discovery, many antimicrobial lasso peptides have been identified, serving as a fitness advantage to the producing cell.^295,297^ The antimicrobial activity of lasso peptides is typically highly strain specific, with cytotoxicity targeted against closely related strains, or bacterial species in competition with the producer strain in its natural environment.^383^ This specificity is the result of the transporter-dependent uptake mechanism of lasso peptides with an intracellular target. For example, MccJ25 displays antimicrobial activity against Gram-negative bacterial species of *Escherichia*, *Salmonella*, and *Shigella*.^306^ MccJ25 hijacks the iron–siderophore transporter FhuA with the rotaxane structure serving as a reasonable facsimile to the native substrate of FhuA, ferrichrome.^347,384–386^ The intracellular target of MccJ25 was implicated to be the RNA polymerase (RNAP), when DNA sequencing of a resistant *E. coli* mutant revealed a single point mutation in the *rpoC* gene which introduces a T931I-substitution in the RNAP β′ subunit.^387^ Studying the activity of purified *E. coli* RNAP in vitro confirmed that MccJ25 inhibited transcription^388^ and the noninterlocked structure was found to not inhibit enzyme activity.^303^ The crystal structure of MccJ25 bound to *E. coli* RNAP has been solved, showing how the rotaxane structure is able to engage the secondary channel of RNAP and act as an inhibitor.^345^ A number of lasso peptides have been identified which all exhibit unique species specific antimicrobial activity, through inhibition of RNAP, including capistruin,^389^ acinetodin,^383^ klebsidin,^383^ citrocin,^390^ ubonodin,^326^ the achromonodins,^337^ MccY^391^ and cloacaenodin.^392^ The number of RNAP-inhibiting lasso peptides emphasize the sequence and structural diversity with different ring, loop and tail lengths all capable of binding RNAP, but leads to alternative transporter binding specificities which influences the strain susceptibility to the antimicrobial lasso peptide.^393,394^

Lasso peptides have been identified with alternative biological targets, which display antimicrobial activity. For example, an antimycobacterial activity screen searching for treatments of tuberculosis identified triculamin in 1958,^395,396^ which was not structurally characterized as a lasso peptide until 2022.^377^ Lariatin,^397^ lassomycin,^352^ lariocidin^348^ and gelatinamin^378^ all display antimycobacterial activity.^398^ Lassomycin was shown to bind the ClpC1 ATPase complex and act as an agonist overstimulating ATPase activity and uncoupling it from its native protease activity with the ClpP1P2 complex leading to cell death.^352^ Interestingly, the recently discovered lasso peptide lariocidin displays broad-spectrum antimicrobial activity, due to his high positive charge (+7 charge in a 17 aa peptide) facilitating a transporter-independent membrane translocation mechanism as well as its inhibition of the ribosome. The uniquely compact lasso structure allows for both cell-penetrating and ribosome-inhibiting activities to be packaged within a single molecule.^348^

### Lasso Peptide Dynamics

The [1]­rotaxane structure of lasso peptides is generally highly stable, but in some cases unthreading is observed at elevated temperatures.^399^ MccJ25 is extremely stable, where heating (100 °C, 30 min) does not alter the interlocked structure leading to retention of antimicrobial activity.^306^ The caulosegnin family of lasso peptides were the first shown to thermally unthreaded.^400^ Three precursor peptides are encoded within the BGC from *Caulobacter segnis*, and are all processed by the same biosynthetic enzymes yielding the lasso peptides (caulosegnins I, II, and III) with 8 and 9 aa rings. Heating each of the caulosegnins (95 °C, 4 h) and analyzing the product distribution by HPLC revealed different thermal stabilities. Caulosegnin I, despite having the smallest ring size (8 aa), was completely converted into a new species, that was now susceptible to carboxypeptidase degradation suggesting a loss of the interlocked structure. Caulosegnin II (9 aa ring) was stable to heat treatment, while caulosegnin III (9 aa ring) was hydrolyzed upon heating. Although caulosegnin I has the smallest ring size, it is the least thermally stable, highlighting that the residues flanking the ring likely have a greater impact on thermal stability of the lasso peptides. Thermodynamic and kinetic parameters of astexin-2 and astexin-3 variant unthreading were determined and supported by molecular dynamics modeling, which probed the influence of residue size, hydrophobicity, and polarity on the free energy barrier to unthreading.^401^ ^1^H NMR spectroscopy has proven to be an effective tool at elucidating the three-dimensional structure of lasso peptides,^338^ revealing interlocked peptides have a wide chemical shift dispersion for amide protons (typically between 6 and 10 ppm), indicating a well-folded structure, while the unthreaded conformer has a narrower dispersion of amide protons.^402,403^

The lasso peptide benenodin-1 was shown to display shuttling motion between threaded coconformers at elevated temperatures (Figure 15).^404^ Benenodin-1 is a lasso peptide from the α-proteobacterium *Asticcacaulis benevestitus*, produced as a [1]­rotaxane with an 8 aa ring threaded onto the linear chain and held between the flanking residues Glu14 and Gln15. This coconformational isomer was shown to thermally convert to another, with Lys17 acting as a stopper (loop pulling). The rate of shuttling at room temperature is slow, allowing the two coconformational isomers to be isolated and the 3D structure of each to be determined by ^1^H NMR spectroscopy. A further study identified another two minor coconformational isomers of benenodin-1 in thermal exchange, with the ring further from the C-termini (tail pulling, between Gln13 and Glu14 or Thr12 and Gln13) than the naturally produced isomer.^405^ Computational modeling probed the balance between putative mechanical strain and noncovalent interactions that govern the equilibrium distribution of the coconformational isomers.^406^

**Figure 15:**
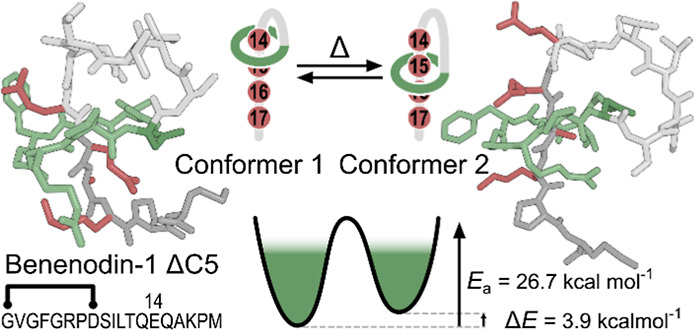
Shuttling motion in a naturally occurring lasso peptide. Benenodin-1 (8 aa/25 atom ring) is isolated with 5 aa truncated from the tail as conformer 1 with the ring between E14-Q15 (PDB 5TJ1). Upon heating the lasso peptide an equilibrium of coconformational isomers is reached, with the structure of conformer 2 (PDB: 6B5W) with the ring straddling Ala16 determined by ^1^H NMR.

### Engineering Lasso Peptides in Cellulo

Since the lasso peptide precursor is gene-encoded, recombinant DNA tools can be employed to generate variants of lasso peptides. Coexpression of these new precursors with the lasso peptide maturation enzymes in cellulo allows alterations of different lasso peptide scaffolds to be evaluated,^407^ enabling the function of new lasso peptide sequences to be evaluated. Single substitutions are typically well tolerated, other than at the acidic isopeptide forming residue, but more ambitious precursor engineering such as additions or deletions within the core peptide are generally poorly tolerated. Alteration of the ring size by one residue has been shown to significantly reduce lasso peptide production,^407,408^ suggesting the selectivity of the tailoring enzymes for recognizing a specific substrate length. Reducing the loop size of MccJ25 and caulosegnin I has been shown to be moderately tolerated, but no examples of increasing the loop size of a lasso peptide have been reported.^400,408^ Despite the macrocycle typically being less amenable to alteration, expansion and contraction of the fusilassin (also called fuscanodin)^324^ macrocycle (7–10 aa, natively 9 aa) was tolerated^409^ and the caulosegnin maturation enzymes natively produce 8 and 9 aa macrocycle lasso peptides.^400^

The steric lock residues of most lasso peptides tend to have bulky aromatic side chains, which restrict the motion of the macrocycle. Substituting the lock residues tends to prevent maturation of any product or in some cases has been shown to generate a pseudo[1]­rotaxane which is liable to thermal unthreading.^400,401,407,408,410^ Substitutions within the macrolactam ring influence the thermal stability of the lasso to unthreading, where substitutions that introduce more flexibility tend to reduce the barrier to unthreading.^411^ An interesting variant of lihuanodin harboring a T7L substitution in the macrolactam ring displayed controllable unthreading in response to enzymatic PTM (Figure 16).^381^ Lihuanodin^380^ is a lasso peptide produced by the firmicute *Lihuaxuella thermophila* with a 9 aa (28 atom ring) containing a stable aspartimide introduced by the methyltransferase LihM. The lihuanodin aspartimide is remarkably stable, however upon hydrolysis preferential regioselective ring opening is observed regenerating the canonical Asp residue. Substitution of Thr7, which likely promotes regioselective hydrolysis to Leu results in a 3:1 preference for the isoAsp hydrolysis product increasing the macrocycle ring by 1 atom (9 aa, 29 atom ring) resulting in the spontaneous unthreading of the peptide (Figure 16).^381^

**Figure 16:**
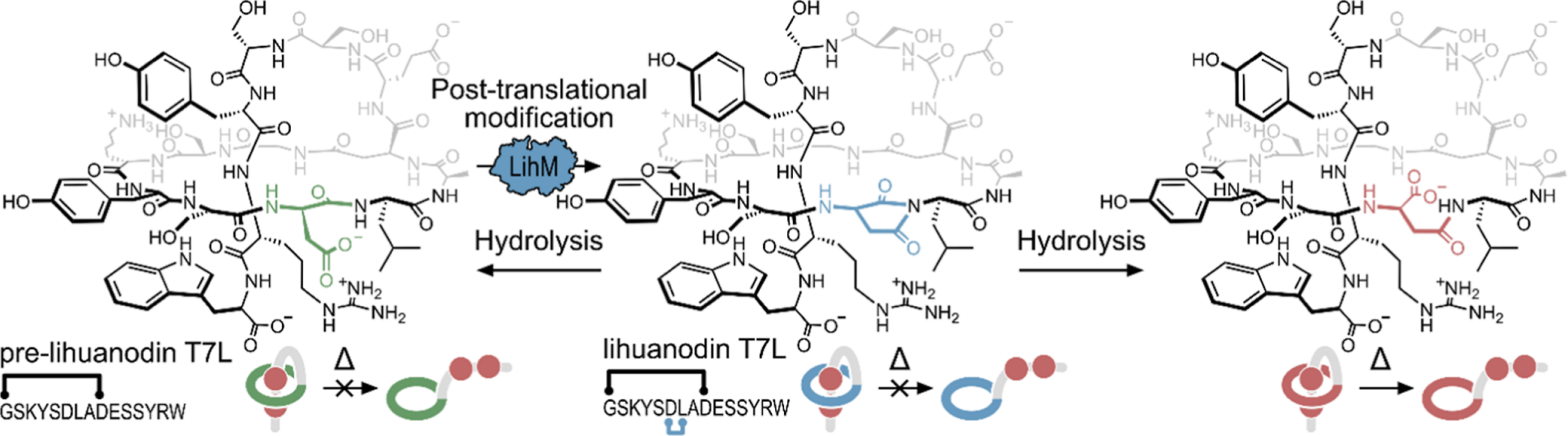
Engineering lasso peptides to promote nanomechanical motion. Lihuanodin is a lasso peptide (9 aa, 28 atom ring) with an enzymatically installed aspartimidylation (blue) in the ring, which regioselectively hydrolyses to regenerate the canonical Asp residue (green). Introduction of T7L substitution in lihuanodin favors hydrolysis to the isoAsp (red) product increasing the macrocycle ring by 1 atom (9 aa, 29 atom ring) resulting in the spontaneous unthreading of the peptide.

Tail deletions can be tolerated up to the residue below the bottom steric lock, as would be expected based on the observation of these truncated products, cleavage by endogenous carboxypeptidases during cellular expression.^313,400,408^ The addition of amino acids to the tail of the precursor peptide typically results in a significant drop in product formation,^407,408^ however has been shown to be well tolerated for astexin-1,^412^ pandonodin^313^ and other lasso peptides^413,414^ allowing [1]­rotaxane architectures to be matured with a fusion protein appended to the C-termini. The introduction of noncanonical amino acids into the lasso peptide scaffold has been achieved through the amber suppression protocol.^345,415,416^ Four *meta*-substituted Phe derivatives (chloro, bromo, nitro and trifluoromethyl) were each introduced into one of four positions within MccJ25 (V6 in the loop, Phe10 and Ile13 in the loop and steric lock Phe19) site-specifically using the pyrrolysyl-tRNA synthetase (PylRS) system (Figure 17A).^415^ MccJ25 variants with *para*-bromo-Phe substituted at positions H5 and F10 generated following an equivalent protocol were used to obtain cocrystals bound to RNAP, with the heavy bromine atoms enabling unambiguous placement and refinement of an MccJ25 atomic model into the X-ray crystal structure density.^345^

**Figure 17:**
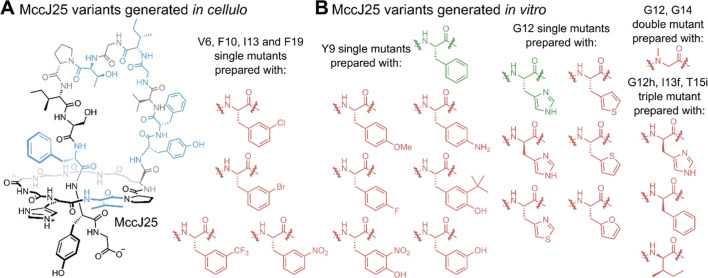
Incorporation of noncanonical amino acids into MccJ25. The structure of MccJ25 is shown with the positions tolerant to substitution colored blue, with the structure of the noncanonical amino acids incorporated (A) in cellulo and (B) in vitro colored red. Canonical amino acids substituted into the structure of MccJ25 in vitro are colored green.

Engineering efforts directed at altering the tailoring enzymes of lasso peptide products are still in their infancy, with studies mainly guided by gaining understanding of the biosynthetic logic.^316,323,417,418^ However, recently the rational design of lasso cyclase mutants has been reported to expand their substrate scope enabling maturation of previously inaccessible lasso peptide variants.^322^ Despite there being no crystal structures of lasso cyclases, models could be generated with AlphaFold, ESMFold and OmegaFold each giving nearly identical structures. Molecular dynamics simulations of the cyclase with the adenylated core peptide were then conducted, revealing key interacting residues for subsequent mutational analysis. The engineered MccJ25 cyclase (McjC) bearing K252A and K388A substitutions was used in a subsequent study to great effect, significantly increasing the yield of MccJ25 variants harboring up to 9 substitutions and 1 deletion within the core peptide.^419^ Combining enzymes from other biosynthetic pathways with the lasso peptide biosynthetic enzymes in cellulo is a new strategy developed to expand the chemical diversity of lasso peptides.^420^ In this area, our group has recently shown that the graspetide synthetase, ThfB, can be encoded in a BGC to introduce an ester-cross-linked macrocycle into the tail of an engineered pandonodin variant in cellulo.^421^

As well as providing insights into the mechanism of biosynthesis, variants have been generated to evaluate the structure–activity relationship of biologically active lasso peptides.^392,422^ The antimicrobial activity of lasso peptides has been utilized as an observable output to screen variants in cellulo in high-throughput.^423–425^ Typically antimicrobial lasso peptides have a transporter gene in the BGC, to provide immunity to the host organism. By controlling the expression of the precursor peptide and the transporter under orthogonally inducible promotors, the biosynthesis of the lasso peptide can be uncoupled from the transporter. In the scenario of all genes being expressed the antimicrobial lasso peptide will be produced and exported from the cell, however, when expression of the transporter gene is turned off the intracellular accumulation of the lasso peptide will kill the host cell. Transforming cells with saturation mutagenesis libraries of the precursor peptide and comparing the growth of colonies with and without the transporter through replica plating or next generation sequencing allows the identification of antimicrobial variants. Screens have been conducted in this way for the evaluation of production and antimicrobial activity of variants with aa substitutions of MccJ25,^423^ klebsidin,^424^ and ubonodin.^425^ A library of MccJ25 variants has also been screened for activity against *Salmonella* and *E. coli* strains to gain structural insights into strain specific bioactivity.^426^ From these screens detailed structure–activity relationships can be obtained allowing deep learning models to be generated such as DeepLasso, which accurately predicts the activity of an ubonodin variant based solely on its sequence.

Beyond evaluation of biological function (antimicrobial activity), lasso peptides have been engineered to have new biological activities.^427^ Hegemann et al. first demonstrated that lasso peptides could be used as a scaffold for epitope grafting, where a small peptide sequence was introduced into the precursor peptide to generate interlocked lasso peptide variants with new biological activities.^428^ Introducing the tripeptide integrin binding motif RGD into the loop of MccJ25 (positions 12–14) gave a threaded lasso peptide that served as a potent integrin antagonist. Subsequent variants have been generated, with further substitutions, that tune the binding affinity and selectivity for αvβ6/8 integrins for the development of anticancer drugs.^419,429^ MccJ25 variants have been engineered to bind other biological targets including the Clp-ATPase for potential antimicrobial activity,^430^ and collagen to serve as a potential antithrombosis agent.^431^

### Lasso Peptide Synthesis In Vitro

Early examples of reconstituting the biosynthesis of MccJ25 in vitro were hindered by low expression yield, insolubility and instability (prone to aggregation) of the tailoring enzymes.^320,432,433^ However, experiments did reveal a strict interdependence of the two enzymes (McjB and McjC) which were only active in the presence of the other protein, even a functionally inactive mutant. For systems with a split protease (B1/B2) the purification of the RiPP recognition element (RRE) and protease proteins has allowed the measurement of binding interactions^314,434^ and reconstitution of protease activity^316,370,418,435,436^ with the precursor peptide. The structural basis for recognition of specific residues within the leader by the RRE (B1) has been confirmed by X-ray crystallography.^319,321^

The Link and Mitchell groups simultaneously reported the complete in vitro reconstitution of the same lasso peptide (named fuscanodin^324^ and fusilassin^409^ by respective groups) produced by the thermophilic actinobacterium *Thermobifida fusca*. Both reasoned that searching for lasso peptide gene clusters in thermophilic bacteria, would yield enzymes that are more stable than those from bacteria found at lower temperatures. The lasso peptide BGC contained four putative genes (*A*, *B1*, *B2* and *C*) which were each heterologously expressed in *E. coli* with an N-terminal His_6_ or MBP for purification. Coexpression and purification of the His_6_ tagged B2 and C proteins as a complex was necessary to isolate soluble proteins,^324^ while the MBP tag enabled purification of the proteins individually.^409^ The minimal biosynthetic components were confirmed in vitro, with all four proteins, ATP and Mg^2+^ being essential. Interestingly, a precursor without the leader peptide was still processed albeit at a reduced efficiency, but no reaction was observed in the absence of B1, revealing the requirement of this protein for catalytic competency of the lasso peptide synthetase. Mitchell and co-workers built on this work to implement fuscanodin/fusilassin biosynthesis using cell-free biosynthesis (CFB).^322,437–439^ The use of CFB coupled with mass spectrometry has allowed screening of libraries of fusilassin variants, to evaluate the efficiency of lasso peptide maturation. Substitutions in MccJ25, ubonodin, and klebsidin are preferentially tolerated in the loop, while fusilassin, in contrast, tolerated substitutions in the ring region. The tailored language model LassoESM was developed using this data set as a method to predict substrate compatibility of a lasso peptide precursor and a lasso synthetase enzyme.^440^ CFB has also enabled the high-throughput screening of precusor/RRE interactions from uncharacterized lasso peptide BGCs,^441^ however of the 42 precursor/RRE pairs, only 27 were found to bind in the assay and just one lasso peptide could be successfully matured in vitro (Las24, also named Las-1010).^414^

The Hartrampf group have recently demonstrated that merging automated fast-flow peptide synthesis (AFPS) for the preparation of the precursor (McjA, 57 aa) with enzymatic maturation in vitro enabled the production of MccJ25 variants containing multiple noncanonical amino acids and core peptide substitutions (Figure 17B).^442^ To improve expression yields of the maturation enzymes (McjB and McjC), the genes were codon optimized and expressed in various *E. coli* strains, which identified C43­(DE3) as the superior host. His_6_-MBP-McjB and His_6_-McjC were expressed and purified separately and used in vitro to prepare MccJ25 variants with substitutions in the loop region. Seven different benzylic noncanonical amino acids could be substituted at position Tyr9, while 6 different 5-membered heterocycles were tolerated in place of Gly12. Up to three positions (Gly12, Ile13,Thr15) could be substituted with d-amino acids within the loop and up to two backbone *N*-methylations (G12, G14) were well tolerated. All variants were confirmed to have an interlocked structure by LC-IMS-MS, which results in a lower collision cross section (CCS) value in ion mobility measurements for the more compact threaded conformation compared to the unthreaded conformer.

Beyond systems attempting to expand the sequence diversity of lasso peptides produced in vitro, a number of enzymatic and chemical modifications of purified lasso peptides have been reported. Characteristically, the interlocked structure of a lasso peptide imparts stability to proteases, with resistance to carboxypeptidase typically being used as evidence for the threading of the linear portion of the peptide through the macrolactam ring.^400^ While MccJ25 was stable to chymotrypsin and pepsin, incubation with thermolysin (cleaves amide bond next to hydrophobic residues) cleaves the peptide backbone between Phe10 and Val11.^443^ The cleaved product was remarkably stable to unthreading^444^ (65 °C/8 M urea/16 h) allowing the 3D structure of the [2]­rotaxane to be determined in CD_3_OH by ^1^H NMR spectroscopy (PDB: 1S7P).^445^ The loop of MccJ25 can be chemically cleaved removing residues T15-I17 or I13–I17 upon incubation in acidic solution (12 M HCl/37 °C/10 h) to give thermally stable [2]­rotaxanes.^445^ The cleaved MccJ25 [2]­rotaxanes all displayed reduced antimicrobial activity, despite equivalent inhibition of RNAP in vitro, suggesting inhibition of cellular uptake by membrane transporters.^446,447^ A T12R variant of benenodin-1 retained the thermal switching between two coconformers, which interestingly displayed different rates of proteolysis when incubated with trypsin (cleavage after K or R).^404^ The conformer with the macrocycle closer to the Arg residue displayed enhanced proteolytic stability, but both conformers could be cleaved with trypsin to generate a peptide [2]­rotaxane. A chemical methodology to promote amide hydrolysis in peptides by cyclizing Ser, Thr, Cys or Glu side chains with a backbone nitrogen increasing the nucleophilicity of the backbone carbonyl was used to generate a benenodin [2]­rotaxane.^448^

Chemical modification of purified lasso peptides has expanded their chemical diversity. Amide coupling reagents have been used to amidate the C-terminus of MccJ25 to probe antimicrobial activity,^449–451^ as well as introduce moieties to improve biological targeting^452^ and stability^419^ of conjugates for anticancer applications. Introducing aa substitutions in lasso peptides can provide new functional handles for coupling reactions, with Lys introduced to provide new sites for amide coupling,^419^ Cys introduced for thiol-maleimide chemistry^401^ and noncanonical amino acids bearing a terminal alkene being used for alkene metathesis.^416^ The lasso peptide stlassin was used as a platform to demonstrate a range of chemically introduced modifications.^453^ Introduction of Cys into stlassin enabled *S*-glucosylation, *S*-arylation and following sulfur elimination to generate dehydroalanine Michael-addition reactions with thiols garnered thioether derivatives. O-glucosylation of a Tyr variant of stlassin was also generated. Tyrosine selective nitration with dinitroimidazole and 390 nm light irradiation enabled the nitration of MccJ25 and caulonodin IV, with the nitration of the two Tyr steric lock residues of caulonodin IV increasing the thermal stability of the interlocked structure.^454^ Carbethoxylation of MccJ25 His5 only had a minor impact of inhibition of RNAP transcription but significantly hindered antimicrobial activity and could be selectively removed,^449^ opening opportunities for stimuli-responsive antimicrobial activity.

### Chemically Synthesized Rotaxanes Containing Amino Acids

The chemical connectivity and interlocked architecture of lasso peptides has been deciphered in part through comparison to control compounds generated by solid-phase peptide synthesis,^296,455^ but the chemical synthesis of lasso peptides remains an unsolved challenge.^456^ The N-terminal-side chain isopeptide bond was confirmed by comparison to synthetic derivatives with this connectivity and head-to-tail cyclized peptides.^303–305^ However, even with the equivalent connectivity to the naturally occurring lasso peptides, the synthetic derivates cannot be prepared with the interlocked structure,^402,457–459^ demonstrating that the precursor is not predisposed to fold into the correct conformation such that isopeptide bond formation would kinetically trap the MIP.^460–462^ Inspired by the biological activity of naturally occurring lasso peptides, noninterlocked constitutional isomers (different cyclic connectivities) have been prepared to investigate their activity as antimicrobials^463–466^ or cancer therapies.^467,468^ Only one synthetic route to an interlocked lasso peptide has been reported, for the disulfide-containing BI-32169 where a covalently attached cryptand-imidazolimium template was used to preorganize the peptide chain such that isopeptide formation trapped the structure into the interlocked [1]­rotaxane architecture.^469^ This isolated example, which has not since been reproduced, highlights the limitations of the total synthesis which may not be generalizable to other lasso peptides.

Given the desire to mimic the architecture of lasso peptides, which display enhanced proteolytic and thermal stability as well as biological activity, many synthetic MIMs have been prepared containing aa residues to confer biological function.^25^ Coutrot and co-workers reported [1]­rotaxane **8** containing a Gly_3_ sequence in the loop which displayed pH-responsive threading based on ammonium-crown ether recognition (Figure 18i).^470^ [1]­Rotaxanes **9** and **10** with aa residues in the loop have also been prepared by first generating a [2]­rotaxane via typical template-directed strategies (amide-hydrogen bonding macrocycle^471^ or ammonium-crown ether)^472^ and then linking the macrocycle to the axle (Figure 18ii). Following this strategy, Saito and Bode showed that short peptide chains could be used to link the components together installed via two amide-forming ligations, the α-ketoacid-hydroxylamine (KAHA) ligation and native chemical ligation.^472^ Synthetic [1]­rotaxanes **10a**–**d** with between 9 and 15 aa (omitting GABA) in the loop were prepared which displayed enhanced proteolytic stability, compared to cyclic and unthreaded control compounds (Figure 18iii).

**Figure 18:**
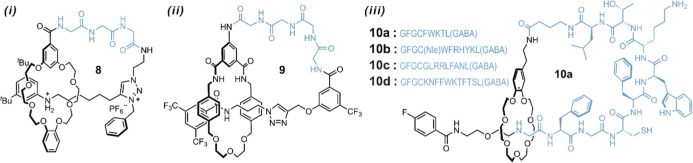
Synthetic [1]­rotaxanes mimicking the structure of lasso peptides. (i) Coutrot’s [1]­rotaxane **8** containing a Gly_3_ sequence in the loop. (ii) Evans’s [1]­rotaxane **9** containing a Gly_4_ sequence in the loop. (iii) Bode’s [1]­rotaxanes were accessed via a [2]­rotaxane precursor allowing various aa sequences (written from N–C termini) to be introduced into the loop (**10a**–**d**), allowing the replication of the lassomycin loop sequence in **10c** and the inclusion of noncanonical amino acids such as norleucine (Nle) and γ-aminobutyric acid (GABA).

Numerous synthetic rotaxanes have been prepared that incorporate aa residues (Figure 19). In 1997, Leigh and co-workers developed a strategy utilizing a Gly–Gly sequence in the axle to template the formation of a benzylic amide macrocycle through hydrogen bonding to garner [2]­rotaxane **11** (Figure 19A).^473^ The protocol has been employed to investigate the preparation of a range of dipeptide containing axles^474–478^ and for the preparation of [2]­rotaxane molecular shuttles.^479–492^ The spacing of the two carbonyls in the Gly–Gly dipeptide is equivalent to the fumaramide recognition unit^493^ which has been widely applied for the preparation of MIMs and for the development of molecular machines. Rotaxanes containing just a single Gly within the structure, as well as those containing the lanthanide binding DOTA motif are beyond the scope of this review.^494–498^ Many cyclodextrin based rotaxanes have also been prepared containing amino acids in the axle^499^ or appended to the cyclodextrin macrocycle, which influence biomedical applications but will not be discussed as they have been recently reviewed.^500,501^ Of note is Anderson and co-workers α-cyclodextrin (α-CD) [2]­rotaxane **17** which was the first enzyme-catalyzed preparation of a synthetic rotaxane (Figure 20).^502^ Inclusion of the azobenzene containing axle in α-CD was promoted in water by the hydrophobic effect and amide coupling between an Arg residue (attached to the azobenzene) and a Phe residue (attached to maleic acid) was mediated by chymotrypsin. Due to the directionality of the axle and cone shape of α-CD there are two potential stereoisomers, with the enzyme selectively forming the thermodynamically less stable stereoisomer under kinetic control.

**Figure 19:**
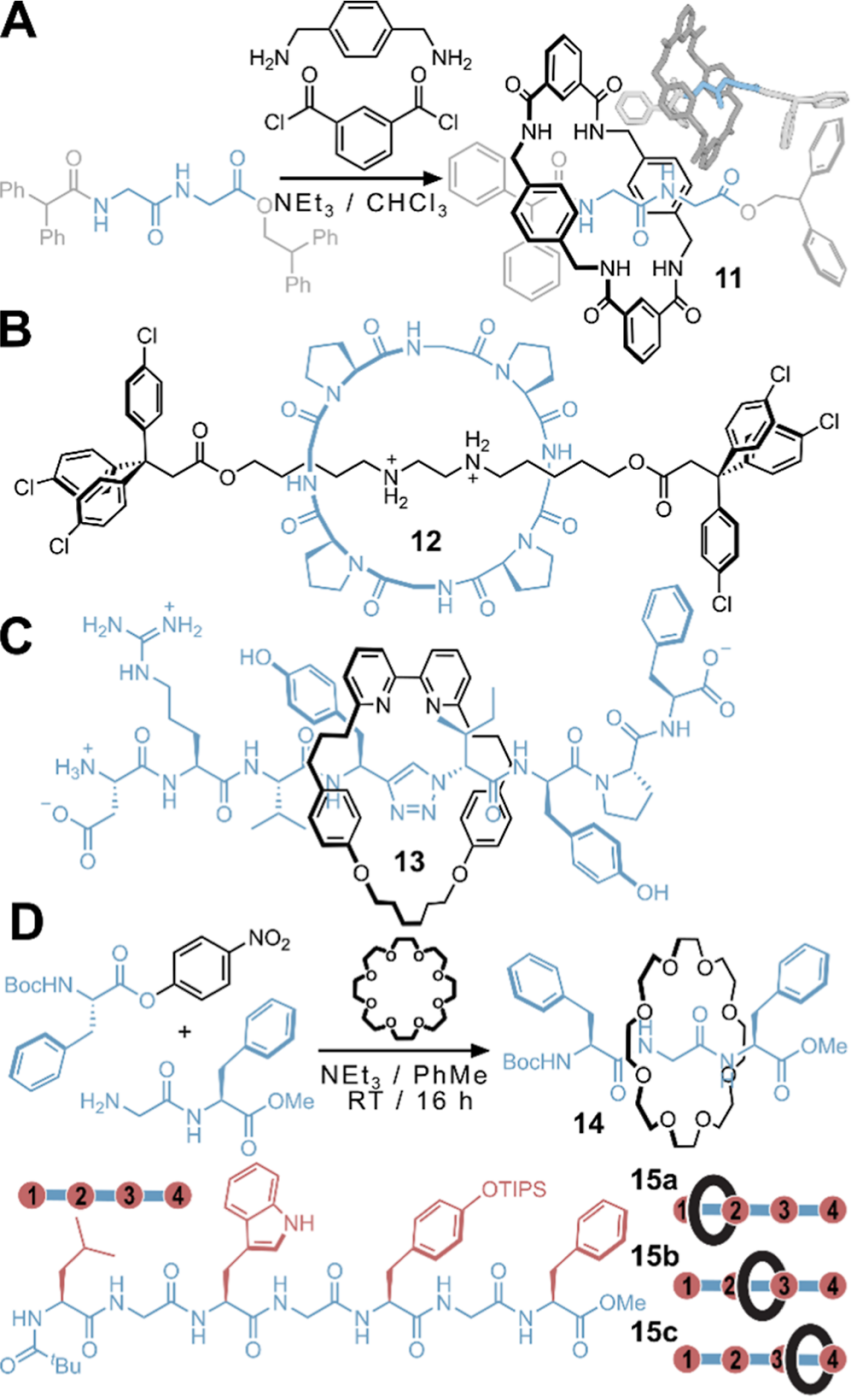
Examples of synthetic rotaxanes containing aa residues. (A) Leigh’s hydrogen bond directed synthesis of diglycine containing [2]­rotaxanes, including the solid-state structure of **11** (CCDC: 123926). (B) Leigh’s cyclo­(l-ProGly)_4_ [2]­rotaxane **12** prepared via ammonium-cyclic peptide recognition and subsequent stoppering. (C) Tian’s triazole isostere peptide [2]­rotaxane **13**. (D) Leigh’s metal-free active template-directed synthesis of crown ether peptide [2]­rotaxane **14** and structure of three positional isomers (**15a**–**c**) of a 24C8-heptapeptide [2]­rotaxane featuring a Boc-Leu-Gly-Trp-Gly-Tyr­(TIPS)-Gly-Phe-OMe axle.

**Figure 20:**
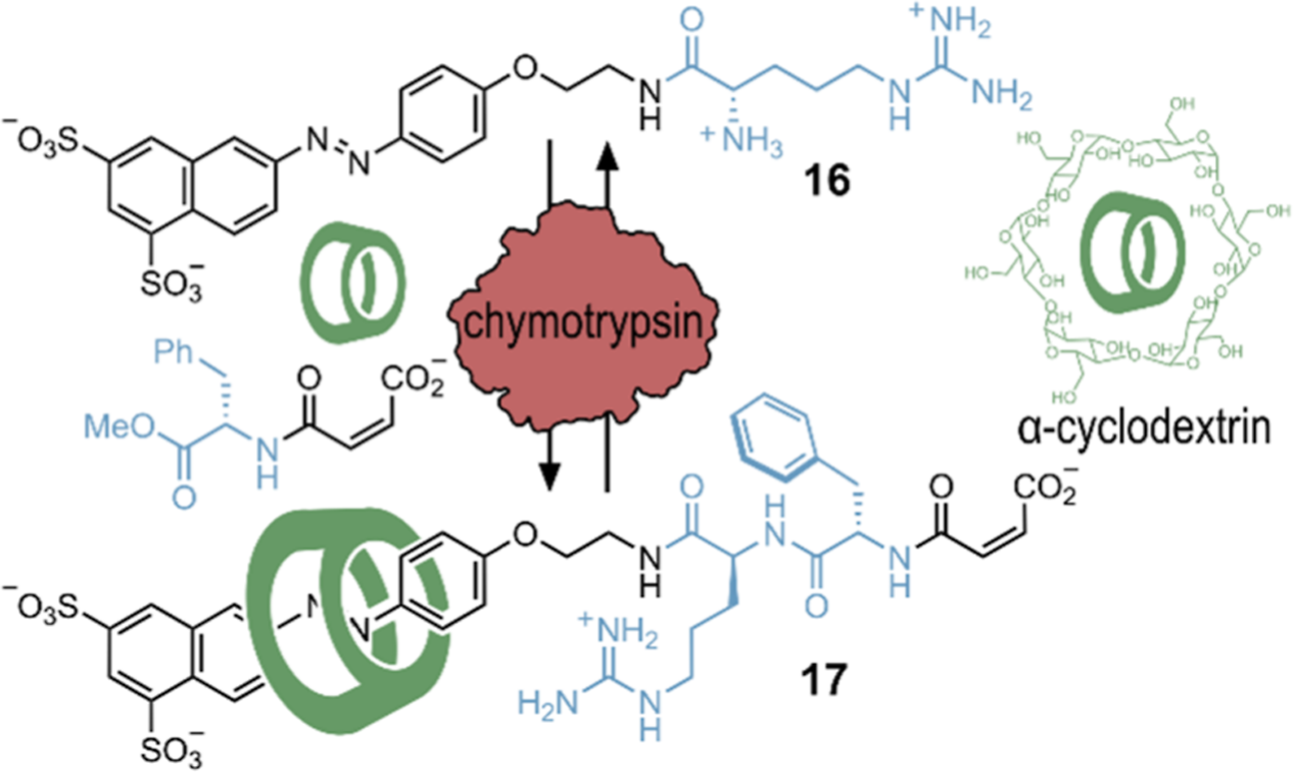
Enzymatically mediated synthesis of an amino acid containing [2]­rotaxane. Anderson and co-workers reported the chymotrypsin mediated synthesis of α-CD [2]­rotaxane **17** in HEPES buffer (pH 8) showing the reversible formation of the interlocked structure from precursor **16**.

Different synthetic strategies have enabled amino acids to be incorporated within various positions of the MIM. In 2006 Leigh and co-workers introduced all amino acid macrocycles cyclo­(l-ProGly)_4_ and cyclo­(l-ProGly)_5_ for the preparation of [2]­rotaxanes (Figure 19B).^503^ The affinity of the peptide macrocycles for ammonium containing axles has been exploited for passive template-directed synthesis of [2]­rotaxanes with nonpeptidic axles such as **12**,^503–505^ but has also been shown to promote the reaction of a Gly primary amine with a Phe activated ester in an active template-directed synthesis of a [2]­rotaxane albeit in low yield (1.2%).^506^ The amide backbone of peptides typically forms intramolecular hydrogen bonds to stabilize folded secondary structures, precluding strong hydrogen bonding interactions to facilitate MIP formation. The use of one nonpeptidic component (macrocycle or axle) enables alternative noncovalent interactions to be employed to promote threading of components, for example Pd^II^ coordination by a 2,6-diamidopyrdine-based macrocycle and the carbonyl of a peptide backbone promotes pseudorotaxane formation,^507^ providing a strategy toward future interlocked MIPs. Papot and co-workers prepared a [1]­rotaxane via AT-CuAAC and showed that the stereogenic center of a Phe residue in the loop directed the diastereoselective synthesis of the mechanically planar chiral rotaxane.^508,509^ Tian and co-workers recently reported the used of AT-CuAAC with a bipyridine-based macrocycle to prepare the small peptide [2]­rotaxane **13** where the linking amide bond was replaced with the triazole ring bioisostere (Figure 19C).^510^ Leigh and co-workers have also recently demonstrated that a range of crown ether–peptide [2]­rotaxanes can be prepared in moderate yield.^511^ The 24-crown-8 macrocycle was shown to promote amide coupling between a glycine primary amine and an aa activated ester in an active template-directed strategy such as **14** (Figure 19D), enabling each of the three positional isomers of a 24C8-heptapeptide (Leu-Gly-Trp-Gly-Tyr-Gly-Phe) [2]­rotaxane **15a**–**c** to be prepared.

Synthetic rotaxanes have displayed a number of functional activities as a result of the amino acids integrated into the architecture (Figure 21).^512^ Smithrud and co-workers reported a [2]­rotaxane with two Arg residues appended to the macrocycle that was capable of transporting peptide cargos across cellular membranes.^513^ Several derivatives were prepared and investigated for transport activity which have been recently reviewed, along with other MIMs capable of transmembrane transport.^514^ Rotaxanation has also been used as a strategy to mechanically protect a cargo, which can be released after programmed decomposition. Leigh, Aucagne, Papot and co-workers reported [2]­rotaxane **18** with a Met-enkephalin pentapeptide (Tyr-Gly-Gly-Phe-Met) axle that displayed enhanced proteolytic stability as a result of mechanical protection, but which could be selectively released after β-galactosidase cleavage of a monosaccharide stopper (Figure 21A).^515^ Subsequently, a β-galactosidase susceptible macrocycle was developed to protect a Gly_3_ tripeptide sequence^516^ and enabled the controlled release of paclitaxel in KB, H661 and MDA-MB-231 cancer cells.^517^

**Figure 21:**
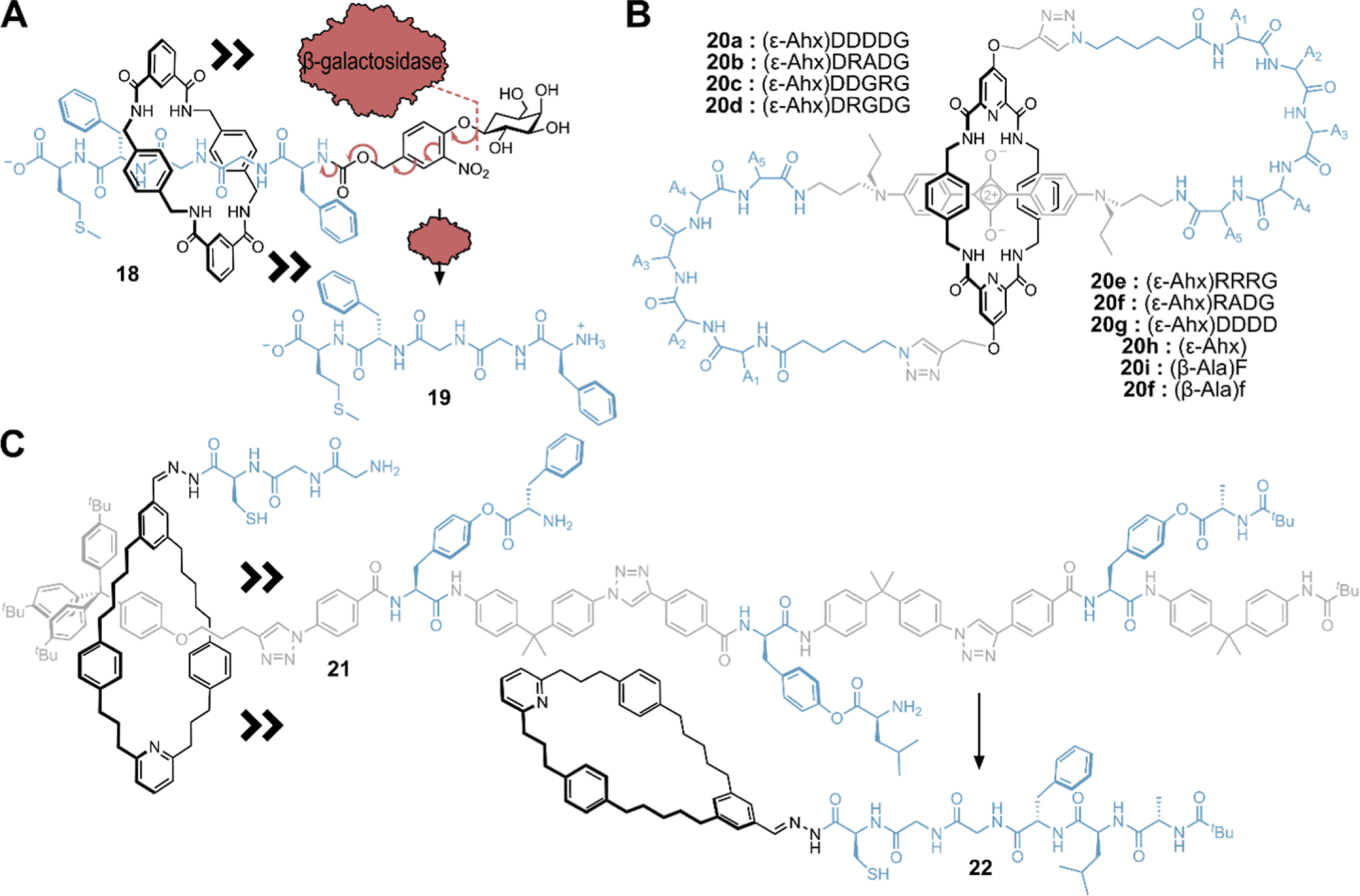
Functional peptide-based rotaxanes. (A) β-Galactosidase responsive mechanically protected peptide [2]­rotaxane **18**, that undergoes self-immolative decomposition (red arrows) after cleavage of the monosaccharide stopper to release pentapeptide **19**. (B) Smith’s figure-of-eight fluorescent MIPs (**20a**–**f**) with different peptide sequences (written from N–C termini) with noncanonical amino acids ε-aminohexanoic acid (ε-Ahx), β-alanine (β-Ala) and d-phenylalanine (f) integrated into the loop regions. (C) Leigh’s peptide synthesizer **21**, which forms three peptide bonds via NCL as the macrocycle processes along the axle. This molecular machine mimics the action of the ribosome, synthesizing a peptide in a sequence selective manner.

The use of MIMs as fluorescent sensors is well established, and the integration of amino acids into the architecture has been shown to enable chiral sensing.^518^ Circularly polarized luminescence from a [2]­rotaxane probe has been shown to depend on the stereogenic center of a Phe residue in the axle which transfers chiral information to a dynamic axially chiral bipyrene-based macrocycle to control the polarity of the Cotton effect.^519^ Integrating short peptide sequences into interlocked fluorescent probes has enabled tunable biological targeting.^520,521^ Smith and co-workers showed that an interlocked squaraine fluorophore could be coupled at each end of the axle to the macrocycle with a peptide linker (1–6 aa) providing a general strategy to figure-of-eight [1]­rotaxanes **20a**–**f** (Figure 21B).^522^ The peptide sequence both increased the stability of the interlocked architecture and enabled the preparation of probes with tailored biological targeting for cell membranes, bone, cancer cells.^523,524^ Interestingly, the mitochondrial accumulation of a figure-of-eight probe with a single Phe in each loop was shown to be dependent on the chirality of these residues (**20i** and **20f**).^525^

Catalytically active aa-containing [2]­rotaxanes have been reported, employed as an organocatalyst for an asymmetric Michael addition reaction.^526^ However, perhaps the most inspiring example is the sequence-specific peptide synthesizer reported by Leigh and co-workers in 2013 (Figure 21C).^16^ Here, the molecular shuttling motion of the macrocycle along the axle of [2]­rotaxane **21** controls the iterative ligation of aa residues onto the macrocycle as it translates down the axle where amino acids are loaded, mimicking the function of the ribosome. After subsequent native chemical ligations, the macrocycle is released from the axle with the synthesized peptide appended and the sequence determined by the position along the axle from which the aa came. Four sequential aa ligations along a single axle is the current maximum achieved for sequence selective synthesis of α-peptides,^527,528^ while β-peptide trimers could also be prepared which are not typically accepted as substrates of the ribosome.^529^ Leu oligomers up to dodecapeptides were also prepared in an equivalent way as the macrocycle translated down a leucine-ester-derivatized polystyrene chain, yielding an α-helix that served as an asymmetric catalyst for the Juliá–Colonna epoxidation of chalcones.^530^

Proteins have been decorated with MIMs, either through avidin–biotin noncovalent association^531^ or covalently linked by cysteine-maleimide conjugation,^532,533^ photoinduced cross-linking^534,535^ or cucurbituril-promoted azide–alkyne cycloaddition.^536,537^ Beyond reports highlighting the application of a new coupling strategy, proteins functionalized with a synthetic MIM have typically been used as fluorescent probes. Holland and d’Orchymont reported a [4]­rotaxane prepared via cooperative capture that had a central cyclodextrin macrocycle with a pendant aryl azide for photoinduced cross-linking.^534^ Coupling the [4]­rotaxane with the trastuzumab monoclonal antibody that binds the human epidermal growth-factor receptor 2 (HER2/neu) overexpressed at the surface of cancer cells enabled tumor localization for PET imaging in vivo.

## Future Perspectives: Challenges, Opportunities and Applications

Naturally occurring MIPs have been discovered with an array of interlocked architectures and complex topologies. These structures have been found to span all ranges of hierarchical protein assembly; from the [1]­rotaxane lasso peptides mimicking secondary structures, Möbius topology cysteine knots forming a small tertiary structure, and [72]­catenane viral capsids assembled at the quaternary structure level. The growing structural diversity highlights the ubiquity of interlocked architectures in nature, however the open question of why nature chose the mechanical bond remains unanswered. The ability to orient aa side chains into new conformations in an interlocked structure is likely a key benefit to entangled protein active sites and for the binding of interlocked secondary metabolites to their target protein. Commonly, MIPs display enhanced proteolytic and thermal stability relative to their untangled components, which may have been a selective pressure for the evolution of these architectures. It will be exciting to see what topological complexity awaits discovery in nature.

Unbeknownst to the original molecular topologists, mechanically interlocked biomolecules now serve as inspiring molecular targets for chemists.^538,539^ Whether it is a wholly synthetic or biological approach for the preparation of new MIPs, significant advances in both have been made only recently opening up the field for the design and preparation of functional MIPs.^17,540^ New interlocked architectures made synthetically such as perplexanes and a trefoil-of-trefoils triskelion knot currently outpace the development of MIP architectures.^541–543^ However, the growing predictability of protein structure and assembly by design,^544^ coupled with the development of new ligation strategies^545–547^ is expected to meet this challenge. The unique benefit to the development of biologically produced MIPs is access to bioengineering technologies including the ability to screen vast libraries of derivatives in a high-throughput manner for biological function.^425^ Hybrid derivatives, comprised of synthetic components or other biomolecules are likely to be a future avenue for diversification, especially interfacing with DNA-based components which have already begun to be explored for the preparation of hybrid MIMs and molecular machines.^548–550^ The ability to interface MIPs with biology for biomedical applications^551^ has already generated two FDA-approved mechanically interlocked drugs based on the cysteine knot, with the use of lasso peptides as a drug scaffold also being hotly pursued.^427^

The sophistication of biology has always inspired chemists, and the out-of-equilibrium operation of biological machinery which displays controlled molecular motions to perform work are a prime example. Top-down approaches in biology have been successful in engineering biological machines such as the ribosome and myosin motors to achieve nonequilibrium functions.^552–555^ The propensity of interlocked components to display controlled mechanical motion inspires a bottom-up approach to the development of peptide-based molecular machines, which may provide complementary applications to their naturally occurring counterparts. The interlocked catenane topology of the HK97 viral capsid provides enhanced strength but also flexibility to accommodate viral DNA. It is, however, typical of naturally occurring MIPs to have conformationally static structures and so the engineering of more dynamic MIPs is sure to be an area in which we can expect future research for the development of biocompatible molecular machines.^556^

## References

[1] Stoddart J. F. (2009). The Chemistry
of the Mechanical Bond. Chem. Soc. Rev..

[2] Sluysmans D., Stoddart J. F. (2019). The Burgeoning of
Mechanically Interlocked Molecules
in Chemistry. Trends Chem..

[3] Langton M. J., Beer P. D. (2014). Rotaxane and Catenane
Host Structures for Sensing Charged
Guest Species. Acc. Chem. Res..

[4] Lewis J. E. M., Galli M., Goldup S. M. (2017). Properties
and Emerging Applications
of Mechanically Interlocked Ligands. Chem. Commun..

[5] Heard A. W., Goldup S. M. (2020). Simplicity in the
Design, Operation, and Applications
of Mechanically Interlocked Molecular Machines. ACS Cent. Sci..

[6] Gao Y., Gupta P., Rončević I., Mycroft C., Gates P. J., Parker A. W., Anderson H. L. (2025). Solution-Phase Stabilization
of a Cyclocarbon by Catenane Formation. Science.

[7] Langton M. J., Robinson S. W., Marques I., Félix V., Beer P. D. (2014). Halogen Bonding in Water Results
in Enhanced Anion
Recognition in Acyclic and Rotaxane Hosts. Nat.
Chem..

[8] Barendt T.
A., Docker A., Marques I., Félix V., Beer P. D. (2016). Selective Nitrate
Recognition by a Halogen-Bonding
Four-Station [3]­Rotaxane Molecular Shuttle. Angew. Chem., Int. Ed..

[9] Hoekman S., Kitching M. O., Leigh D. A., Papmeyer M., Roke D. (2015). Goldberg Active
Template Synthesis of a [2]­Rotaxane Ligand for Asymmetric Transition-Metal
Catalysis. J. Am. Chem. Soc..

[10] Galli M., Lewis J. E. M., Goldup S. M. (2015). A Stimuli-Responsive
Rotaxane-Gold
Catalyst: Regulation of Activity and Diastereoselectivity. Angew. Chem., Int. Ed..

[11] August D. P., Borsley S., Cockroft S. L., della Sala F., Leigh D. A., Webb S. J. (2020). Transmembrane Ion Channels Formed
by a Star of David [2]­Catenane and a Molecular Pentafoil Knot. J. Am. Chem. Soc..

[12] Min
Tay H., Johnson T. G., Docker A., Langton M. J., Beer P. D. (2023). Exploiting
the Catenane Mechanical Bond Effect for Selective Halide Anion Transmembrane
Transport. Angew. Chem., Int. Ed..

[13] Bąk K. M., Kisiel O., Mondal D., Zimnicka M. M., Chmielewski M. J. (2025). Anion Transport
across Lipid Bilayers by a Hydrogen Bonding Homo[2]­Catenane. Chem. Commun..

[14] Wang C., Wang S., Yang H., Xiang Y., Wang X., Bao C., Zhu L., Tian H., Qu D. H. (2021). A Light-Operated
Molecular Cable Car for Gated Ion Transport. Angew. Chem., Int. Ed..

[15] Green J. E., Wook Choi J., Boukai A., Bunimovich Y., Johnston-Halperin E., Deionno E., Luo Y., Sheriff B. A., Xu K., Shik Shin Y., Tseng H. R., Stoddart J. F., Heath J. R. (2007). A 160-Kilobit
Molecular Electronic Memory Patterned at 1011 Bits per Square Centimetre. Nature.

[16] Lewandowski B., De Bo G., Ward J. W., Papmeyer M., Kuschel S., Aldegunde M. J., Gramlich P. M. E., Heckmann D., Goldup S. M., D’Souza D. M., Fernandes A. E., Leigh D. A. (2013). Sequence-Specific
Peptide Synthesis by an Artificial Small-Molecule Machine. Science.

[17] Beeren S. R., McTernan C. T., Schaufelberger F. (2023). The Mechanical Bond in Biological
Systems. Chem.

[18] Riebe J., Niemeyer J. (2021). Mechanically Interlocked Molecules
for Biomedical Applications. Eur. J. Org. Chem..

[19] Forgan R. S., Sauvage J. P., Stoddart J. F. (2011). Chemical
Topology: Complex Molecular
Knots, Links, and Entanglements. Chem. Rev..

[20] Zhang W.-B., Sun F., Tirrell D. A., Arnold F. H. (2013). Controlling Macromolecular Topology
with Genetically Encoded SpyTag-SpyCatcher Chemistry. J. Am. Chem. Soc..

[21] Ahnert S. E., Marsh J. A., Hernández H., Robinson C. V., Teichmann S. A. (2015). Principles
of Assembly Reveal a Periodic Table of Protein Complexes. Science.

[22] Francl M. (2009). Stretching
Topology. Nat. Chem..

[23] Alexander J. W., Briggs G. B. (1926). On Types of Knotted Curves. Ann.
Math..

[24] Walba D. M. (1985). Topological
Stereochemistry. Tetrahedron.

[25] Spicer R. L., Evans N. H. (2025). Pretzelanes, [1]­Rotaxanes
and Molecular Figures-of-Eight
- Crossing the Bridge from Fundamentals to Functional Applications. Org. Biomol. Chem..

[26] Adams C., Devadoss J., Elhamdadi M., Mashaghi A. (2020). Knot Theory for Proteins:
Gauss Codes, Quandles and Bondles. J. Math.
Chem..

[27] Wasserman E. (1960). The Preparation
of Interlocking Rings: A Catenane. J. Am. Chem.
Soc..

[28] Schill G., Lüttringhaus A. (1964). The Preparation of Catena Compounds by Directed Synthesis. Angew. Chem., Int. Ed. Engl..

[29] Harrison I. T., Harrison S. (1967). Synthesis of a Stable Complex of a Macrocycle and a
Threaded Chain. J. Am. Chem. Soc..

[30] Hudson B., Vinograd J. (1967). Catenated Circular
DNA Molecules in HeLa Cell Mitochondria. Nature.

[31] Clayton D. A., Vinograd J. (1967). Circular Dimer and
Catenate Forms of Mitochondrial
DNA in Human Leukaemic Leucocytes. Nature.

[32] Gellert M., Mizuuchi K., O’Dea M. H., Nash H. A. (1976). DNA Gyrase: An Enzyme
That Introduces Superhelical Turns into DNA. Proc. Natl. Acad. Sci. U. S. A..

[33] Dean F. B., Stasiak A., Koller T., Cozzarelli N. R. (1985). Duplex
DNA Knots Produced by Escherichia coli Topoisomerase I. Structure
and Requirements for Formation. J. Biol. Chem..

[34] Worcel A., Burgi E. (1972). On the Structure of
the Folded Chromosome of Escherichia coli. J.
Mol. Biol..

[35] Hardy C. D., Crisona N. J., Stone M. D., Cozzarelli N. R. (2004). Disentangling
DNA during Replication: A Tale of Two Strands. Philos. Trans. R. Soc., B.

[36] Sundin O., Varshavsky A. (1980). Terminal Stages of SV40 DNA Replication Proceed via
Multiply Intertwined Catenated Dimers. Cell.

[37] Liu D., Chen G., Akhter U., Cronin T. M., Weizmann Y. (2016). Creating Complex
Molecular Topologies by Configuring DNA Four-Way Junctions. Nat. Chem..

[38] Chen J., Seeman N. C. (1991). Synthesis from DNA of a Molecule with the Connectivity
of a Cube. Nature.

[39] Jester S. S., Famulok M. (2014). Mechanically Interlocked DNA Nanostructures for Functional
Devices. Acc. Chem. Res..

[40] Lu C. H., Cecconello A., Elbaz J., Credi A., Willner I. (2013). A Three-Station
DNA Catenane Rotary Motor with Controlled Directionality. Nano Lett..

[41] Lim N. C. H., Jackson S. E. (2015). Molecular Knots in Biology and Chemistry. J. Phys.: Condens. Matter.

[42] Niemyska W., Mukherjee S., Gren B. A., Niewieczerzal S., Bujnicki J. M., Sulkowska J. I. (2024). Discovery
of a Trefoil Knot in the
RydC RNA: Challenging Previous Notions of RNA Topology. J. Mol. Biol..

[43] Stoddart J. F. (2017). Mechanically
Interlocked Molecules (MIMs)-Molecular Shuttles, Switches, and Machines
(Nobel Lecture). Angew. Chem., Int. Ed..

[44] Sauvage J.-P. (2017). From Chemical
Topology to Molecular Machines (Nobel Lecture). Angew. Chem., Int. Ed..

[45] Feringa B. L. (2017). The Art
of Building Small: From Molecular Switches to Motors (Nobel Lecture). Angew. Chem., Int. Ed..

[46] Mansfield M. L. (1994). Are There
Knots in Proteins?. Nat. Struct. Biol..

[47] Richardson J. S. (1977). β-Sheet
Topology and the Relatedness of Proteins. Nature.

[48] Liljas A., Kannan K. K., Bergstén P. C., Waara I., Fridborg K., Strandberg B., Carlbom U., Järup L., Lövgren S., Petef M. (1972). Crystal Structure of Human Carbonic
Anhydrase C. Nat. New Biol..

[49] Sulkowska J. I. (2020). On Folding
of Entangled Proteins: Knots, Lassos, Links and θ-Curves. Curr. Opin. Struct. Biol..

[50] Dabrowski-Tumanski P., Rubach P., Goundaroulis D., Dorier J., Sułkowski P., Millett K. C., Rawdon E. J., Stasiak A., Sulkowska J. I. (2019). KnotProt
2.0: A Database of Proteins with Knots and Other Entangled Structures. Nucleic Acids Res..

[51] Jamroz M., Niemyska W., Rawdon E. J., Stasiak A., Millett K. C., Sułkowski P., Sulkowska J. I. (2015). KnotProt: A Database of Proteins
with Knots and Slipknots. Nucleic Acids Res..

[52] Niemyska W., Rubach P., Gren B. A., Nguyen M. L., Garstka W., Bruno Da Silva F., Rawdon E. J., Sulkowska J. I. (2022). AlphaKnot:
Server to Analyze Entanglement in Structures Predicted by AlphaFold
Methods. Nucleic Acids Res..

[53] Rubach P., Sikora M., Jarmolinska A. I., Perlinska A. P., Sulkowska J. I. (2024). AlphaKnot 2.0: A Web Server for the
Visualization of
Proteins’ Knotting and a Database of Knotted AlphaFold-Predicted
Models. Nucleic Acids Res..

[54] Bölinger D., Sułkowska J. I., Hsu H. P., Mirny L. A., Kardar M., Onuchic J. N., Virnau P. (2010). A Stevedore’s
Protein Knot. PLoS Comput. Biol..

[55] Schmidberger J. W., Wilce J. A., Weightman A. J., Whisstock J. C., Wilce M. C. J. (2008). The Crystal Structure of DehI Reveals
a New α-Haloacid
Dehalogenase Fold and Active-Site Mechanism. J. Mol. Biol..

[56] Brems M. A., Runkel R., Yeates T. O., Virnau P. (2022). AlphaFold
Predicts
the Most Complex Protein Knot and Composite Protein Knots. Protein Sci..

[57] Hsu M. F., Sriramoju M. K., Lai C. H., Chen Y. R., Huang J. S., Ko T. P., Huang K. F., Hsu S. T. D. (2024). Structure, Dynamics,
and Stability of the Smallest and Most Complex 71 Protein Knot. J. Biol. Chem..

[58] Tubiana L., Alexander G. P., Barbensi A., Buck D., Cartwright J. H. E., Chwastyk M., Cieplak M., Coluzza I., Čopar S., Craik D. J., Di Stefano M., Everaers R., Faísca P. F. N., Ferrari F., Giacometti A., Goundaroulis D., Haglund E., Hou Y. M., Ilieva N., Jackson S. E., Japaridze A., Kaplan N., Klotz A. R., Li H., Likos C. N., Locatelli E., López-León T., Machon T., Micheletti C., Michieletto D., Niemi A., Niemyska W., Niewieczerzal S., Nitti F., Orlandini E., Pasquali S., Perlinska A. P., Podgornik R., Potestio R., Pugno N. M., Ravnik M., Ricca R., Rohwer C. M., Rosa A., Smrek J., Souslov A., Stasiak A., Steer D., Sułkowska J., Sułkowski P., Sumners D. W. L., Svaneborg C., Szymczak P., Tarenzi T., Travasso R., Virnau P., Vlassopoulos D., Ziherl P., Žumer S. (2024). Topology in
Soft and Biological Matter. Phys. Rep..

[59] Dabrowski-Tumanski P., Sulkowska J. I. (2017). To Tie
or Not to Tie? That Is the Question. Polymers.

[60] Hsu S. T. D. (2023). Folding
and Functions of Knotted Proteins. Curr. Opin.
Struct. Biol..

[61] Banani S. F., Lee H. O., Hyman A. A., Rosen M. K. (2017). Biomolecular Condensates:
Organizers of Cellular Biochemistry. Nat. Rev.
Mol. Cell Biol..

[62] Das D., Deniz A. A. (2023). Topological Considerations in Biomolecular Condensation. Biomolecules.

[63] Shin Y., Brangwynne C. P. (2017). Liquid
Phase Condensation in Cell Physiology and Disease. Science.

[64] Liang C., Mislow K. (1995). Topological Features
of Protein Structures: Knots and
Links. J. Am. Chem. Soc..

[65] Dabrowski-Tumanski P., Goundaroulis D., Stasiak A., Rawdon E. J., Sulkowska J. I. (2024). Theta-Curves
in Proteins. Protein Sci..

[66] Moriuchi H. (2009). A Table of
θ-Curves and Handcuff Graphs with up to Seven Crossings. Adv. Stud. Pure Math..

[67] Liang C., Mislow K. (1994). Knots in Proteins. J. Am. Chem.
Soc..

[68] Mallam A. L., Jackson S. E. (2007). A Comparison of the Folding of Two Knotted Proteins:
YbeA and YibK. J. Mol. Biol..

[69] Rivera M., Hao Y., Maillard R. A., Baez M. (2020). Mechanical Unfolding of a Knotted
Protein Unveils the Kinetic and Thermodynamic Consequences of Threading
a Polypeptide Chain. Sci. Rep..

[70] Norcross T. S., Yeates T. O. (2006). A Framework for Describing Topological
Frustration
in Models of Protein Folding. J. Mol. Biol..

[71] Sułkowska J. I., Sułkowski P., Szymczak P., Cieplak M. (2008). Stabilizing Effect
of Knots on Proteins. Proc. Natl. Acad. Sci.
U. S. A..

[72] Yeates T. O., Norcross T. S., King N. P. (2007). Knotted and Topologically Complex
Proteins as Models for Studying Folding and Stability. Curr. Opin. Chem. Biol..

[73] Wagner J. R., Brunzelle J. S., Forest K. T., Vierstra R. D. (2005). A Light-Sensing
Knot Revealed by the Structure of the Chromophore-Binding Domain of
Phytochrome. Nature.

[74] Sayre T. C., Lee T. M., King N. P., Yeates T. O. (2011). Protein
Stabilization
in a Highly Knotted Protein Polymer. Protein
Eng. Des. Sel..

[75] King N. P., Yeates E. O., Yeates T. O. (2007). Identification of Rare Slipknots
in Proteins and Their Implications for Stability and Folding. J. Mol. Biol..

[76] Hsu S. T. D., Lee Y. T. C., Mikula K. M., Backlund S. M., Tascón I., Goldman A., Iwaï H. (2021). Tying up the
Loose Ends: A Mathematically
Knotted Protein. Front. Chem..

[77] King N. P., Jacobitz A. W., Sawaya M. R., Goldschmidt L., Yeates T. O. (2010). Structure and Folding of a Designed Knotted Protein. Proc. Natl. Acad. Sci. U. S. A..

[78] Doyle L. A., Takushi B., Kibler R. D., Milles L. F., Orozco C. T., Jones J. D., Jackson S. E., Stoddard B. L., Bradley P. (2023). De Novo Design
of Knotted Tandem Repeat Proteins. Nat. Commun..

[79] Minami S., Kobayashi N., Sugiki T., Nagashima T., Fujiwara T., Tatsumi-Koga R., Chikenji G., Koga N. (2023). Exploration
of Novel Αβ-Protein Folds through de Novo Design. Nat. Struct. Mol. Biol..

[80] Dietrich-Buchecker C. O., Sauvage J.-P. (1989). A Synthetic Molecular
Trefoil Knot. Angew. Chem., Int. Ed. Engl..

[81] Chambron J. C., Dietrich-Buchecker C. O., Nierengarten J. F., Sauvage J. P. (1994). Transition Metals as Assembling and
Templating Species:
From Catenanes and Knots to Strings Threaded through Molecular Rings. Pure Appl. Chem..

[82] Fielden S. D. P., Leigh D. A., Woltering S. L. (2017). Molecular Knots. Angew. Chem., Int. Ed..

[83] Ashbridge Z., Fielden S. D. P., Leigh D. A., Pirvu L., Schaufelberger F., Zhang L. (2022). Knotting Matters: Orderly Molecular Entanglements. Chem. Soc. Rev..

[84] Ponnuswamy N., Cougnon F. B. L., Pantoş G. D., Sanders J. K. M. (2014). Homochiral and
Meso Figure Eight Knots and a Solomon Link. J. Am. Chem. Soc..

[85] Feigel M., Ladberg R., Engels S., Herbst-Irmer R., Fröhlich R. (2006). A Trefoil Knot Made of Amino Acids and Steroids. Angew. Chem., Int. Ed..

[86] Song Y., Schaufelberger F., Ashbridge Z., Pirvu L., Vitorica-Yrezabal I. J., Leigh D. A. (2021). Effects of Turn-Structure on Folding and Entanglement
in Artificial Molecular Overhand Knots. Chem.
Sci..

[87] Zhang Z. H., Zhou Q., Li Z., Zhang N., Zhang L. (2023). Completely
Stereospecific Synthesis of a Molecular Cinquefoil (51) Knot. Chem.

[88] Zhao X., Yang R., Zhang Z. H., Zhu T., Zhang L. (2023). Theoretical
Investigation of the Lack of Chiral Self-Sorting Behaviour of a Molecular
Cinquefoil Knot. Supramol. Chem..

[89] Zhou A., Yang R., Zhang Z. H., Zhang L. (2025). Remote Control of Synthetic
Knots through Peptide Sequences. Sci. China
Chem..

[90] Yang R., Zhao X., Zhang Z. H., Zhang L. (2026). Inducing Chiral Order
in a Synthetic Knotted System. Chem.

[91] Jeong S., Lee K., Yoo S. H., Lee H. S., Kwon S. (2023). Crystalline Metal-Peptide
Networks: Structures, Applications, and Future Outlook. ChemBioChem.

[92] Sawada T., Matsumoto A., Fujita M. (2014). Coordination-Driven Folding and Assembly
of a Short Peptide into a Protein-like Two-Nanometer-Sized Channel. Angew. Chem., Int. Ed..

[93] Sawada T., Fujita M. (2021). Orderly Entangled Nanostructures of Metal-Peptide Strands. Bull. Chem. Soc. Jpn..

[94] Inomata Y., Sawada T., Fujita M. (2020). Metal-Peptide Torus Knots from Flexible
Short Peptides. Chem.

[95] Inomata Y., Sawada T., Fujita M. (2021). Metal-Peptide
Nonafoil Knots and
Decafoil Supercoils. J. Am. Chem. Soc..

[96] Oguma S., Inomata Y., Hayakawa A., Nakama T., Fujita M., Sawada T. (2025). Helical Sense Control
of Metal-Peptide Torus Frameworks
Leading to the Folding and Assembly of a Ag21L14 Braided Peptide Nanotube. J. Am. Chem. Soc..

[97] Wang X. W., Zhang W.-B. (2018). Chemical Topology and Complexity of Protein Architectures. Trends Biochem. Sci..

[98] Wang Y. X., Wu W. H., Jiang F. Y., Zhang W.-B. (2024). Mechanically
Interlocked
Biomacromolecules. Supramol. Mater..

[99] Pieters B. J. G. E., Van Eldijk M. B., Nolte R. J. M., Mecinović J. (2016). Natural Supramolecular
Protein Assemblies. Chem. Soc. Rev..

[100] Cao Z., Roszak A. W., Gourlay L. J., Lindsay J. G., Isaacs N. W. (2005). Bovine
Mitochondrial Peroxiredoxin III Forms a Two-Ring Catenane. Structure.

[101] Cao Z., McGow D. P., Shepherd C., Lindsay J. G. (2015). Improved
Catenated
Structures of Bovine Peroxiredoxin III F190L Reveal Details of Ring-Ring
Interactions and a Novel Conformational State. PLoS One.

[102] Smeulders M. J., Barends T. R. M., Pol A., Scherer A., Zandvoort M. H., Udvarhelyi A., Khadem A. F., Menzel A., Hermans J., Shoeman R. L., Wessels H. J. C. T., Van Den Heuvel L. P., Russ L., Schlichting I., Jetten M. S. M., Op
Den Camp H. J. M. (2011). Evolution of a New Enzyme for Carbon
Disulphide Conversion by an Acidothermophilic Archaeon. Nature.

[103] Van Eldijk M. B., van Leeuwen I., Mikhailov V. A., Neijenhuis L., Harhangi H. R., van Hest J. C. M., Jetten M. S. M., Op Den Camp H. J. M., Robinson C. V., Mecinović J. (2013). Evidence That
the Catenane Form of CS2 Hydrolase Is Not an Artefact. Chem. Commun..

[104] Van
Eldijk M. B., Pieters B. J., Mikhailov V. A., Robinson C. V., Van Hest J. C. M., Mecinović J. (2014). Catenane versus
Ring: Do Both Assemblies of CS2 Hydrolase Exhibit the Same Stability
and Catalytic Activity?. Chem. Sci..

[105] Wang J. C. Y., Zlotnick A., Mecinović J. (2014). Transmission
Electron Microscopy Enables the Reconstruction of the Catenane and
Ring Forms of CS2 Hydrolase. Chem. Commun..

[106] Smeulders M. J., Pol A., Venselaar H., Barends T. R. M., Hermans J., Jetten M. S. M., Op
den Camp H. J. M. (2013). Bacterial CS2 Hydrolases from Acidithiobacillus Thiooxidans
Strains Are Homologous to the Archaeal Catenane Cs2 Hydrolase. J. Bacteriol..

[107] Zimanyi C. M., Ando N., Brignole E. J., Asturias F. J., Stubbe J., Drennan C. L. (2012). Tangled Up in Knots: Structures of
Inactivated Forms of E. coli Class Ia Ribonucleotide Reductase. Structure.

[108] Lee B., Kim K. H., Park S. J., Eom S. H., Song H. K., Suh S. W. (2004). Ring-Shaped Architecture of RecR:
Implications for
Its Role in Homologous Recombinational DNA Repair. EMBO J..

[109] Domínguez-Gil T., Molina R., Dik D. A., Spink E., Mobashery S., Hermoso J. A. (2017). X-Ray Structure
of Catenated Lytic
Transglycosylase SltB1. Biochemistry.

[110] Song H., Van Der Velden N. S., Shiran S. L., Bleiziffer P., Zach C., Sieber R., Imani A. S., Krausbeck F., Aebi M., Freeman M. F., Riniker S., Künzler M., Naismith J. H. (2018). A Molecular Mechanism
for the Enzymatic Methylation
of Nitrogen Atoms within Peptide Bonds. Sci.
Adv..

[111] Ongpipattanakul C., Nair S. K. (2018). Molecular Basis for Autocatalytic
Backbone N-Methylation in RiPP Natural Product Biosynthesis. ACS Chem. Biol..

[112] Wikoff W. R., Liljas L., Duda R. L., Tsuruta H., Hendrix R. W., Johnson J. E. (2000). Topologically Linked Protein Rings
in the Bacteriophage HK97 Capsid. Science.

[113] Duda R. L. (1998). Protein Chainmail: Catenated Protein
in Viral Capsids. Cell.

[114] Helgstrand C., Wikoff W. R., Duda R. L., Hendrix R. W., Johnson J. E., Liljas L. (2003). The Refined Structure
of a Protein
Catenane: The HK97 Bacteriophage Capsid at 3.44 Å Resolution. J. Mol. Biol..

[115] Johnson J. E. (2010). Virus Particle Maturation: Insights into Elegantly
Programmed Nanomachines. Curr. Opin. Struct.
Biol..

[116] Gertsman I., Gan L., Guttman M., Lee K., Speir J. A., Duda R. L., Hendrix R. W., Komives E. A., Johnson J. E. (2009). An Unexpected Twist in Viral Capsid Maturation. Nature.

[117] Tso D. J., Peebles C. L., Maurer J. B., Duda R. L., Hendrix R. W. (2017). On the Catalytic Mechanism of Bacteriophage HK97 Capsid
Crosslinking. Virology.

[118] Gan L., Speir J. A., Conway J. F., Lander G., Cheng N., Firek B. A., Hendrix R. W., Duda R. L., Liljas L., Johnson J. E. (2006). Capsid Conformational Sampling in
HK97 Maturation Visualized
by X-Ray Crystallography and Cryo-EM. Structure.

[119] Dierkes L. E., Peebles C. L., Firek B. A., Hendrix R. W., Duda R. L. (2009). Mutational Analysis of a Conserved
Glutamic Acid Required
for Self-Catalyzed Cross-Linking of Bacteriophage HK97 Capsids. J. Virol..

[120] Ross P. D., Cheng N., Conway J. F., Firek B. A., Hendrix R. W., Duda R. L., Steven A. C. (2005). Crosslinking Renders
Bacteriophage HK97 Capsid Maturation Irreversible and Effects an Essential
Stabilization. EMBO J..

[121] Lee K. K., Gan L., Tsuruta H., Moyer C., Conway J. F., Duda R. L., Hendrix R. W., Steven A. C., Johnson J. E. (2008). Virus Capsid Expansion Driven by the Capture of Mobile
Surface Loops. Structure.

[122] Gan L., Conway J. F., Firek B. A., Cheng N., Hendrix R. W., Steven A. C., Johnson J. E., Duda R. L. (2004). Control of Crosslinking
by Quaternary Structure Changes during Bacteriophage HK97 Maturation. Mol. Cell.

[123] Huet A., Oh B., Maurer J., Duda R. L., Conway J. F. (2023). A Symmetry Mismatch Unraveled: How Phage HK97 Scaffold
Flexibly Accommodates a 12-Fold Pore at a 5- Fold Viral Capsid Vertex. Sci. Adv..

[124] Belford A. K., Maurer J. B., Duda R. L., Huet A., Conway J. F. (2025). Structural Insights into Scaffold-Guided Assembly of
the Pseudomonas Phage D3 Capsid. Nat. Commun..

[125] Hardy J. M., Dunstan R. A., Grinter R., Belousoff M. J., Wang J., Pickard D., Venugopal H., Dougan G., Lithgow T., Coulibaly F. (2020). The Architecture
and Stabilisation of Flagellotropic Tailed Bacteriophages. Nat. Commun..

[126] Zhang X., Guo H., Jin L., Czornyj E., Hodes A., Hui W. H., Nieh A. W., Miller J. F., Zhou Z. H. (2013). A New Topology of the HK97-like Fold
Revealed in Bordetella
Bacteriophage by CryoEM at 3.5 Å Resolution. eLife.

[127] Zhou Z. H., Chiou J. (2015). Protein Chainmail Variants
in DsDNA
Viruses. AIMS Biophys..

[128] Kang S., Hawkridge A. M., Johnson K. L., Muddiman D. C., Prevelige P. E. (2006). Identification
of Subunit-Subunit Interactions in Bacteriophage
P22 Procapsids by Chemical Cross-Linking and Mass Spectrometry. J. Proteome Res..

[129] Chavez J. D., Cilia M., Weisbrod C. R., Ju H. J., Eng J. K., Gray S. M., Bruce J. E. (2012). Cross-Linking Measurements
of the Potato Leafroll Virus Reveal Protein Interaction Topologies
Required for Virion Stability, Aphid Transmission, and Virus-Plant
Interactions. J. Proteome Res..

[130] Domínguez-Zotes S., Fuertes M. A., Rodríguez-Huete A., Valbuena A., Mateu M. G. (2022). A Genetically
Engineered, Chain Mail-Like
Nanostructured Protein Material with Increased Fatigue Resistance
and Enhanced Self-Healing. Small.

[131] Aguirre C., Goto Y., Costas M. (2016). Thermal and Chemical
Unfolding Pathways of PaSdsA1 Sulfatase, a Homo-Dimer with Topologically
Interlinked Chains. FEBS Lett..

[132] Banerjee R., Nath S., Ranjan A., Khamrui S., Pani B., Sen R., Sen U. (2012). The First
Structure
of Polarity Suppression Protein, Psu from Enterobacteria Phage P4,
Reveals a Novel Fold and a Knotted Dimer. J.
Biol. Chem..

[133] Mascarenhas N. M., Gosavi S. (2017). Understanding Protein Domain-Swapping
Using Structure-Based Models of Protein Folding. Prog. Biophys. Mol. Biol..

[134] Baiesi M., Orlandini E., Trovato A., Seno F. (2016). Linking in
Domain-Swapped Protein Dimers. Sci. Rep..

[135] Boutz D. R., Cascio D., Whitelegge J., Perry L. J., Yeates T. O. (2007). Discovery of a Thermophilic Protein
Complex Stabilized by Topologically Interlinked Chains. J. Mol. Biol..

[136] Duff A. P., Cohen A. E., Ellis P. J., Kuchar J. A., Langley D. B., Shepard E. M., Dooley D. M., Freeman H. C., Guss J. M. (2003). The Crystal Structure of Pichia Pastoris Lysyl Oxidase. Biochemistry.

[137] Dabrowski-Tumanski P., Sulkowska J. I. (2017). Topological Knots and Links in Proteins. Proc. Natl. Acad. Sci. U. S. A..

[138] Dabrowski-Tumanski P., Jarmolinska A. I., Niemyska W., Rawdon E. J., Millett K. C., Sulkowska J. I. (2017). LinkProt:
A Database Collecting Information
about Biological Links. Nucleic Acids Res..

[139] Xu L., Zhang W.-B. (2018). Topology: A Unique
Dimension in Protein Engineering. Sci. China
Chem..

[140] Qu Z., Cheng S. Z. D., Zhang W.-B. (2021). Macromolecular
Topology Engineering. Trends Chem..

[141] Li T., Zhang F., Fang J., Liu Y., Zhang W.-B. (2023). Rational
Design and Cellular Synthesis of Proteins with Unconventional Chemical
Topology. Chin. J. Chem..

[142] Yan L. Z., Dawson P. E. (2001). Design and Synthesis of a Protein
Catenane. Angew. Chem., Int. Ed..

[143] Hill R. B., Raleigh D. P., Lombardi A., Degrado W. F. (2000). De Novo
Design of Helical Bundles as Models for Understanding Protein Folding
and Function. Acc. Chem. Res..

[144] Davison T. S., Nie X., Ma W., Lin Y., Kay C., Benchimol S., Arrowsmith C. H. (2001). Structure
and Functionality of a
Designed P53 Dimer. J. Mol. Biol..

[145] Blankenship J. W., Dawson P. E. (2003). Thermodynamics of
a Designed Protein
Catenane. J. Mol. Biol..

[146] Blankenship J. W., Dawson P. E. (2007). Threading a Peptide
through a Peptide:
Protein Loops, Rotaxanes, and Knots. Protein
Sci..

[147] Wang X. W., Zhang W.-B. (2016). Cellular Synthesis
of Protein Catenanes. Angew. Chem., Int. Ed..

[148] Zakeri B., Fierer J. O., Celik E., Chittock E. C., Schwarz-Linek U., Moy V. T., Howarth M. (2012). Peptide Tag
Forming
a Rapid Covalent Bond to a Protein, through Engineering a Bacterial
Adhesin. Proc. Natl. Acad. Sci. U. S. A..

[149] Howarth M. R. (2025). Click Biology Highlights the Opportunities
from Reliable
Biological Reactions. Nat. Chem. Biol..

[150] Wang X. W., Zhang W.-B. (2017). Protein Catenation
Enhances Both
the Stability and Activity of Folded Structural Domains. Angew. Chem., Int. Ed..

[151] Bai X., Liu Y., Lee J., Fang J., Wu W. H., Seo J., Zhang W.-B. (2022). Cellular Synthesis of Protein Pretzelanes. Giant.

[152] Liu Y., Duan Z., Fang J., Zhang F., Xiao J., Zhang W.-B. (2020). Cellular Synthesis
and X-Ray Crystal Structure of a
Designed Protein Heterocatenane. Angew. Chem.,
Int. Ed..

[153] Wu W. H., Bai X., Shao Y., Yang C., Wei J., Wei W., Zhang W.-B. (2021). Higher Order Protein Catenation Leads
to an Artificial Antibody with Enhanced Affinity and in Vivo Stability. J. Am. Chem. Soc..

[154] Liu Y., Bai X., Lyu C., Fang J., Zhang F., Wu W. H., Wei W., Zhang W.-B. (2022). Mechano-Bioconjugation
Strategy Empowering Fusion Protein Therapeutics with Aggregation Resistance,
Prolonged Circulation, and Enhanced Antitumor Efficacy. J. Am. Chem. Soc..

[155] Liu Y., Wu W. H., Hong S., Fang J., Zhang F., Liu G. X., Seo J., Zhang W.-B. (2020). Lasso Proteins:
Modular Design, Cellular Synthesis, and Topological Transformation. Angew. Chem., Int. Ed..

[156] Yang Z., Kou S., Wei X., Zhang F., Li F., Wang X. W., Lin Y., Wan C., Zhang W.-B., Sun F. (2018). Genetically Programming Stress-Relaxation Behavior in Entirely Protein-Based
Molecular Networks. ACS Macro Lett..

[157] Xu T., Sun Y., Wang Y. X., Jiang F., Hou B., Meng Z., Xu L., Liu Y., Wu W. H., Zhang W.-B. (2025). A High Modulus, Multi-Stimuli Responsive,
Interwoven
Protein Network With Topologically Confined Micro-Association. Angew. Chem., Int. Ed..

[158] Xu L., Deng P., Gao H., Zhang W.-B. (2024). Systematic Discovery
and Feature Analysis of Intertwined Symmetric Protein Motifs for Topology
Engineering. Giant.

[159] Deng P., Zhang Y., Xu L., Lyu J., Li L., Sun F., Zhang W.-B., Gao H. (2025). Computational Discovery
and Systematic Analysis of Protein Entangling Motifs in Nature: From
Algorithm to Database. Chem. Sci..

[160] Hou Y., Xie T., He L., Tao L., Huang J. (2023). Topological
Links in Predicted Protein Complex Structures Reveal Limitations of
AlphaFold. Commun. Biol..

[161] Deng P., Xu L., Wei Y., Sun F., Li L., Zhang W.-B., Gao H. (2025). Deep Learning-Assisted Discovery
of Protein Entangling Motifs. Biomacromolecules.

[162] Xu L., Tian X., Zhang W.-B. (2025). Heterodimeric
Protein Entangling
Motifs: Systematic Discovery, Feature Analysis, and Topology Engineering. Chem. Sci..

[163] Fang J., Li T., Lee J., Im D., Xu L., Liu Y., Seo J., Zhang W.-B. (2023). A Single-Domain
Protein Catenane of Dihydrofolate Reductase. Natl. Sci. Rev..

[164] Qu Z., Fang J., Wang Y. X., Sun Y., Liu Y., Wu W. H., Zhang W.-B. (2023). A Single-Domain
Green Fluorescent
Protein Catenane. Nat. Commun..

[165] Qu Z., Xu L., Jiang F., Liu Y., Zhang W.-B. (2024). Folds from
Fold: Exploring Topological Isoforms of a Single-Domain Protein. Proc. Natl. Acad. Sci. U. S. A..

[166] Da X. D., Zhang W.-B. (2019). Active Template Synthesis of Protein
Heterocatenanes. Angew. Chem., Int. Ed..

[167] Zhang F., Liu Y., Shao Y., Zhang W.-B. (2024). Active
Template Synthesis of Protein [n]­Catenanes Using Engineered Peptide-Peptide
Ligation Tools. CCS Chem..

[168] Zhang F., Liu Y., Da X. D., Zhang W.-B. (2024). Toward
Selective Synthesis of Protein Olympiadanes via Orthogonal Active
Templates in One Step. CCS Chem..

[169] Lee J., Im D., Liu Y., Fang J., Tian X., Kim M., Zhang W.-B., Seo J. (2023). Distinguishing Protein Chemical Topologies
Using Supercharging Ion Mobility Spectrometry-Mass Spectrometry. Angew. Chem., Int. Ed..

[170] Allen C. D., Link A. J. (2016). Self-Assembly of Catenanes from Lasso
Peptides. J. Am. Chem. Soc..

[171] Schröder H. V., Zhang Y., Link A. J. (2021). Dynamic
Covalent
Self-Assembly of Mechanically Interlocked Molecules Solely Made from
Peptides. Nat. Chem..

[172] Dietrich-Buchecker C.
O., Sauvage J. P., Kintzinger J. P. (1983). Une Nouvelle
Famille de Molecules: Les Metallo-Catenanes. Tetrahedron Lett..

[173] Ashton P. R., Goodnow T. T., Kaifer A. E., Reddington M. V., Slawin A. M. Z., Spencer N., Stoddart J. F., Vicent C., Williams D. J. (1989). A [2] Catenane Made to Order. Angew. Chem., Int. Ed. Engl..

[174] Gil-Ramírez G., Leigh D. A., Stephens A. J. (2015). Catenanes: Fifty
Years of Molecular Links. Angew. Chem., Int.
Ed..

[175] Frisch H. L., Wasserman E. (1961). Chemical Topology. J. Am. Chem. Soc..

[176] Au-Yeung H. Y., Deng Y. (2022). Distinctive Features and Challenges
in Catenane Chemistry. Chem. Sci..

[177] Aucagne V., Hänni K. D., Leigh D. A., Lusby P. J., Walker D. B. (2006). Catalytic “Click”
Rotaxanes: A Substoichiometric
Metal-Template Pathway to Mechanically Interlocked Architectures. J. Am. Chem. Soc..

[178] Denis M., Goldup S. M. (2017). The Active Template Approach to Interlocked
Molecules. Nat. Rev. Chem..

[179] Liu Y., Bonvallet P. A., Vignon S. A., Khan S. I., Stoddart J. F. (2005). Donor-Acceptor
Pretzelanes and a Cyclic Bis[2]­Catenane Homologue. Angew. Chem., Int. Ed..

[180] Gallagher P. R., Savoini A., Saady A., Maynard J. R. J., Butler P. W. V., Tizzard G. J., Goldup S. M. (2024). Facial Selectivity
in Mechanical Bond Formation: Axially Chiral Enantiomers and Geometric
Isomers from a Simple Prochiral Macrocycle. J. Am. Chem. Soc..

[181] Zhang M., De Bo G. (2020). A Catenane as a Mechanical Protecting
Group. J. Am. Chem. Soc..

[182] Lam R. T. S., Belenguer A., Roberts S. L., Naumann C., Jarrosson T., Otto S., Sanders J. K. M. (2005). Amplification
of Acetylcholine-Binding Catenanes from Dynamic Combinatorial Libraries. Science.

[183] Chung M. K., White P. S., Lee S. J., Gagné M. R. (2009). Synthesis
of Interlocked 56-Membered Rings by Dynamic Self-Templating. Angew. Chem., Int. Ed..

[184] Chung M. K., White P. S., Lee S. J., Waters M. L., Gagné M. R. (2012). Self-Assembled Multi-Component Catenanes: Structural
Insights into an Adaptable Class of Molecular Receptors and [2]-Catenanes. J. Am. Chem. Soc..

[185] Chung M. K., Lee S. J., Waters M. L., Gagné M. R. (2012). Self-Assembled
Multi-Component Catenanes: The Effect of Multivalency and Cooperativity
on Structure and Stability. J. Am. Chem. Soc..

[186] Cougnon F. B. L., Ponnuswamy N., Pantoş G. D., Sanders J. K. M. (2015). Molecular Motion of Donor-Acceptor
Catenanes in Water. Org. Biomol. Chem..

[187] Dehkordi M. E., Luxami V., Pantoş G. D. (2018). High-Yielding
Synthesis of Chiral Donor-Acceptor Catenanes. J. Org. Chem..

[188] Cougnon F. B. L., Ponnuswamy N., Jenkins N. A., Pantoş G. D., Sanders J. K. M. (2012). Structural Parameters Governing the Dynamic Combinatorial
Synthesis of Catenanes in Water. J. Am. Chem.
Soc..

[189] Cougnon F.
B. L., Jenkins N. A., Pantoş G. D., Sanders J. K. M. (2012). Templated Dynamic Synthesis of a
[3]­Catenane. Angew. Chem., Int. Ed..

[190] Cougnon F. B. L., Au-Yeung H. Y., Pantoş G. D., Sanders J. K. M. (2011). Exploring the Formation Pathways of Donor-Acceptor
Catenanes in Aqueous Dynamic Combinatorial Libraries. J. Am. Chem. Soc..

[191] Au-Yeung H. Y., Pantoş G. D., Sanders J. K. M. (2011). Dynamic Combinatorial
Donor-Acceptor Catenanes in Water: Access to Unconventional and Unexpected
Structures. J. Org. Chem..

[192] Au-Yeung H. Y., Pantoş G. D., Sanders J. K. M. (2010). A Water Soluble
Donor-Acceptor [2]­Catenane That Can Switch between a Coplanar and
a Gemini-Sign Conformation. Angew. Chem., Int.
Ed..

[193] Au-Yeung H. Y., Dan Pantoş G., Sanders J. K. M. (2009). Amplifying Different
[2]­Catenanes in an Aqueous Donor-Acceptor Dynamic Combinatorial Library. J. Am. Chem. Soc..

[194] Au-Yeung H. Y., Pantoş G. D., Sanders J. K. M. (2009). Dynamic Combinatorial
Synthesis of a Catenane Based on Donor-Acceptor Interactions in Water. Proc. Natl. Acad. Sci. U. S. A..

[195] Delcourt D., García Coll J., Cougnon F. B. L., Ulrich S. (2025). Effective
RNA Complexation by [2]­Catenanes Confers Enhanced Resistance to Enzymatic
Degradation. Chem. —Eur. J..

[196] Jamieson E. M. G., Modicom F., Goldup S. M. (2018). Chirality
in Rotaxanes
and Catenanes. Chem. Soc. Rev..

[197] Goldup S. M. (2024). The End of the Beginning of Mechanical
Stereochemistry. Acc. Chem. Res..

[198] Bordoli R. J., Goldup S. M. (2014). An Efficient Approach
to Mechanically
Planar Chiral Rotaxanes. J. Am. Chem. Soc..

[199] de Juan A., Lozano D., Heard A. W., Jinks M. A., Suarez J. M., Tizzard G. J., Goldup S. M. (2022). A Chiral
Interlocking
Auxiliary Strategy for the Synthesis of Mechanically Planar Chiral
Rotaxanes. Nat. Chem..

[200] Koenis M. A. J., Chibueze C. S., Jinks M. A., Nicu V. P., Visscher L., Goldup S. M., Buma W. J. (2020). Vibrational
Circular
Dichroism Spectroscopy for Probing the Expression of Chirality in
Mechanically Planar Chiral Rotaxanes. Chem.
Sci..

[201] Heard A. W., Goldup S. M. (2020). Synthesis of a Mechanically Planar
Chiral Rotaxane Ligand for Enantioselective Catalysis. Chem.

[202] Jinks M. A., de Juan A., Denis M., Fletcher C. J., Galli M., Jamieson E. M. G., Modicom F., Zhang Z., Goldup S. M. (2018). Stereoselective
Synthesis of Mechanically Planar Chiral
Rotaxanes. Angew. Chem., Int. Ed..

[203] Savoini A., Gallagher P. R., Saady A., Goldup S. M. (2024). The Final
Stereogenic Unit of [2]­Rotaxanes: Type 2 Geometric Isomers. J. Am. Chem. Soc..

[204] Denis M., Lewis J. E. M., Modicom F., Goldup S. M. (2019). An Auxiliary
Approach for the Stereoselective Synthesis of Topologically Chiral
Catenanes. Chem.

[205] Pairault N., Rizzi F., Lozano D., Jamieson E. M. G., Tizzard G. J., Goldup S. M. (2023). A Catenane That Is Topologically
Achiral despite Being Composed of Oriented Rings. Nat. Chem..

[206] Zhang S., Rodríguez-Rubio A., Saady A., Tizzard G. J., Goldup S. M. (2023). A Chiral Macrocycle
for the Stereoselective
Synthesis of Mechanically Planar Chiral Rotaxanes and Catenanes. Chem.

[207] Rodríguez-Rubio A., Savoini A., Modicom F., Butler P., Goldup S. M. (2022). A Co-Conformationally
“Topologically”
Chiral Catenane. J. Am. Chem. Soc..

[208] Thoonen S., Tuck K. L., Turner D. R. (2025). Discrete
Metallosupramolecular
Architectures with Amino Acids. Coord. Chem.
Rev..

[209] Sawada T., Fujita M. (2020). Folding and Assembly
of Metal-Linked
Peptidic Nanostructures. Chem.

[210] Dong J., Liu Y., Cui Y. (2021). Artificial
Metal-Peptide
Assemblies: Bioinspired Assembly of Peptides and Metals through Space
and across Length Scales. J. Am. Chem. Soc..

[211] Miyake R., Ando A., Ueno M., Muraoka T. (2019). Formation
of Giant and Small Cyclic Complexes from a Flexible Tripeptide Ligand
Controlled by Metal Coordination and Hydrogen Bonds. J. Am. Chem. Soc..

[212] Boer S. A., Cox R. P., Beards M. J., Wang H., Donald W. A., Bell T. D. M., Turner D. R. (2019). Elucidation of Naphthalene
Diimide Metallomacrocycles and Catenanes by Solvent Dependent Excimer
and Exciplex Emission. Chem. Commun..

[213] Sawada T., Inomata Y., Yamagami M., Fujita M. (2017). Self-Assembly
of a Peptide [2]­Catenane through Ω-Loop Folding. Chem. Lett..

[214] Tsunekawa E., Fujita M., Sawada T. (2025). A Discrete Four-Stranded
β-Sheet through Catenation of M2L2Metal-Peptide Rings. Angew. Chem., Int. Ed..

[215] Cui Z., Mu Q. S., Gao X., Jin G. X. (2023). Stereoselective
Construction of Chiral Linear [3]­Catenanes and [2]­Catenanes. J. Am. Chem. Soc..

[216] Kang X., Wang L., Liu B., Zhou S., Li Y., Yang S. L., Yao R., Qiao L., Wang X., Gong W., Liu Y., Shi L., Dong J., Cui Y., Davis A. P. (2025). Mechanically Rigid Metallopeptide Nanostructures Achieved
by Highly Efficient Folding. Nat. Synth..

[217] Schulte T. R., Holstein J. J., Schneider L., Adam A., Haberhauer G., Clever G. H. (2020). A New Mechanically-Interlocked
[Pd2L4] Cage Motif by Dimerization of Two Peptide-Based Lemniscates. Angew. Chem., Int. Ed..

[218] Hua P. P., Bai J. H., Feng H. J., Wang J. W., Zhang L. F., Jin G. X. (2024). The Topological Transformation of
41 Knot to 412 Link through Supramolecular Fusion. J. Am. Chem. Soc..

[219] Mu Q. S., Gao X., Cui Z., Lin Y. J., Jin G. X. (2023). Selective Construction of Molecular Solomon Links and
Figure-Eight Knots by Fine-Tuning Unsymmetrical Ligands. Sci. China Chem..

[220] Yang S.-L., Qiao L., Liu B., Yao R., Wang X., Gong W., Liu Y., Dong J., Davis A. P., Cui Y. (2026). Amino-Acid-Encoded Assembly of Programmable
Chiral Solomon Links. Nat. Synth..

[221] Wakayama N., Shimokawa K. (2022). On the Classification of Polyhedral
Links. Symmetry.

[222] Cui Z., Jin G. X. (2022). Construction of a Molecular Prime Link by Interlocking
Two Trefoil Knots. Nat. Synth..

[223] Cui Z., Hao L. Y., Yuan Y. F., Xuan X. P., Jin G. X. (2025). Stereoselective
Self-Assembly of a Topologically Chiral [6]­Catenane with 18 Crossings. Angew. Chem., Int. Ed..

[224] Cui Z., Gao X., Lin Y. J., Jin G. X. (2022). Stereoselective
Self-Assembly of Complex Chiral Radial [5]­Catenanes Using Half-Sandwich
Rhodium/Iridium Building Blocks. J. Am. Chem.
Soc..

[225] Sawada T., Yamagami M., Ohara K., Yamaguchi K., Fujita M. (2016). Peptide [4]­Catenane by Folding and Assembly. Angew. Chem., Int. Ed..

[226] Sawada T., Saito A., Tamiya K., Shimokawa K., Hisada Y., Fujita M. (2019). Metal-Peptide Rings Form Highly Entangled
Topologically Inequivalent Frameworks with the Same Ring- and Crossing-Numbers. Nat. Commun..

[227] Sawada T., Inomata Y., Shimokawa K., Fujita M. (2019). A Metal-Peptide Capsule by Multiple Ring Threading. Nat. Commun..

[228] Inomata Y., Oguma S., Sagara N., Nishijima A., Saburomaru Y., Yoshida S., Kajitani T., Shimokawa K., Sato S., Yoshizawa M., Fujita M., Sawada T. (2025). An M60L60
Metal-Peptide Capsid with a 60-Crossing Woven Network. Chem.

[229] McLean T. C., Balaguer-Pérez F., Chandanani J., Thomas C. M., Aicart-Ramos C., Burick S., Olinares P. D. B., Gobbato G., Mundy J. E. A., Chait B. T., Lawson D. M., Darst S. A., Campbell E. A., Moreno-Herrero F., Le T. B. K. (2025). KorB Switching from DNA-Sliding Clamp
to Repressor
Mediates Long-Range Gene Silencing in a Multi-Drug Resistance Plasmid. Nat. Microbiol..

[230] Haglund E., Sułkowska J. I., He Z., Feng G. S., Jennings P. A., Onuchic J. N. (2012). The Unique Cysteine Knot Regulates
the Pleotropic Hormone Leptin. PLoS One.

[231] Niemyska W., Dabrowski-Tumanski P., Kadlof M., Haglund E., Sułkowski P., Sulkowska J. I. (2016). Complex Lasso: New Entangled Motifs
in Proteins. Sci. Rep..

[232] Mandal K., Pentelute B. L., Bang D., Gates Z. P., Torbeev V. Y., Kent S. B. H. (2012). Design, Total Chemical Synthesis,
and X-Ray Structure of a Protein Having a Novel Linear-Loop Polypeptide
Chain Topology. Angew. Chem., Int. Ed..

[233] Greń B. A., Dabrowski-Tumanski P., Niemyska W., Sulkowska J. I. (2021). Lasso Proteins—Unifying
Cysteine Knots and Miniproteins. Polymers.

[234] Dabrowski-Tumanski P., Niemyska W., Pasznik P., Sulkowska J. I. (2016). LassoProt:
Server to Analyze Biopolymers with Lassos. Nucleic
Acids Res..

[235] Bruno da Silva F., Perlinska A. P., Płonka J., Flapan E., Sulkowska J. I. (2025). Universe
of Lasso Proteins: Exploring
the Limit of Entanglement of Proteins Predicted by AlphaFold. J. Mol. Biol..

[236] Rubach P., Płonka J., Gren B. A., Bruno
Da Silva F., Korpacz M., Sulkowska J. I. (2025). AlphaLasso—a
Web Server to Identify Loop and Lasso Motifs in 3D Structure of Biopolymers. Nucleic Acids Res..

[237] Haglund E., Pilko A., Wollman R., Jennings P. A., Onuchic J. N. (2017). Pierced Lasso Topology Controls Function in Leptin. J. Phys. Chem. B.

[238] Isaacs N. W. (1995). Cystine Knots. Curr. Opin. Struct.
Biol..

[239] Daly N. L., Craik D. J. (2011). Bioactive Cystine
Knot Proteins. Curr. Opin. Chem. Biol..

[240] Le Nguyen D., Heitz A., Chiche L., Castro B., Boigegrain R. A., Favel A., Coletti-Previero M. A. (1990). Molecular
Recognition between Serine Proteases and New Bioactive Microproteins
with a Knotted Structure. Biochimie.

[241] Postic G., Gracy J., Périn C., Chiche L., Gelly J. C. (2018). KNOTTIN: The Database of Inhibitor
Cystine Knot Scaffold after 10 Years, toward a Systematic Structure
Modeling. Nucleic Acids Res..

[242] Rees D. C., Lipscomb W. N. (1982). Refined Crystal Structure of the
Potato Inhibitor Complex of Carboxypeptidase A at 2.5 Å Resolution. J. Mol. Biol..

[243] Iyer S., Acharya K. R. (2011). Tying the Knot: The Cystine Signature
and Molecular-Recognition Processes of the Vascular Endothelial Growth
Factor Family of Angiogenic Cytokines. FEBS
J..

[244] Murray-Rust J., McDonald N. Q., Blundell T. L., Hosang M., Oefner C., Winkler F., Bradshaw R. A. (1993). Topological
Similarities
in TGF-Β2, PDGF-BB and NGF Define a Superfamily of Polypeptide
Growth Factors. Structure.

[245] McDonald N. Q., Lapatto R., Rust J. M., Gunning J., Wlodawer A., Blundell T. L. (1991). New Protein Fold
Revealed by a 2.3-Å
Resolution Crystal Structure of Nerve Growth Factor. Nature.

[246] Pallaghy P. K., Norton R. S., Nielsen K. J., Craik D. J. (1994). A Common
Structural Motif Incorporating a Cystine Knot and a Triple-stranded
Β-sheet in Toxic and Inhibitory Polypeptides. Protein Sci..

[247] Zhu S., Darbon H., Dyason K., Verdonck F., Tytgat J. (2003). Evolutionary
Origin of Inhibitor Cystine Knot Peptides. FASEB
J..

[248] Craik D. J., Daly N. L., Bond T., Waine C. (1999). Plant Cyclotides:
A Unique Family of Cyclic and Knotted Proteins That Defines the Cyclic
Cystine Knot Structural Motif. J. Mol. Biol..

[249] Flapan E., Heller G. (2015). Topological Complexity
in Protein
Structures. Comput. Math. Biophys..

[250] Craik D. J., Daly N. L., Waine C. (2001). The Cystine
Knot Motif
in Toxins and Implications for Drug Design. Toxicon.

[251] Dutertre S., Lewis R. J. (2010). Use of Venom Peptides
to Probe Ion
Channel Structure and Function. J. Biol. Chem..

[252] Góngora-Benítez M., Tulla-Puche J., Albericio F. (2014). Multifaceted Roles of Disulfide Bonds.
Peptides as
Therapeutics. Chem. Rev..

[253] Venkatesan J., Roy D. (2023). Cyclic Cystine Knot and Its Strong
Implication on the Structure and Dynamics of Cyclotides. Proteins.

[254] Colgrave M. L., Craik D. J. (2004). Thermal, Chemical, and Enzymatic
Stability of the Cyclotide Kalata B1: The Importance of the Cyclic
Cystine Knot. Biochemistry.

[255] Boisseau S., Mabrouk K., Ram N., Garmy N., Collin V., Tadmouri A., Mikati M., Sabatier J. M., Ronjat M., Fantini J., De Waard M. (2006). Cell Penetration Properties
of Maurocalcine, a Natural Venom Peptide Active on the Intracellular
Ryanodine Receptor. Biochim. Biophys. Acta,
Biomembr..

[256] Hellinger R., Muratspahić E., Devi S., Koehbach J., Vasileva M., Harvey P. J., Craik D. J., Gründemann C., Gruber C. W. (2021). Importance of the
Cyclic Cystine Knot Structural Motif
for Immunosuppressive Effects of Cyclotides. ACS Chem. Biol..

[257] De Veer S. J., Kan M. W., Craik D. J. (2019). Cyclotides: From
Structure to Function. Chem. Rev..

[258] Kan M. W., Roseli R. B., Chan L. Y., Nguyen L. T. T., Craik D. J. (2025). Recent Progress on Cyclotides: 2021–2024. ScienceAsia.

[259] Arnison P. G., Bibb M. J., Bierbaum G., Bowers A. A., Bugni T. S., Bulaj G., Camarero J. A., Campopiano D. J., Challis G. L., Clardy J., Cotter P. D., Craik D. J., Dawson M., Dittmann E., Donadio S., Dorrestein P. C., Entian K. D., Fischbach M. A., Garavelli J. S., Göransson U., Gruber C. W., Haft D. H., Hemscheidt T. K., Hertweck C., Hill C., Horswill A. R., Jaspars M., Kelly W. L., Klinman J. P., Kuipers O. P., Link A. J., Liu W., Marahiel M. A., Mitchell D. A., Moll G. N., Moore B. S., Müller R., Nair S. K., Nes I. F., Norris G. E., Olivera B. M., Onaka H., Patchett M. L., Piel J., Reaney M. J. T., Rebuffat S., Ross R. P., Sahl H. G., Schmidt E. W., Selsted M. E., Severinov K., Shen B., Sivonen K., Smith L., Stein T., Süssmuth R. D., Tagg J. R., Tang G. L., Truman A. W., Vederas J. C., Walsh C. T., Walton J. D., Wenzel S. C., Willey J. M., van der Donk W. A. (2013). Ribosomally Synthesized and Post-Translationally
Modified Peptide Natural Products: Overview and Recommendations for
a Universal Nomenclature. Nat. Prod. Rep..

[260] Saska I., Gillon A. D., Hatsugai N., Dietzgen R. G., Hara-Nishimura I., Anderson M. A., Craik D. J. (2007). An Asparaginyl
Endopeptidase
Mediates in Vivo Protein Backbone Cyclization. J. Biol. Chem..

[261] Gruber C. W., Čemažar M., Clark R. J., Horibe T., Renda R. F., Anderson M. A., Craik D. J. (2007). A Novel
Plant Protein-Disulfide Isomerase Involved in the Oxidative Folding
of Cystine Knot Defense Proteins. J. Biol. Chem..

[262] Lee M. H., Jackson M. A., Rehm F. B. H., Barkauskas D. S., Ho W. L., Yap K., Craik D. J., Gilding E. K. (2024). Proximity
Labelling Confirms the Involvement of Papain-Like Cysteine Proteases
and Chaperones in Cyclotide Biosynthesis. Plant.
Mol. Biol. Rep..

[263] Saether O., Craik D. J., Campbell I. D., Sletten K., Juul J., Norman D. G. (1995). Elucidation of the Primary and Three-Dimensional
Structure of the Uterotonic Polypeptide Kalata B1. Biochemistry.

[264] Wang C. K., King G. J., Northfield S. E., Ojeda P. G., Craik D. J. (2014). Racemic and Quasi-Racemic X-Ray Structures
of Cyclic Disulfide-Rich Peptide Drug Scaffolds. Angew. Chem., Int. Ed..

[265] Wang C. K., Shu-Hong H., Martin J. L., Sjögren T., Hajdu J., Bohlin L., Claeson P., Göransson U., Rosengren K. J., Tang J., Ning-Hua T., Craik D. J. (2009). Combined
X-Ray and NMR Analysis of the Stability of the Cyclotide Cystine Knot
Fold That Underpins Its Insecticidal Activity and Potential Use as
a Drug Scaffold. J. Biol. Chem..

[266] Akcan M., Stroud M. R., Hansen S. J., Clark R. J., Daly N. L., Craik D. J., Olson J. M. (2011). Chemical
Re-Engineering
of Chlorotoxin Improves Bioconjugation Properties for Tumor Imaging
and Targeted Therapy. J. Med. Chem..

[267] Kwon S., Bosmans F., Kaas Q., Cheneval O., Conibear A. C., Rosengren K. J., Wang C. K., Schroeder C. I., Craik D. J. (2016). Efficient Enzymatic
Cyclization of an Inhibitory Cystine
Knot-Containing Peptide. Biotechnol. Bioeng..

[268] Gran L. (1973). Oxytocic Principles of Oldenlandia
Affinis. Lloydia.

[269] Gran L., Sandberg F., Sletten K. (2000). Oldenlandia
Affinis
(R&S) DC: A Plant Containing Uteroactive Peptides Used in African
Traditional Medicine. J. Ethnopharmacol..

[270] Daly N. L., Love S., Alewood P. F., Craik D. J. (1999). Chemical
Synthesis and Folding Pathways of Large Cyclic Polypeptides: Studies
of the Cystine Knot Polypeptide Kalata B1. Biochemistry.

[271] Kintzing J. R., Cochran J. R. (2016). Engineered Knottin
Peptides as Diagnostics,
Therapeutics, and Drug Delivery Vehicles. Curr.
Opin. Chem. Biol..

[272] Wang C. K., Craik D. J. (2018). Designing Macrocyclic Disulfide-Rich
Peptides for Biotechnological Applications. Nat. Chem. Biol..

[273] Cheng X., Wu C. (2025). Directing the Oxidative Folding of
Disulfide-Rich Peptides for Enhanced Engineering and Applications. Chem. Sci..

[274] Hyun Y. (2025). Cyclotides
as Novel Plant-Derived Scaffolds for Orally Active Cyclic
Peptide Therapeutics. Mol. Cells.

[275] Gould A., Camarero J. A. (2017). Cyclotides: Overview
and Biotechnological
Applications. ChemBioChem.

[276] Deegala S., Rathnapala H. C., Rajendran S., Hettiarachchi C. (2025). Transgenic Innovation: Harnessing Cyclotides as Next
Generation Pesticides. ACS Omega.

[277] Ji Y., Majumder S., Millard M., Borra R., Bi T., Elnagar A. Y., Neamati N., Shekhtman A., Camarero J. A. (2013). In Vivo Activation of the P53 Tumor
Suppressor Pathway
by an Engineered Cyclotide. J. Am. Chem. Soc..

[278] Thongyoo P., Bonomelli C., Leatherbarrow R. J., Tate E. W. (2009). Potent Inhibitors of β-Tryptase
and Human Leukocyte
Elastase Based on the MCoTI-II Scaffold. J.
Med. Chem..

[279] Wong C. T.
T., Rowlands D. K., Wong C. H., Lo T. W. C., Nguyen G. K. T., Li H. Y., Tam J. P. (2012). Orally Active Peptidic
Bradykinin B 1 Receptor Antagonists Engineered from a Cyclotide Scaffold
for Inflammatory Pain Treatment. Angew. Chem.,
Int. Ed..

[280] Clark R. J., Daly N. L., Craik D. J. (2006). Structural Plasticity
of the Cyclic-Cystine-Knot Framework: Implications for Biological
Activity and Drug Design. Biochem. J..

[281] Li C. Y., Rehm F. B. H., Yap K., Zdenek C. N., Harding M. D., Fry B. G., Durek T., Craik D. J., de Veer S. J. (2022). Cystine Knot Peptides with Tuneable
Activity and Mechanism. Angew. Chem., Int. Ed..

[282] Cox N., Kintzing J. R., Smith M., Grant G. A., Cochran J. R. (2016). Integrin-Targeting
Knottin Peptide-Drug Conjugates Are Potent Inhibitors of Tumor Cell
Proliferation. Angew. Chem., Int. Ed..

[283] Miller C. L., Sagiv-Barfi I., Neuhöfer P., Czerwinski D. K., Artandi S. E., Bertozzi C. R., Levy R., Cochran J. R. (2022). Systemic Delivery of a Targeted Synthetic
Immunostimulant
Transforms the Immune Landscape for Effective Tumor Regression. Cell Chem. Biol..

[284] Jagadish K., Borra R., Lacey V., Majumder S., Shekhtman A., Wang L., Camarero J. A. (2013). Expression
of Fluorescent
Cyclotides Using Protein Trans-Splicing for Easy Monitoring of Cyclotide-Protein
Interactions. Angew. Chem., Int. Ed..

[285] Yap K., Du J., Rehm F. B. H., Tang S. R., Zhou Y., Xie J., Wang C. K., de Veer S. J., Lua L. H. L., Durek T., Craik D. J. (2021). Yeast-Based
Bioproduction of Disulfide-Rich Peptides
and Their Cyclization via Asparaginyl Endopeptidases. Nat. Protoc..

[286] Hansen S., Zhang Y., Hwang S., Nabhan A., Li W., Fuhrmann J., Kschonsak Y., Zhou L., Nile A. H., Gao X., Piskol R., de Sousa e Melo F., de Sauvage F. J., Hannoush R. N. (2022). Directed Evolution Identifies High-Affinity Cystine-Knot
Peptide Agonists and Antagonists of Wnt/β-Catenin Signaling. Proc. Natl. Acad. Sci. U. S. A..

[287] Liu H., Song L., Meng X., Li J., Fan S., Dong H., Wang X., Li M., Yu H., Tsai Y. H., Yin Y., Wu C. (2025). Proline-Mediated Enhancement
in Evolvability of Disulfide-Rich Peptides for Discovering Protein
Binders. J. Am. Chem. Soc..

[288] Liu W., De Veer S. J., Huang Y. H., Sengoku T., Okada C., Ogata K., Zdenek C. N., Fry B. G., Swedberg J. E., Passioura T., Craik D. J., Suga H. (2021). An Ultrapotent
and
Selective Cyclic Peptide Inhibitor of Human β-Factor XIIa in
a Cyclotide Scaffold. J. Am. Chem. Soc..

[289] Barkan D. T., Cheng X. L., Celino H., Tran T. T., Bhandari A., Craik C. S., Sali A., Smythe M. L. (2016). Clustering
of Disulfide-Rich Peptides Provides Scaffolds for Hit Discovery by
Phage Display: Application to Interleukin-23. BMC Bioinform..

[290] Maaß F., Wüstehube-Lausch J., Dickgießer S., Valldorf B., Reinwarth M., Schmoldt H. U., Daneschdar M., Avrutina O., Sahin U., Kolmar H. (2015). Cystine-Knot Peptides
Targeting Cancer-Relevant Human Cytotoxic T Lymphocyte-Associated
Antigen 4 (CTLA-4). J. Pept. Sci..

[291] Jagadish K., Gould A., Borra R., Majumder S., Mushtaq Z., Shekhtman A., Camarero J. A. (2015). Recombinant Expression
and Phenotypic Screening of a Bioactive Cyclotide Against α-Synuclein-Induced
Cytotoxicity in Baker′s Yeast. Angew.
Chem., Int. Ed..

[292] Getz J. A., Rice J. J., Daugherty P. S. (2011). Protease-Resistant
Peptide Ligands from a Knottin Scaffold Library. ACS Chem. Biol..

[293] Silverman A. P., Levin A. M., Lahti J. L., Cochran J. R. (2009). Engineered
Cystine-Knot Peptides That Bind Αvβ3 Integrin with Antibody-Like
Affinities. J. Mol. Biol..

[294] Muttenthaler M., King G. F., Adams D. J., Alewood P. F. (2021). Trends
in Peptide Drug Discovery. Nat. Rev. Drug Discovery.

[295] Maksimov M. O., Pan S. J., James
Link A. (2012). Lasso Peptides:
Structure, Function, Biosynthesis, and Engineering. Nat. Prod. Rep..

[296] Martin-Gómez H., Tulla-Puche J. (2018). Lasso Peptides: Chemical Approaches
and Structural Elucidation. Org. Biomol. Chem..

[297] Hegemann J. D., Zimmermann M., Xie X., Marahiel M. A. (2015). Lasso Peptides:
An Intriguing Class of Bacterial Natural Products. Acc. Chem. Res..

[298] Barrett S. E., Mitchell D. A. (2024). Advances in Lasso Peptide Discovery,
Biosynthesis, and Function. Trends Genet..

[299] Katahira R., Shibata K., Yamasaki M., Matsuda Y., Yoshida M. (1995). Solution Structure of Endothelin
B Receptor Selective
Antagonist RES-701–1 Determined by 1H NMR Spectroscopy. Bioorg. Med. Chem..

[300] Constantine K. L., Friedrichs M. S., Detlefsen D., Nishio M., Tsunakawa M., Furumai T., Ohkuma H., Oki T., Hill S., Bruccoleri R. E., Lin P. F., Mueller L. (1995). High-Resolution
Solution Structure of Siamycin II: Novel Amphipathic Character of
a 21-Residue Peptide That Inhibits HIV Fusion. J. Biomol. NMR.

[301] Fréchet D., Guitton J. D., Herman F., Faucher D., Helynck G., Monegier du Sorbier B., Ridoux J. P., James-Surcouf D., Vuilhorgne M. (1994). Solution Structure of RP 71955, a New 21 Amino Acid
Tricyclic Peptide Active against HIV-1 Virus. Biochemistry.

[302] Katahira R., Yamasaki M., Matsuda Y., Yoshida M. (1996). MS-271, A
Novel Inhibitor of Calmodulin-Activated Myosin Light Chain Kinase
from Streptomyces Sp.—II. Solution Structure of MS-271: Characteristic
Features of the ‘Lasso’ Structure. Bioorg. Med. Chem..

[303] Wilson K. A., Kalkum M., Ottesen J., Yuzenkova J., Chait B. T., Landick R., Muir T., Severinov K., Darst S. A. (2003). Structure of Microcin J25, a Peptide Inhibitor of Bacterial
RNA Polymerase, Is a Lassoed Tail. J. Am. Chem.
Soc..

[304] Rosengren K. J., Clark R. J., Daly N. L., Göransson U., Jones A., Craik D. J. (2003). Microcin
J25 Has a Threaded Sidechain-to-Backbone
Ring Structure and Not a Head-to-Tail Cyclized Backbone. J. Am. Chem. Soc..

[305] Bayro M. J., Mukhopadhyay J., Swapna G. V. T., Huang J. Y., Ma L. C., Sineva E., Dawson P. E., Montelione G. T., Ebright R. H. (2003). Structure of Antibacterial Peptide Microcin J25: A
21-Residue Lariat Protoknot. J. Am. Chem. Soc..

[306] Salomon R. A., Farias R. N. (1992). Microcin 25, a Novel
Antimicrobial
Peptide Produced by Escherichia coli. J. Bacteriol..

[307] Maksimov M. O., Pelczer I., Link A. J. (2012). Precursor-Centric
Genome-Mining Approach for Lasso Peptide Discovery. Proc. Natl. Acad. Sci. U. S. A..

[308] Tietz J. I., Schwalen C. J., Patel P. S., Maxson T., Blair P. M., Tai H. C., Zakai U. I., Mitchell D. A. (2017). A New Genome-Mining
Tool Redefines the Lasso Peptide Biosynthetic Landscape. Nat. Chem. Biol..

[309] Hegemann J. D., Zimmermann M., Zhu S., Klug D., Marahiel M. A. (2013). Lasso Peptides from Proteobacteria: Genome Mining Employing
Heterologous Expression and Mass Spectrometry. Biopolymers.

[310] Van Heel A. J., De Jong A., Song C., Viel J. H., Kok J., Kuipers O. P. (2018). BAGEL4: A User-Friendly
Web Server to Thoroughly Mine
RiPPs and Bacteriocins. Nucleic Acids Res..

[311] Blin K., Shaw S., Augustijn H. E., Reitz Z. L., Biermann F., Alanjary M., Fetter A., Terlouw B. R., Metcalf W. W., Helfrich E. J. N., Van
Wezel G. P., Medema M. H., Weber T. (2023). AntiSMASH 7.0: New
and Improved Predictions for Detection, Regulation, Chemical Structures
and Visualisation. Nucleic Acids Res..

[312] Cortés-Albayay C., Jarmusch S. A., Trusch F., Ebel R., Andrews B. A., Jaspars M., Asenjo J. A. (2020). Downsizing
Class II Lasso Peptides: Genome Mining-Guided Isolation of Huascopeptin
Containing the First Gly1-Asp7Macrocycle. J.
Org. Chem..

[313] Cheung-Lee W. L., Cao L., Link A. J. (2019). Pandonodin: A Proteobacterial
Lasso Peptide with an Exceptionally Long C-Terminal Tail. ACS Chem. Biol..

[314] Burkhart B. J., Hudson G. A., Dunbar K. L., Mitchell D. A. (2015). A Prevalent
Peptide-Binding Domain Guides Ribosomal Natural Product Biosynthesis. Nat. Chem. Biol..

[315] Kloosterman A. M., Shelton K. E., van Wezel G. P., Medema M. H., Mitchell D. A. (2020). RRE-Finder: A Genome-Mining Tool
for Class-Independent RiPP Discovery. mSystems.

[316] Kretsch A. M., Gadgil M. G., Dicaprio A. J., Barrett S. E., Kille B. L., Si Y., Zhu L., Mitchell D. A. (2023). Peptidase
Activation by a Leader Peptide-Bound RiPP Recognition Element. Biochemistry.

[317] Maksimov M. O., Link A. J. (2013). Discovery and Characterization of
an Isopeptidase That Linearizes Lasso Peptides. J. Am. Chem. Soc..

[318] Inokoshi J., Matsuhama M., Miyake M., Ikeda H., Tomoda H. (2012). Molecular
Cloning of the Gene Cluster for Lariatin
Biosynthesis of Rhodococcus Jostii K01-B0171. Appl. Microbiol. Biotechnol..

[319] Sumida T., Dubiley S., Wilcox B., Severinov K., Tagami S. (2019). Structural Basis of Leader Peptide Recognition in Lasso
Peptide Biosynthesis Pathway. ACS Chem. Biol..

[320] Yan K. P., Li Y., Zirah S., Goulard C., Knappe T. A., Marahiel M. A., Rebuffat S. (2012). Dissecting
the Maturation
Steps of the Lasso Peptide Microcin J25 in Vitro. ChemBioChem.

[321] Chekan J. R., Ongpipattanakul C., Nair S. K. (2019). Steric Complementarity
Directs Sequence Promiscuous Leader Binding in RiPP Biosynthesis. Proc. Natl. Acad. Sci. U. S. A..

[322] Barrett S. E., Yin S., Jordan P., Brunson J. K., Gordon-Nunez J., Costa Machado da Cruz G., Rosario C., Okada B. K., Anderson K., Pires T. A., Wang R., Shukla D., Burk M. J., Mitchell D. A. (2025). Substrate Interactions
Guide Cyclase Engineering and Lasso Peptide Diversification. Nat. Chem. Biol..

[323] Tan H. N., Liu W. Q., Ho J., Chen Y. J., Shieh F. J., Liao H. T., Wang S. P., Hegemann J. D., Chang C. Y., Chu J. (2024). Structure Prediction and Protein
Engineering Yield New Insights into Microcin J25 Precursor Recognition. ACS Chem. Biol..

[324] Koos J. D., Link A. J. (2019). Heterologous and in Vitro Reconstitution
of Fuscanodin, a Lasso Peptide from Thermobifida fusca. J. Am. Chem. Soc..

[325] Hegemann J. D., Zimmermann M., Zhu S., Steuber H., Harms K., Xie X., Marahiel M. A. (2014). Xanthomonins I-III:
A New Class of Lasso Peptides with a Seven-Residue Macrolactam Ring. Angew. Chem., Int. Ed..

[326] Cheung-Lee W. L., Parry M. E., Zong C., Cartagena A. J., Darst S. A., Connell N. D., Russo R., Link A. J. (2020). Discovery
of Ubonodin, an Antimicrobial Lasso Peptide Active against Members
of the Burkholderia Cepacia Complex. ChemBioChem.

[327] Xiu H., Wang M., Fage C. D., He Y., Niu X., Han M., Li F., An X., Fan H., Song L., Zheng G., Zhu S., Tong Y. (2022). Discovery
and Characterization
of Rubrinodin Provide Clues into the Evolution of Lasso Peptides. Biochemistry.

[328] Juarez R. J., Jiang Y., Tremblay M., Shao Q., Link A. J., Yang Z. J. (2023). LassoHTP: A High-Throughput Computational
Tool for Lasso Peptide Structure Construction and Modeling. J. Chem. Inf. Model..

[329] Ouyang X., Ran X., Xu H., Al-Abssi R., Zhao Y. L., Link A. J., Yang Z. J. (2025). LassoPred: A Tool
to Predict the 3D Structure of Lasso Peptides. Nat. Commun..

[330] Solbiati J. O., Ciaccio M., Farías R. N., Salomón R. A. (1996). Genetic Analysis of Plasmid Determinants for Microcin
J25 Production and Immunity. J. Bacteriol..

[331] Choudhury H. G., Tong Z., Mathavan I., Li Y., Iwata S., Zirah S., Rebuffat S., Van Veen H. W., Beis K. (2014). Structure of an Antibacterial Peptide ATP-Binding Cassette Transporter
in a Novel Outward Occluded State. Proc. Natl.
Acad. Sci. U. S. A..

[332] Delgado M. A., Solbiati J. O., Chiuchiolo M. J., Farías R. N., Salomón R. A. (1999). Escherichia coli Outer Membrane Protein
TolC Is Involved in Production of the Peptide Antibiotic Microcin
J25. J. Bacteriol..

[333] Bountra K., Hagelueken G., Choudhury H. G., Corradi V., El Omari K., Wagner A., Mathavan I., Zirah S., Yuan Wahlgren W., Tieleman D. P., Schiemann O., Rebuffat S., Beis K. (2017). Structural
Basis for Antibacterial
Peptide Self-immunity by the Bacterial ABC Transporter McjD. EMBO J..

[334] Li Y., Ducasse R., Zirah S., Blond A., Goulard C., Lescop E., Giraud C., Hartke A., Guittet E., Pernodet J. L., Rebuffat S. (2015). Characterization of Sviceucin from
Streptomyces Provides Insight into Enzyme Exchangeability and Disulfide
Bond Formation in Lasso Peptides. ACS Chem.
Biol..

[335] Knappe T. A., Linne U., Xie X., Marahiel M. A. (2010). The Glucagon
Receptor Antagonist BI-32169 Constitutes a New Class of Lasso Peptides. FEBS Lett..

[336] Yan Z. Y., Qu K. J., Li L. Y., Wang Z. R., He W., Guo Z. K., Tan R. X. (2025). Pseudovenmycin, a Novel Lasso Peptide
from Streptomyces Pseudovenezuelae NA07851. Tetrahedron Lett..

[337] Carson D. V., Zhang Y., So L., Cheung-Lee W. L., Cartagena A. J., Darst S. A., Link A. J. (2023). Discovery,
Characterization,
and Bioactivity of the Achromonodins: Lasso Peptides Encoded by Achromobacter. J. Nat. Prod..

[338] Xie X., Marahiel M. A. (2012). NMR as an Effective Tool for the
Structure Determination
of Lasso Peptides. ChemBioChem.

[339] Jarmusch S. A., Feldmann I., Blank-Landeshammer B., Cortés-Albayay C., Castro J. F., Andrews B., Asenjo J. A., Sickmann A., Ebel R., Jaspars M. (2020). Cutting the
Gordian Knot: Early and Complete Amino Acid Sequence Confirmation
of Class II Lasso Peptides by HCD Fragmentation. J. Antibiot. (Tokyo)..

[340] Jeanne Dit Fouque K., Lavanant H., Zirah S., Hegemann J. D., Fage C. D., Marahiel M. A., Rebuffat S., Afonso C. (2018). General Rules
of Fragmentation Evidencing Lasso Structures in CID and ETD. Analyst.

[341] Jeanne
Dit Fouque K., Afonso C., Zirah S., Hegemann J. D., Zimmermann M., Marahiel M. A., Rebuffat S., Lavanant H. (2015). Ion Mobility-Mass
Spectrometry of Lasso Peptides: Signature of a Rotaxane Topology. Anal. Chem..

[342] Dit
Fouque K. J., Moreno J., Hegemann J. D., Zirah S., Rebuffat S., Fernandez-Lima F. (2018). Identification of Lasso Peptide Topologies
Using Native Nanoelectrospray Ionization-Trapped Ion Mobility Spectrometry-Mass
Spectrometry. Anal. Chem..

[343] Nar H., Schmid A., Puder C., Potterat O. (2010). High-Resolution Crystal
Structure of a Lasso Peptide. ChemMedChem.

[344] Fage C. D., Hegemann J. D., Nebel A. J., Steinbach R. M., Zhu S., Linne U., Harms K., Bange G., Marahiel M. A. (2016). Structure
and Mechanism of the Sphingopyxin I Lasso Peptide Isopeptidase. Angew. Chem., Int. Ed..

[345] Braffman N. R., Piscotta F. J., Hauver J., Campbell E. A., Link A. J., Darst S. A. (2019). Structural Mechanism of Transcription
Inhibition by Lasso Peptides Microcin J25 and Capistruin. Proc. Natl. Acad. Sci. U. S. A..

[346] Chekan J. R., Koos J. D., Zong C., Maksimov M. O., Link A. J., Nair S. K. (2016). Structure of the Lasso Peptide Isopeptidase
Identifies a Topology for Processing Threaded Substrates. J. Am. Chem. Soc..

[347] Mathavan I., Zirah S., Mehmood S., Choudhury H. G., Goulard C., Li Y., Robinson C. V., Rebuffat S., Beis K. (2014). Structural Basis for Hijacking Siderophore Receptors by Antimicrobial
Lasso Peptides. Nat. Chem. Biol..

[348] Jangra M., Travin D. Y., Aleksandrova E. V., Kaur M., Darwish L., Koteva K., Klepacki D., Wang W., Tiffany M., Sokaribo A., Chen X., Deng Z., Tao M., Coombes B. K., Vázquez-Laslop N. (2025). A Broad-Spectrum Lasso Peptide Antibiotic Targeting the Bacterial
Ribosome. Nature.

[349] Shi J., Zhang Y., Ren W. Q., Shi Y., Wei Y. Y., Zhang B., Jiao R. H., Ge H. M. (2025). Biosynthesis
of
Collinodin Unveils Iterative Oxidative and Prenylation Modifications. ACS Catal..

[350] Shihoya W., Akasaka H., Jordan P. A., Lechner A., Okada B. K., Costa Machado da Cruz G., Sano F. K., Tanaka T., Kawahara R., Chaudhari R., Masamune H., Burk M. J., Nureki O. (2025). Structure of a Lasso
Peptide Bound ETB Receptor Provides Insights into the Mechanism of
GPCR Inverse Agonism. Nat. Commun..

[351] Duan Y., Niu W., Pang L., Bian X., Zhang Y., Zhong G. (2022). Unusual Post-Translational Modifications
in the Biosynthesis of Lasso Peptides. Int.
J. Mol. Sci..

[352] Gavrish E., Sit C. S., Cao S., Kandror O., Spoering A., Peoples A., Ling L., Fetterman A., Hughes D., Bissell A., Torrey H., Akopian T., Mueller A., Epstein S., Goldberg A., Clardy J., Lewis K. (2014). Lassomycin, a Ribosomally Synthesized
Cyclic Peptide, Kills Mycobacterium
Tuberculosis by Targeting the ATP-Dependent Protease ClpC1P1P2. Chem. Biol..

[353] Su Y., Han M., Meng X., Feng Y., Luo S., Yu C., Zheng G., Zhu S. (2019). Discovery and Characterization of
a Novel C-Terminal Peptide Carboxyl Methyltransferase in a Lassomycin-like
Lasso Peptide Biosynthetic Pathway. Appl. Microbiol.
Biotechnol..

[354] Ogawa T., Ochiai K., Tanaka T., Tsukuda E., Chiba S., Yano K., Yamasaki M., Yoshida M., Matsuda Y. (1995). RES-701–2,
−3 and −4, Novel and
Selective Endothelin Type B Receptor Antagonists Produced by Streptomyces
Sp. I. Taxonomy of Producing Strains, Fermentation, Isolation, and
Biochemical Properties. J. Antibiot. (Tokyo)..

[355] Oves-Costales D., Sánchez-Hidalgo M., Martín J., Genilloud O. (2020). Identification, Cloning and Heterologous
Expression
of the Gene Cluster Directing RES-701–3, −4 Lasso Peptides
Biosynthesis from a Marine Streptomyces Strain. Mar. Drugs.

[356] Feng Z., Ogasawara Y., Nomura S., Dairi T. (2018). Biosynthetic
Gene Cluster of a D-Tryptophan-Containing Lasso Peptide, MS-271. ChemBioChem.

[357] Feng Z., Ogasawara Y., Dairi T. (2021). Identification of the
Peptide Epimerase MslH Responsible for D-Amino Acid Introduction at
the C-Terminus of Ribosomal Peptides. Chem.
Sci..

[358] Nakashima Y., Kawakami A., Ogasawara Y., Maeki M., Tokeshi M., Dairi T., Morita H. (2023). Structure
of Lasso Peptide Epimerase MslH Reveals Metal-Dependent Acid/Base
Catalytic Mechanism. Nat. Commun..

[359] Kaweewan I., Hemmi H., Komaki H., Harada S., Kodani S. (2018). Isolation and Structure Determination of a New Lasso
Peptide Specialicin Based on Genome Mining. Bioorg. Med. Chem..

[360] Yano K., Toki S., Nakanishi S., Ochiai K., Ando K., Yoshida M., Matsuda Y., Yamasaki M. (1996). MS-271, a Novel Inhibitor of Calmodulin-Activated Myosin
Light Chain Kinase from Streptomyces Sp.—I. Isolation, Structural
Determination and Biological Properties of MS-271. Bioorg. Med. Chem..

[361] Zhang C., Seyedsayamdost M. R. (2020). CanE, an Iron/2-Oxoglutarate-Dependent
Lasso Peptide Hydroxylase from Streptomyces Canus. ACS Chem. Biol..

[362] Xu F., Wu Y., Zhang C., Davis K. M., Moon K., Bushin L. B., Seyedsayamdost M. R. (2019). A Genetics-Free
Method for High-Throughput
Discovery of Cryptic Microbial Metabolites. Nat. Chem. Biol..

[363] Um S., Jeong H., Ganley J. G., Covington B. C., Choi S., Zhang C., Kim W., Seyedsayamdost M. R., Moon K. (2026). Cebulassopins, Antiproliferative Lasso Peptides from a Split Biosynthetic
Operon. J. Am. Chem. Soc..

[364] Harris L. A., Saint-Vincent P. M. B., Guo X., Hudson G. A., Dicaprio A. J., Zhu L., Mitchell D. A. (2020). Reactivity-Based
Screening for Citrulline-Containing Natural Products Reveals a Family
of Bacterial Peptidyl Arginine Deiminases. ACS
Chem. Biol..

[365] Saad H., Majer T., Bhattarai K., Lampe S., Nguyen D. T., Kramer M., Straetener J., Brötz-Oesterhelt H., Mitchell D. A., Gross H. (2023). Bioinformatics-Guided
Discovery of Biaryl-Linked Lasso Peptides. Chem.
Sci..

[366] Harris L. A., Saad H., Shelton K. E., Zhu L., Guo X., Mitchell D. A. (2024). Tryptophan-Centric Bioinformatics Identifies New Lasso
Peptide Modifications. Biochemistry.

[367] Lu J. L., Cui J. J., Hu Z. Y., Di J. M., Li Y. Y., Xiong J., Jiao Y. M., Gao K., Min J., Luo S., Dong S. H. (2025). Characterization
of an Iterative
Halogenase Acting on Ribosomal Peptides Underlies the Combinatorial
Biosynthesis Logic of Lasso Peptides. J. Nat.
Prod..

[368] Rice A. J., Gadgil M. G., Bisignano P., Stein R. A., Mchaourab H. S., Mitchell D. A. (2025). Peptidic Tryptophan
Halogenation by a Promiscuous Flavin-Dependent Enzyme. Angew. Chem., Int. Ed..

[369] Sun K., Cui J. J., Zhai W. K., Su X., Liu Y. C., Ning L., Xiong J., Gao K., Luo S., Lei X., Dong S. H. (2025). Iterative Glycosylation on a Single Residue of a Mature
Lasso Peptide. Chem. Sci..

[370] Zyubko T., Serebryakova M., Andreeva J., Metelev M., Lippens G., Dubiley S., Severinov K. (2019). Efficient
in Vivo Synthesis of Lasso Peptide Pseudomycoidin Proceeds in the
Absence of Both the Leader and the Leader Peptidase. Chem. Sci..

[371] Shi M., Duan Y., Hou S. Y., Xie L., Sun W., Feng L., Zhang Y., Bian X., Zhong G. (2026). Functional
and Mechanistic Investigation of a Distinct DUF6895 Family Epimerase
Involved in Lasso Peptide Modification. ACS
Catal..

[372] Ma Y.-X., Di J.-M., Lu J.-L., Zhai W., Lou G.-C., Li X.-R., Cui J.-J., Xiong J., Han H., Gao K., Lei X., Luo S., Dong S.-H. (2025). Discovery
and Biosynthesis of Dehydroalanine-Containing Lasso Peptide by a Hybrid
Biosynthetic System. Org. Lett..

[373] Zhu S., Hegemann J. D., Fage C. D., Zimmermann M., Xie X., Linne U., Marahiel M. A. (2016). Insights
into the Unique Phosphorylation
of the Lasso Peptide Paeninodin. J. Biol. Chem..

[374] Zhu S., Fage C. D., Hegemann J. D., Yan D., Marahiel M. A. (2016). Dual Substrate-Controlled
Kinase Activity Leads to Polyphosphorylated Lasso Peptides. FEBS Lett..

[375] Zong C., Cheung-Lee W. L., Elashal H. E., Raj M., Link A. J. (2018). Albusnodin: An Acetylated Lasso Peptide from Streptomyces
Albus. Chem. Commun..

[376] Li Y. Y., Li X. R., Lu J. L., Cui J. J., Zhai W. K., Xiong J., Han H., Gao K., Lei X., Zhang C. R., Luo S., Dong S. H. (2025). Discovery and Biosynthesis
of Lipolasso Peptides Reveal CoA-Transferase for Tyrosine Lipidation
of Ribosomal Peptides. Org. Lett..

[377] Andersen F. D., Pedersen K. D., Wilkens
Juhl D., Mygind T., Chopin P., Svenningsen E. B., Poulsen T. B., Braad Lund M., Schramm A., Gotfredsen C. H., Tørring T. (2022). Triculamin: An Unusual Lasso Peptide with Potent Antimycobacterial
Activity. J. Nat. Prod..

[378] Merrild A., Svenningsen T., Chevrette M. G., Tørring T. (2025). Evolution-Guided Discovery of Antimycobacterial
Triculamin-Like
Lasso Peptides. Angew. Chem., Int. Ed..

[379] Jangra M., Travin D. Y., Kaur M., Hackenberger D., Koteva K., Polikanov Y. S., Wright G. D. (2026). An Acetyltransferase
Conferring Self-Resistance of the Producer to Lasso Peptide Antibiotic
Lariocidin. ACS Infect. Dis..

[380] Cao L., Beiser M., Koos J. D., Orlova M., Elashal H. E., Schröder H. V., Link A. J. (2021). Cellulonodin-2 and Lihuanodin: Lasso
Peptides with an Aspartimide Post-Translational Modification. J. Am. Chem. Soc..

[381] Cao L., Elashal H. E., Link A. J. (2023). Kinetics of Aspartimide Formation
and Hydrolysis in Lasso Peptide Lihuanodin. Biochemistry.

[382] Maksimov M. O., Koos J. D., Zong C., Lisko B., Link A. J. (2015). Elucidating the Specificity Determinants
of the AtxE2
Lasso Peptide Isopeptidase. J. Biol. Chem..

[383] Metelev M., Arseniev A., Bushin L. B., Kuznedelov K., Artamonova T. O., Kondratenko R., Khodorkovskii M., Seyedsayamdost M. R., Severinov K. (2017). Acinetodin
and Klebsidin, RNA Polymerase
Targeting Lasso Peptides Produced by Human Isolates of Acinetobacter
Gyllenbergii and Klebsiella Pneumoniae. ACS
Chem. Biol..

[384] Socias S. B., Severinov K., Salomon R. A. (2009). The Ile13 Residue
of Microcin J25 Is Essential for Recognition by the Receptor FhuA,
but Not by the Inner Membrane Transporter SbmA. FEMS Microbiol. Lett..

[385] Salomon R. A., Farias R. N. (1993). The FhuA Protein
Is Involved in Microcin
25 Uptake. J. Bacteriol..

[386] Destoumieux-Garzón D., Duquesne S., Peduzzi J., Goulard C., Desmadril M., Letellier L., Rebuffat S., Boulanger P. (2005). The Iron-Siderophore
Transporter
FhuA Is the Receptor for the Antimicrobial Peptide Microcin J25: Role
of the Microcin Val11-Pro16 β-Hairpin Region in the Recognition
Mechanism. Biochem. J..

[387] Delgado M. A., Rintoul M. R., Farías R. N., Salomón R. A. (2001). Escherichia coli RNA Polymerase Is the Target of the
Cyclopeptide Antibiotic Microcin J25. J. Bacteriol..

[388] Yuzenkova J., Delgado M., Nechaev S., Savalia D., Epshtein V., Artsimovitch I., Mooney R. A., Landick R., Farias R. N., Salomon R., Severinov K. (2002). Mutations
of Bacterial RNA Polymerase Leading to Resistance to Microcin J25. J. Biol. Chem..

[389] Kuznedelov K., Semenova E., Knappe T. A., Mukhamedyarov D., Srivastava A., Chatterjee S., Ebright R. H., Marahiel M. A., Severinov K. (2011). The Antibacterial Threaded-Lasso Peptide Capistruin
Inhibits Bacterial RNA Polymerase. J. Mol. Biol..

[390] Cheung-Lee W. L., Parry M. E., Cartagena A. J., Darst S. A., Link A. J. (2019). Discovery and Structure of the Antimicrobial
Lasso Peptide Citrocin. J. Biol. Chem..

[391] Li Y., Han Y., Zeng Z., Li W., Feng S., Cao W. (2021). Discovery and Bioactivity of the
Novel Lasso Peptide Microcin Y. J. Agric. Food
Chem..

[392] Carson D. V., Patiño M., Elashal H. E., Cartagena A. J., Zhang Y., Whitley M. E., So L., Kayser-Browne A. K., Earl A. M., Bhattacharyya R. P., Link A. J. (2023). Cloacaenodin, an
Antimicrobial Lasso Peptide with Activity against Enterobacter. ACS Infect. Dis..

[393] Do T., Thokkadam A., Leach R., Link A. J. (2022). Phenotype-Guided
Comparative Genomics Identifies the Complete Transport Pathway of
the Antimicrobial Lasso Peptide Ubonodin in Burkholderia. ACS Chem. Biol..

[394] Carson D. V., Juarez R. J., Do T., Yang Z. J., Link A. J. (2024). Antimicrobial Lasso Peptide Cloacaenodin Utilizes a
Unique TonB-Dependent Transporter to Access Susceptible Bacteria. ACS Chem. Biol..

[395] Maeda K., Kondo̅ S., Ohi K., Kondo̅ H., Wang E. L., O̅sato Y., Umezawa H. (1958). A New Antibiotic, Alboverticillin. J. Antibiot., Ser. A.

[396] Suzuki S., Asahi K., Nagatsu J., Kawashima Y., Suzuki I. (1967). Triculamin, a New Antituberculosis Substance. J. Antibiot., Ser. A.

[397] Iwatsuki M., Uchida R., Takakusagi Y., Matsumoto A., Jiang C. L., Takahashi Y., Arai M., Kobayashi S., Matsumoto M., Inokoshi J., Tomoda H., Omura S. (2007). Lariatins,
Novel Anti-Mycobacterial
Peptides with a Lasso Structure, Produced by Rhodococcus Jostii K01-B0171. J. Antibiot. (Tokyo)..

[398] Zhu S., Su Y., Shams S., Feng Y., Tong Y., Zheng G. (2019). Lassomycin
and Lariatin Lasso Peptides as Suitable Antibiotics for
Combating Mycobacterial Infections: Current State of Biosynthesis
and Perspectives for Production. Appl. Microbiol.
Biotechnol..

[399] Hegemann J. D. (2020). Factors
Governing the Thermal Stability of Lasso Peptides. ChemBioChem.

[400] Hegemann J. D., Zimmermann M., Xie X., Marahiel M. A. (2013). Caulosegnins
I-III: A Highly Diverse Group of Lasso Peptides Derived from a Single
Biosynthetic Gene Cluster. J. Am. Chem. Soc..

[401] Allen C. D., Chen M. Y., Trick A. Y., Le D. T., Ferguson A. L., Link A. J. (2016). Thermal Unthreading
of the Lasso
Peptides Astexin-2 and Astexin-3. ACS Chem.
Biol..

[402] Lear S., Munshi T., Hudson A. S., Hatton C., Clardy J., Mosely J. A., Bull T. J., Sit C. S., Cobb S. L. (2016). Total Chemical
Synthesis of Lassomycin and Lassomycin-Amide. Org. Biomol. Chem..

[403] Zimmermann M., Hegemann J. D., Xie X., Marahiel M. A. (2013). The Astexin-1
Lasso Peptides: Biosynthesis, Stability, and Structural Studies. Chem. Biol..

[404] Zong C., Wu M. J., Qin J. Z., Link A. J. (2017). Lasso Peptide
Benenodin-1 Is a Thermally Actuated [1]­Rotaxane Switch. J. Am. Chem. Soc..

[405] Schröder H. V., Stadlmeier M., Wühr M., Link A. J. (2022). The Shuttling Cascade in Lasso Peptide Benenodin-1
Is Controlled by Non-Covalent Interactions. Chem. —Eur. J..

[406] Yang Z., Hajlasz N., Kulik H. J. (2022). Computational Modeling
of Conformer Stability in Benenodin-1, a Thermally Actuated Lasso
Peptide Switch. J. Phys. Chem. B.

[407] Knappe T. A., Linne U., Robbel L., Marahiel M. A. (2009). Insights
into the Biosynthesis and Stability of the Lasso Peptide Capistruin. Chem. Biol..

[408] Ducasse R., Yan K. P., Goulard C., Blond A., Li Y., Lescop E., Guittet E., Rebuffat S., Zirah S. (2012). Sequence Determinants
Governing the Topology and Biological Activity of a Lasso Peptide,
Microcin J25. ChemBioChem.

[409] Dicaprio A. J., Firouzbakht A., Hudson G. A., Mitchell D. A. (2019). Enzymatic
Reconstitution and Biosynthetic Investigation of the Lasso Peptide
Fusilassin. J. Am. Chem. Soc..

[410] Zimmermann M., Hegemann J. D., Xie X., Marahiel M. A. (2014). Characterization
of Caulonodin Lasso Peptides Revealed Unprecedented N-Terminal Residues
and a Precursor Motif Essential for Peptide Maturation. Chem. Sci..

[411] Hegemann J. D., Fage C. D., Zhu S., Harms K., Di Leva F. S., Novellino E., Marinelli L., Marahiel M. A. (2016). The Ring Residue
Proline 8 Is Crucial for the Thermal
Stability of the Lasso Peptide Caulosegnin II. Mol. Biosyst..

[412] Zong C., Maksimov M. O., Link A. J. (2016). Construction of
Lasso Peptide Fusion Proteins. ACS Chem. Biol..

[413] Glassey E., King A. M., Anderson D. A., Zhang Z., Voigt C. A. (2022). Functional Expression of Diverse
Post-Translational
Peptide-Modifying Enzymes in Escherichia coli under Uniform Expression
and Purification Conditions. PLoS One.

[414] King A. M., Zhang Z., Glassey E., Siuti P., Clardy J., Voigt C. A. (2023). Systematic Mining
of the Human Microbiome
Identifies Antimicrobial Peptides with Diverse Activity Spectra. Nat. Microbiol..

[415] Piscotta F. J., Tharp J. M., Liu W. R., Link A. J. (2015). Expanding
the Chemical Diversity of Lasso Peptide MccJ25 with Genetically Encoded
Noncanonical Amino Acids. Chem. Commun..

[416] Al Toma R. S., Kuthning A., Exner M. P., Denisiuk A., Ziegler J., Budisa N., Süssmuth R. D. (2015). Site-Directed
and Global Incorporation of Orthogonal and Isostructural Noncanonical
Amino Acids into the Ribosomal Lasso Peptide Capistruin. ChemBioChem.

[417] Pan S. J., Rajniak J., Cheung W. L., Link A. J. (2012). Construction
of a Single Polypeptide That Matures and Exports the Lasso Peptide
Microcin J25. ChemBioChem.

[418] Zhu S., Fage C. D., Hegemann J. D., Mielcarek A., Yan D., Linne U., Marahiel M. A. (2016). The B1 Protein Guides the Biosynthesis
of a Lasso Peptide. Sci. Rep..

[419] Lechner A., Jordan P. A., Machado da Cruz G. C., Lamson J., Gordon J., Okada B. K., Anderson K., Chaudhari R., Rosario C. J., Mikesell J., McPhee S. A., Burk M. J. (2025). Overcoming
Immune Checkpoint Inhibitor Resistance with
Potent, Selective Dual Αvβ6/8 Inhibitors Based on Engineered
Lasso Peptides. J. Am. Chem. Soc..

[420] Dommaraju S. R., Kandy S. K., Ren H., Luciano D. P., Fujiki S., Sarlah D., Zhao H., Chekan J. R., Mitchell D. A. (2025). A Versatile Enzymatic Pathway for
Modification of Peptide
C-Termini. ACS Cent. Sci..

[421] Johnson T. G., Miller D. M., Carson D. V., Choi B., Link A. J. (2026). Repositioning the Leader Peptide
in Graspetide Biosynthesis. J. Am. Chem. Soc..

[422] Pavlova O., Mukhopadhyay J., Sineva E., Ebright R. H., Severinov K. (2008). Systematic Structure-Activity Analysis of Microcin
J25. J. Biol. Chem..

[423] Pan S. J., Link A. J. (2011). Sequence Diversity in the Lasso Peptide
Framework: Discovery of Functional Microcin J25 Variants with Multiple
Amino Acid Substitutions. J. Am. Chem. Soc..

[424] Hills E., Woodward T. J., Fields S., Brandsen B. M. (2022). Comprehensive
Mutational Analysis of the Lasso Peptide Klebsidin. ACS Chem. Biol..

[425] Thokkadam A., Do T., Ran X., Brynildsen M. P., Yang Z. J., Link A. J. (2023). High-Throughput Screen Reveals the
Structure-Activity Relationship of the Antimicrobial Lasso Peptide
Ubonodin. ACS Cent. Sci..

[426] Ritter S. C., Yang M. L., Kaznessis Y. N., Hackel B. J. (2018). Multispecies Activity Screening of Microcin J25 Mutants
Yields Antimicrobials with Increased Specificity toward Pathogenic
Salmonella Species Relative to Human Commensal Escherichia coli. Biotechnol. Bioeng..

[427] Kolahdoozan M., Jahanian-Najafabadi A. (2025). Lasso Peptides: A Focus on Therapeutic
Index. World J. Microbiol. Biotechnol..

[428] Knappe T. A., Manzenrieder F., Mas-Moruno C., Linne U., Sasse F., Kessler H., Xie X., Marahiel M. A. (2011). Introducing Lasso Peptides as Molecular Scaffolds for
Drug Design: Engineering of an Integrin Antagonist. Angew. Chem., Int. Ed..

[429] Hegemann J. D., De Simone M., Zimmermann M., Knappe T. A., Xie X., Di Leva F. S., Marinelli L., Novellino E., Zahler S., Kessler H., Marahiel M. A. (2014). Rational
Improvement of the Affinity and Selectivity of Integrin Binding of
Grafted Lasso Peptides. J. Med. Chem..

[430] Malik I. T., Hegemann J. D., Brötz-Oesterhelt H. (2021). Generation
of Lasso Peptide-Based ClpP Binders. Int. J.
Mol. Sci..

[431] Guarracino D. A., Carson D. V., Johnson T. G., Link A. J. (2025). Probing
Thrombosis Initiation with Lasso Peptide Variants as Inhibitors to
the von Willebrand Protein-Collagen Interaction. ChemBioChem.

[432] Duquesne S., Destoumieux-Garzón D., Zirah S., Goulard C., Peduzzi J., Rebuffat S. (2007). Two Enzymes Catalyze
the Maturation of a Lasso Peptide in Escherichia coli. Chem. Biol..

[433] Clarke D. J., Campopiano D. J. (2007). Maturation
of McjA Precursor Peptide
into Active Microcin MccJ25. Org. Biomol. Chem..

[434] Cheung W. L., Chen M. Y., Maksimov M. O., Link A. J. (2016). Lasso Peptide
Biosynthetic Protein LarB1 Binds Both Leader and Core Peptide Regions
of the Precursor Protein LarA. ACS Cent. Sci..

[435] Hegemann J. D., Schwalen C. J., Mitchell D. A., van der
Donk W. A. (2018). Elucidation of the Roles of Conserved Residues in the
Biosynthesis of the Lasso Peptide Paeninodin. Chem. Commun..

[436] Duan Y., Niu W., Pang L., Mu D. S., Du Z. J., Zhang Y., Bian X., Zhong G. (2023). Leader Peptide
Removal in Lasso Peptide Biosynthesis Based on Penultimate Isoleucine
Residue. Front. Microbiol..

[437] Rice A. J., Sword T. T., Chengan K., Mitchell D. A., Mouncey N. J., Moore S. J., Bailey C. B. (2025). Cell-Free Synthetic
Biology for Natural Product Biosynthesis and Discovery. Chem. Soc. Rev..

[438] Hunt A. C., Rasor B. J., Seki K., Ekas H. M., Warfel K. F., Karim A. S., Jewett M. C. (2025). Cell-Free Gene Expression:
Methods and Applications. Chem. Rev..

[439] Si Y., Kretsch A. M., Daigh L. M., Burk M. J., Mitchell D. A. (2021). Cell-Free
Biosynthesis to Evaluate Lasso Peptide Formation and Enzyme-Substrate
Tolerance. J. Am. Chem. Soc..

[440] Mi X., Barrett S. E., Mitchell D. A., Shukla D. (2025). LassoESM a
Tailored
Language Model for Enhanced Lasso Peptide Property Prediction. Nat. Commun..

[441] Wong D. A., Shaver Z. M., Cabezas M. D., Daniel-Ivad M., Warfel K. F., Prasanna D. V., Sobol S. E., Fernandez R., Tobias F., Filip S. K., Hulbert S. W., Faull P., Nicol R., DeLisa M. P., Balskus E. P., Karim A. S., Jewett M. C. (2025). Characterizing and Engineering Post-Translational
Modifications
with High-Throughput Cell-Free Expression. Nat.
Commun..

[442] Schiefelbein K., Lang J., Schuster M., Grigglestone C. E., Striga R., Bigler L., Schuman M. C., Zerbe O., Li Y., Hartrampf N. (2024). Merging Flow
Synthesis and Enzymatic Maturation to
Expand the Chemical Space of Lasso Peptides. J. Am. Chem. Soc..

[443] Blond A., Péduzzi J., Goulard C., Chiuchiolo M. J., Barthélémy M., Prigent Y., Salomón R. A., Farías R. N., Moreno F., Rebuffat S. (1999). The Cyclic Structure
of Microcin J25, a 21-Residue Peptide Antibiotic from Escherichia
coli. Eur. J. Biochem..

[444] Blond A., Cheminant M., Destoumieux-Garzón D., Ségalas-Milazzo I., Peduzzi J., Goulard C., Rebuffat S. (2002). Thermolysin-Linearized
Microcin J25 Retains the Structured
Core of the Native Macrocyclic Peptide and Displays Antimicrobial
Activity. Eur. J. Biochem..

[445] Rosengren K. J., Blond A., Afonso C., Tabet J. C., Rebuffat S., Craik D. J. (2004). Structure of Thermolysin
Cleaved
Microcin J25: Extreme Stability of a Two-Chain Antimicrobial Peptide
Devoid of Covalent Links. Biochemistry.

[446] Bellomio A., Vincent P. A., De Arcuri B. F., Salomón R. A., Morero R. D., Farías R. N. (2004). The Microcin
J25 β-Hairpin Region Is Important for Antibiotic Uptake but
Not for RNA Polymerase and Respiration Inhibition. Biochem. Biophys. Res. Commun..

[447] Semenova E., Yuzenkova Y., Peduzzi J., Rebuffat S., Severinov K. (2005). Structure-Activity Analysis of Microcin J25: Distinct
Parts of the Threaded Lasso Molecule Are Responsible for Interaction
with Bacterial RNA Polymerase. J. Bacteriol..

[448] Elashal H. E., Cohen R. D., Elashal H. E., Zong C., Link A. J., Raj M. (2018). Cyclic and Lasso Peptides:
Sequence
Determination, Topology Analysis, and Rotaxane Formation. Angew. Chem., Int. Ed..

[449] Bellomio A., Rintoul M. R., Morero R. D. (2003). Chemical Modification
of Microcin J25 with Diethylpyrocarbonate and Carbodiimide: Evidence
for Essential Histidyl and Carboxyl Residues. Biochem. Biophys. Res. Commun..

[450] Vincent P. A., Bellomio A., De Arcuri B. F., Farías R. N., Morero R. D. (2005). MccJ25 C-Terminal Is Involved in
RNA-Polymerase Inhibition but Not in Respiration Inhibition. Biochem. Biophys. Res. Commun..

[451] Li Y., Zhang J., Wei K., Zhou D., Wang Z., Zeng Z., Han Y., Cao W. (2024). Multi-Lasso
Peptide-Based
Synergistic Nanocomposite: A High-Stability, Broad-Spectrum Antimicrobial
Agent with Potential for Combined Antibacterial Therapy. ACS Nano.

[452] Soudy R., Etayash H., Bahadorani K., Lavasanifar A., Kaur K. (2017). Breast Cancer Targeting Peptide Binds
Keratin 1: A New Molecular Marker for Targeted Drug Delivery to Breast
Cancer. Mol. Pharmaceutics.

[453] Liu T., Ma X., Yu J., Yang W., Wang G., Wang Z., Ge Y., Song J., Han H., Zhang W., Yang D., Liu X., Ma M. (2021). Rational Generation
of Lasso Peptides Based on Biosynthetic Gene Mutations and Site-Selective
Chemical Modifications. Chem. Sci..

[454] Long T., Liu L., Tao Y., Zhang W., Quan J., Zheng J., Hegemann J. D., Uesugi M., Yao W., Tian H., Wang H. (2021). Light-Controlled
Tyrosine Nitration
of Proteins. Angew. Chem., Int. Ed..

[455] Rowe S. M., Spring D. R. (2021). The Role of Chemical
Synthesis in
Developing RiPP Antibiotics. Chem. Soc. Rev..

[456] Khatri B., Lay T., Craik D. J. (2026). Progress
and Challenges
in the Biosynthesis and Chemical Synthesis of Lasso Peptides. J. Am. Chem. Soc..

[457] Katahira R., Shibata K., Yamasaki M., Matsuda Y., Yoshida M. (1995). RES-701–1, Comparative Study of the Synthetic
and the Microbial-Origin Compounds. Bioorg.
Med. Chem. Lett..

[458] Waliczek M., Wierzbicka M., Arkuszewski M., Kijewska M., Jaremko Ł., Rajagopal P., Szczepski K., Sroczyńska A., Jaremko M., Stefanowicz P. (2020). Attempting
to Synthesize Lasso Peptides Using High Pressure. PLoS One.

[459] Harris P. W. R., Cook G. M., Leung I. K. H., Brimble M. A. (2017). An Efficient
Chemical Synthesis of Lassomycin Enabled by an On-Resin Lactamisation-Off-Resin
Methanolysis Strategy and Preparation of Chemical Variants. Aust. J. Chem..

[460] da Hora G. C. A., Oh M., Mifflin M. C., Digal L., Roberts A. G., Swanson J. M. J. (2024). Lasso Peptides: Exploring the Folding
Landscape of Nature’s Smallest Interlocked Motifs. J. Am. Chem. Soc..

[461] Nguyen J. D. M., da Hora G. C. A., Mifflin M. C., Roberts A. G., Swanson J. M. J. (2025). In Silico Design of Foldable Lasso Peptides. Biophys. J..

[462] da Hora G. C. A., Oh M., Nguyen J. D. M., Swanson J. M. J. (2024). One
Descriptor to Fold Them All: Harnessing Intuition and Machine Learning
to Identify Transferable Lasso Peptide Reaction Coordinates. J. Phys. Chem. B.

[463] Martin-Gómez H., Albericio F., Tulla-Puche J. (2018). A Lasso-Inspired
Bicyclic Peptide: Synthesis, Structure and Properties. Chem. —Eur. J..

[464] Soudy R., Wang L., Kaur K. (2012). Synthetic Peptides
Derived from the Sequence of a Lasso Peptide Microcin J25 Show Antibacterial
Activity. Bioorg. Med. Chem..

[465] Hammami R., Bédard F., Gomaa A., Subirade M., Biron E., Fliss I. (2015). Lasso-Inspired
Peptides with Distinct
Antibacterial Mechanisms. Amino Acids.

[466] Ferguson A. L., Zhang S., Dikiy I., Panagiotopoulos A. Z., Debenedetti P. G., James Link A. (2010). An Experimental
and Computational
Investigation of Spontaneous Lasso Formation in Microcin J25. Biophys. J..

[467] Li A., Zou J., Zhuo X., Chen S., Chai X., Gai C., Li X., Zhao Q., Zou Y. (2023). Rational Optimizations
of the Marine-Derived Peptide Sungsanpin as Novel Inhibitors of Cell
Invasion. Chem. Biodivers..

[468] Digal L., Samson S. C., Stevens M. A., Ghorai A., Kim H., Mifflin M. C., Carney K. R., Williamson D. L., Um S., Nagy G., Oh D. C., Mendoza M. C., Roberts A. G. (2024). Nonthreaded
Isomers of Sungsanpin and Ulleungdin Lasso Peptides Inhibit H1299
Cancer Cell Migration. ACS Chem. Biol..

[469] Chen M., Wang S., Yu X. (2019). Cryptand-Imidazolium
Supported Total Synthesis of the Lasso Peptide BI-32169 and Its D-Enantiomer. Chem. Commun..

[470] Clavel C., Fournel-Marotte K., Coutrot F. (2013). A pH-Sensitive Peptide-Containing
Lasso Molecular Switch. Molecules.

[471] Young M. J., Akien G. R., Evans N. H. (2020). An Amide
Hydrogen
Bond Templated [1]­Rotaxane Displaying a Peptide Motif-Demonstrating
an Expedient Route to Synthetic Mimics of Lasso Peptides. Org. Biomol. Chem..

[472] Saito F., Bode J. W. (2017). Synthesis and Stabilities of Peptide-Based
[1]­Rotaxanes: Molecular Grafting onto Lasso Peptide Scaffolds. Chem. Sci..

[473] Leigh D. A., Murphy A., Smart J. P., Slawin A. M. Z. (1997). Glycylglycine
Rotaxanes- The Hydrogen Bond Directed Assembly of Synthetic Peptide
Rotaxanes. Angew. Chem., Int. Ed. Engl..

[474] Clegg W., Gimenez-Saiz C., Leigh D. A., Murphy A., Slawin A. M. Z., Teat S. J. (1999). “Smart”
Rotaxanes:
Shape Memory and Control in Tertiary Amide Peptido[2]­Rotaxanes. J. Am. Chem. Soc..

[475] Biscarini F., Cavallini M., Leigh D. A., León S., Teat S. J., Wong J. K. Y., Zerbetto F. (2002). The Effect of Mechanical
Interlocking on Crystal Packing: Predictions and Testing. J. Am. Chem. Soc..

[476] Asakawa M., Brancato G., Fanti M., Leigh D. A., Shimizu T., Slawin A. M. Z., Wong J. K. Y., Zerbetto F., Zhang S. (2002). Switching
“On” and “Off” the Expression
of Chirality in Peptide Rotaxanes. J. Am. Chem.
Soc..

[477] Brancato G., Coutrot F., Leigh D. A., Murphy A., Wong J. K. Y., Zerbetto F. (2002). From Reactants
to Products via Simple
Hydrogen-Bonding Networks: Information Transmission in Chemical Reactions. Proc. Natl. Acad. Sci. U. S. A..

[478] Bottari G., Leigh D. A., Pérez E. M. (2003). Chiroptical
Switching in a Bistable Molecular Shuttle. J.
Am. Chem. Soc..

[479] Hannam J. S., Lacy S. M., Leigh D. A., Saiz C. G., Slawin A. M. Z., Stitchell S. G. (2004). Controlled Submolecular Translational
Motion in Synthesis: A Mechanically Interlocking Auxiliary. Angew. Chem., Int. Ed..

[480] Hannam J. S., Kidd T. J., Leigh D. A., Wilson A. J. (2003). “Magic
Rod” Rotaxanes: The Hydrogen Bond-Directed Synthesis of Molecular
Shuttles under Thermodynamic Control. Org. Lett..

[481] Zhou W., Chen D., Li J., Xu J., Lv J., Liu H., Li Y. (2007). Photoisomerization
of Spiropyran
for Driving a Molecular Shuttle. Org. Lett..

[482] Wurpel G. W. H., Brouwer A. M., Van Stokkum I. H. M., Farran A., Leigh D. A. (2001). Enhanced Hydrogen Bonding Induced
by Optical Excitation: Unexpected Subnanosecond Photoinduced Dynamics
in a Peptide-Based [2]­Rotaxane. J. Am. Chem.
Soc..

[483] Da Ros T., Guldi D. M., Morales A. F., Leigh D. A., Prato M., Turco R. (2003). Hydrogen Bond-Assembled
Fullerene
Molecular Shuttle. Org. Lett..

[484] Leigh D. A., Thomson A. R. (2006). Switchable Dual
Binding Mode Molecular
Shuttle. Org. Lett..

[485] Pérez E. M., Dryden D. T. F., Leigh D. A., Teobaldi G., Zerbetto F. (2004). A Generic Basis for Some Simple Light-Operated Mechanical
Molecular Machines. J. Am. Chem. Soc..

[486] Leigh D. A., Troisi A., Zerbetto F. (2000). Reducing Molecular
Shuttling to a Single Dimension. Angew. Chem.
Int. Ed.

[487] Lane A. S., Leigh D. A., Murphy A. (1997). Peptide-Based
Molecular
Shuttles. J. Am. Chem. Soc..

[488] Onagi H., Rebek J. (2005). Fluorescence Resonance
Energy Transfer across a Mechanical Bond of a Rotaxane. Chem. Commun..

[489] Martinez-Cuezva A., Valero-Moya S., Alajarin M., Berna J. (2015). Light-Responsive
Peptide [2]­Rotaxanes as Gatekeepers of Mechanised Nanocontainers. Chem. Commun..

[490] Marlin D. S., González Cabrera D., Leigh D. A., Slawin A. M. Z. (2005). Complexation-Induced
Translational Isomerism: Shuttling
through Stepwise Competitive Binding. Angew.
Chem., Int. Ed..

[491] Leigh D. A., Morales M. Á.
F., Pérez E. M., Wong J. K. Y., Saiz C. G., Slawin A. M. Z., Carmichael A. J., Haddleton D. M., Brouwer A. M., Buma W. J., Wurpel G. W. H., León S., Zerbetto F. (2005). Patterning through Controlled Submolecular
Motion: Rotaxane-Based Switches and Logic Gates That Function in Solution
and Polymer Films. Angew. Chem., Int. Ed..

[492] Moretto A., Menegazzo I., Crisma M., Shotton E. J., Nowell H., Mammi S., Toniolo C. (2009). A Rigid Helical Peptide
Axle for a [2]­Rotaxane Molecular Machine. Angew.
Chem., Int. Ed..

[493] Gatti F. G., Leigh D. A., Nepogodiev S. A., Slawin A. M. Z., Teat S. J., Wong J. K. Y. (2001). Stiff, and Sticky
in the Right Places: The Dramatic Influence of Preorganizing Guest
Binding Sites on the Hydrogen Bond-Directed Assembly of Rotaxanes. J. Am. Chem. Soc..

[494] Altieri A., Aucagne V., Carrillo R., Clarkson G. J., D’Souza D. M., Dunnett J. A., Leigh D. A., Mullen K. M. (2011). Sulfur-Containing
Amide-Based [2]­Rotaxanes and Molecular Shuttles. Chem. Sci..

[495] Zhou W., Xu J., Zheng H., Yin X., Zuo Z., Liu H., Li Y. (2009). Distinct Nanostructures from a Molecular
Shuttle: Effects of Shuttling Movement on Nanostructural Morphologies. Adv. Funct. Mater..

[496] Lin Y. H., Lai C. C., Liu Y. H., Peng S. M., Chiu S. H. (2013). Sodium Ions Template the Formation of Rotaxanes from
BPX26C6 and Nonconjugated Amide and Urea Functionalities. Angew. Chem., Int. Ed..

[497] Ramström A., Pooler D. R. S., Abasov H., Tomar M., Crespi S., Schaufelberger F. (2025). Modulation of Lanthanide Luminescence
with the Mechanical Bond: Antenna-Emitter Confinement in a Compact
[2]­Rotaxane. Angew. Chem., Int. Ed..

[498] Langton M. J., Blackburn O. A., Lang T., Faulkner S., Beer P. D. (2014). Nitrite-Templated Synthesis of Lanthanide-Containing
[2]­Rotaxanes for Anion Sensing. Angew. Chem.,
Int. Ed..

[499] Hannak R. B., Farber G., Konrat R., Krautler B. (1997). An Organometallic
B12-Rotaxane and a B12-Dimer, Relaxed and Loaded Forms of a Molecular
Spring. J. Am. Chem. Soc..

[500] Paryente S., Aledwan H., Saady A. (2025). Cyclodextrin-Based
Rotaxanes as a Versatile Platform for Biological and Medicinal Applications. Commun. Chem..

[501] Loethen S., Kim J. M., Thompson D. H. (2007). Biomedical
Applications
of Cyclodextrin Based Polyrotaxanes. Polym.
Rev..

[502] Cheetham A. G., Hutchings M. G., Claridge T. D. W., Anderson H. L. (2006). Enzymatic
Synthesis and Photoswitchable Enzymatic Cleavage of a Peptide-Linked
Rotaxane. Angew. Chem., Int. Ed..

[503] Aucagne V., Leigh D. A., Lock J. S., Thomson A. R. (2006). Rotaxanes
of Cyclic Peptides. J. Am. Chem. Soc..

[504] Kurita T., Numata K. (2025). Synthesis of Cyclic
Peptide-Based
[2]­Rotaxanes via Copper-Catalyzed Azide-Alkyne Cycloaddition. Polym. Chem..

[505] Kurita T., Gimenez-Dejoz J., Romero S., Guallar V., Numata K. (2026). Unraveling
the Impact of Cyclic Peptide Primary Structure
on Rotaxane Formation through Umbrella Sampling Molecular Dynamics
Simulations. Phys. Chem. Chem. Phys..

[506] Kurita T., Higashi M., Gimenez-Dejoz J., Fujita S., Uji H., Sato H., Numata K. (2024). Synthesis
of All-Peptide-Based Rotaxane from a Proline-Containing Cyclic Peptide. Biomacromolecules.

[507] Shinoda T., Morikawa A., Nakajima N., Hamada M., Koyama Y. (2025). Penetration of Poly­(α-Amino Acid) into a Macrocyclic
Cavity Aided by a Palladium Coordination Bond. Macromol. Chem. Phys..

[508] Puente A. R., Bessaguet A., Pairault N., Pieters G., Crassous J., Polavarapu P. L., Opalinski I., Papot S. (2021). Absolute Configuration
of a [1]­Rotaxane Determined from Vibrational
and Electronic Circular Dichroism Spectra. Chirality.

[509] Pairault N., Bessaguet A., Barat R., Frédéric L., Pieters G., Crassous J., Opalinski I., Papot S. (2021). Diastereoselective
Synthesis of [1]­Rotaxanes via an Active Metal
Template Strategy. Chem. Sci..

[510] Li A., Zhang H., Nazri N. B., Wan M., Tian C. (2025). Expanding
the Chemical Space of Mechanically Interlocked Peptides (MIPs) via
Rational Design and Metabolite Profiling. JACS
Au.

[511] Wang P. L., Chen P., Yang R., Tetlow D. J., Zhang Z. H., Han J., Fielden S. D. P., Howlader P., Zhang L., Leigh D. A. (2025). Crown Ether-Peptide
Rotaxanes. Angew. Chem., Int. Ed..

[512] Pairault N., Barat R., Tranoy-Opalinski I., Renoux B., Thomas M., Papot S. (2016). Rotaxane-Based Architectures
for Biological Applications. C. R. Chim..

[513] Dvornikovs V., House B. E., Kaetzel M., Dedman J. R., Smithrud D. B. (2003). Host-[2]­Rotaxanes as Cellular Transport
Agents. J. Am. Chem. Soc..

[514] Johnson T. G., Langton M. J. (2023). Molecular Machines For The Control
Of Transmembrane Transport. J. Am. Chem. Soc..

[515] Fernandes A., Viterisi A., Coutrot F., Potok S., Leigh D. A., Aucagne V., Papot S. (2009). Rotaxane-Based
Propeptides:
Protection and Enzymatic Release of a Bioactive Pentapeptide. Angew. Chem., Int. Ed..

[516] Fernandes A., Viterisi A., Aucagne V., Leigh D. A., Papot S. (2012). Second Generation Specific-Enzyme-Activated Rotaxane Propeptides. Chem. Commun..

[517] Barat R., Legigan T., Tranoy-Opalinski I., Renoux B., Péraudeau E., Clarhaut J., Poinot P., Fernandes A. E., Aucagne V., Leigh D. A., Papot S. (2015). A Mechanically
Interlocked Molecular System Programmed for the Delivery of an Anticancer
Drug. Chem. Sci..

[518] Lim J. Y. C., Marques I., Félix V., Beer P. D. (2017). Enantioselective
Anion Recognition by Chiral Halogen-Bonding
[2]­Rotaxanes. J. Am. Chem. Soc..

[519] David A. H. G., Casares R., Cuerva J. M., Campaña A. G., Blanco V. (2019). A [2]­Rotaxane-Based Circularly Polarized
Luminescence
Switch. J. Am. Chem. Soc..

[520] Das R. S., Mukherjee A., Kar S., Bera T., Das S., Sengupta A., Guha S. (2022). Construction of Red Fluorescent Dual
Targeting Mechanically Interlocked Molecules for Live Cancer Cell
Specific Lysosomal Staining and Multicolor Cellular Imaging. Org. Lett..

[521] Kar S., Das R. S., Bera T., Das S., Mukherjee A., Mondal A., Sengupta A., Guha S. (2025). Targeted NIR
Fluorescent
Mechanically Interlocked Molecules-Peptide Bioconjugate for Live Cancer
Cells Submitochondrial Stimulated Emission Depletion Super-Resolution
Microscopy. Bioconjugate Chem..

[522] Zhai C., Schreiber C. L., Padilla-Coley S., Oliver A. G., Smith B. D. (2020). Fluorescent Self-Threaded
Peptide
Probes for Biological Imaging. Angew. Chem.,
Int. Ed..

[523] Schreiber C. L., Zhai C., Smith B. D. (2022). Structural Engineering
of Fluorescent Self-Threaded Peptide Probes for Targeted Cell Imaging. Photochem. Photobiol..

[524] Kniazev K., Guo T., Zhai C., Gamage R. S., Ghonge S., Frantsuzov P. A., Kuno M., Smith B. (2023). Single-Molecule
Characterization of a Bright and Photostable Deep-Red Fluorescent
Squaraine-Figure-Eight (SF8) Dye. Dyes Pigm..

[525] Schreiber C. L., Zhai C., Smith B. D. (2021). Chiral Figure-Eight
Molecular Scaffold for Fluorescent Probe Development. Org. Biomol. Chem..

[526] Blanco V., Leigh D. A., Marcos V., Morales-Serna J. A., Nussbaumer A. L. (2014). A Switchable [2]­Rotaxane Asymmetric Organocatalyst
That Utilizes an Acyclic Chiral Secondary Amine. J. Am. Chem. Soc..

[527] De Bo G., Kuschel S., Leigh D. A., Lewandowski B., Papmeyer M., Ward J. W. (2014). Efficient Assembly of Threaded Molecular
Machines for Sequence-Specific Synthesis. J.
Am. Chem. Soc..

[528] Echavarren J., Gall M. A. Y., Haertsch A., Leigh D. A., Spence J. T. J., Tetlow D. J., Tian C. (2021). Sequence-Selective
Decapeptide Synthesis by the Parallel Operation of Two Artificial
Molecular Machines. J. Am. Chem. Soc..

[529] De Bo G., Gall M. A. Y., Kitching M. O., Kuschel S., Leigh D. A., Tetlow D. J., Ward J. W. (2017). Sequence-Specific
β-Peptide Synthesis by a Rotaxane-Based Molecular Machine. J. Am. Chem. Soc..

[530] De Bo G., Gall M. A. Y., Kuschel S., De Winter J., Gerbaux P., Leigh D. A. (2018). An Artificial Molecular Machine That
Builds an Asymmetric Catalyst. Nat. Nanotechnol..

[531] Caldwell S. T., Cooke G., Cooper A., Nutley M., Rabani G., Rotello V., Smith B. O., Woisel P. (2008). Tuneable Pseudorotaxane
Formation between a Biotin-Avidin Bioconjugate and CBPQT4+. Chem. Commun..

[532] Bruns C. J., Liu H., Francis M. B. (2016). Near-Quantitative
Aqueous Synthesis of Rotaxanes via Bioconjugation to Oligopeptides
and Proteins. J. Am. Chem. Soc..

[533] Klass S. H., Truxal A. E., Fiala T. A., Kelly J., Nguyen D., Finbloom J. A., Wemmer D. E., Pines A., Francis M. B. (2019). Rotaxane Probes for the Detection
of Hydrogen Peroxide
by 129Xe HyperCEST NMR Spectroscopy. Angew.
Chem., Int. Ed..

[534] d’Orchymont F., Holland J. P. (2022). Supramolecular Rotaxane-Based Multi-Modal
Probes for Cancer Biomarker Imaging. Angew.
Chem., Int. Ed..

[535] d’Orchymont F., Holland J. P. (2022). A Rotaxane-Based Platform for Tailoring
the Pharmacokinetics of Cancer-Targeted Radiotracers. Chem. Sci..

[536] Finbloom J. A., Han K., Slack C. C., Furst A. L., Francis M. B. (2017). Cucurbit­[6]­Uril-Promoted
Click Chemistry for Protein
Modification. J. Am. Chem. Soc..

[537] Slack C. C., Finbloom J. A., Jeong K., Bruns C. J., Wemmer D. E., Pines A., Francis M. B. (2017). Rotaxane
Probes
for Protease Detection by 129Xe HyperCEST NMR. Chem. Commun..

[538] Wang W., Zhou S., Yu X., Guo Q. H., Ma Y., Song J., Zhang L., Yan X., Han L., Liao Q., Li X., Zhang W.-B., Mai Y., Zhang S., Che S., Yang H. B., Fu X., Wang M. X. (2024). What Can Topology Bring to Chemistry?. CCS Chem..

[539] Guo Q. H., Jiao Y., Feng Y., Stoddart J. F. (2021). The Rise
and Promise of Molecular Nanotopology. CCS Chem..

[540] Barber B. E., Jamieson E. M. G., White L. E. M., McTernan C. T. (2025). Embracing
Complexity: Peptides as Tunable Scaffolds in the Construction of Discrete
Supramolecular Systems. Angew. Chem., Int. Ed..

[541] Ashbridge Z., Kreidt E., Pirvu L., Schaufelberger F., Stenlid J. H., Abild-Pedersen F., Leigh D. A. (2022). Vernier Template
Synthesis of Molecular Knots. Science.

[542] Bergman H. M., Fan A. T., Jones C. G., Rothenberger A. J., Jha K. K., Handford R. C., Nelson H. M., Liu Y., Tilley T. D. (2025). Discovery of an Interlocked and Interwoven Molecular
Topology in Nanocarbons via Dynamic C-C Bond Formation. J. Am. Chem. Soc..

[543] Yang Y., Ronson T. K., Teeuwen P. C. P., Zucchelli S., Heard A. W., Posocco P., Wales D. J., Nitschke J. R. (2025). Synthesis
of Covalently Linked Knotted Cage Frameworks. Nat. Synth..

[544] Zhu J., Avakyan N., Kakkis A., Hoffnagle A. M., Han K., Li Y., Zhang Z., Choi T. S., Na Y., Yu C. J., Tezcan F. A. (2021). Protein Assembly by Design. Chem. Rev..

[545] Pihl R., Zheng Q., David Y. (2023). Nature-Inspired Protein
Ligation and Its Applications. Nat. Rev. Chem..

[546] Zou Z., Kalvet I., Lozhkin B., Morris E., Zhang K., Chen D., Ernst M. L., Zhang X., Baker D., Ward T. R. (2025). De Novo Design and
Evolution of an Artificial Metathase
for Cytoplasmic Olefin Metathesis. Nat. Catal..

[547] Srisantitham S., Walker A. L., Markel U., Tezcan F. A. (2025). De Novo
Design of Proteins for Autocatalytic Isopeptide Bond Formation. J. Am. Chem. Soc..

[548] Roberts B. M. W., Del Grosso E., Penocchio E., Ricci F., Prins L. J. (2025). An Information Ratchet Improves Selectivity
in Molecular Recognition under Non-Equilibrium Conditions. Nat. Nanotechnol..

[549] Acevedo-Jake A., Ball A. T., Galli M., Kukwikila M., Denis M., Singleton D. G., Tavassoli A., Goldup S. M. (2020). AT-CuAAC Synthesis of Mechanically Interlocked Oligonucleotides. J. Am. Chem. Soc..

[550] Van Dongen S. F. M., Clerx J., Nørgaard K., Bloemberg T. G., Cornelissen J. J. L.
M., Trakselis M. A., Nelson S. W., Benkovic S. J., Rowan A. E., Nolte R. J. M. (2013). A Clamp-like
Biohybrid Catalyst for DNA Oxidation. Nat. Chem..

[551] Jiang X., Tan Y., Xie G., Lin Y., Fu W., Zhang J., Li Z., Cui X., Xiao L., Zhang C., Ji B., Deng J., Ma D., Lin Q. (2025). Polyrotaxanes Send Shuttling Signals. J. Am.
Chem. Soc..

[552] Hess H., Ross J. L. (2017). Non-Equilibrium Assembly of Microtubules:
From Molecules to Autonomous Chemical Robots. Chem. Soc. Rev..

[553] Zhao X., Palacci H., Yadav V., Spiering M. M., Gilson M. K., Butler P. J., Hess H., Benkovic S. J., Sen A. (2018). Substrate-Driven
Chemotactic Assembly in an Enzyme Cascade. Nat.
Chem..

[554] Ruijgrok P. V., Ghosh R. P., Zemsky S., Nakamura M., Gong R., Ning L., Chen R., Vachharajani V. T., Chu A. E., Anand N., Eguchi R. R., Huang P. S., Lin M. Z., Alushin G. M., Liphardt J. T., Bryant Z. (2021). Optical Control
of Fast and Processive Engineered Myosins in Vitro and in Living Cells. Nat. Chem. Biol..

[555] Rackham O., Chin J. W. (2005). A Network of Orthogonal Ribosome·mRNA
Pairs. Nat. Chem. Biol..

[556] Drexler K. E. (1999). Building Molecular Machine Systems. Trends Biotechnol..

